# Integrative pan cancer analysis reveals epigenomic variation in cancer type and cell specific chromatin domains

**DOI:** 10.1038/s41467-021-21707-1

**Published:** 2021-03-03

**Authors:** Lijin K. Gopi, Benjamin L. Kidder

**Affiliations:** 1 Department of Oncology, Wayne State University School of Medicine, Detroit, MI USA; 2 Karmanos Cancer Institute, Wayne State University School of Medicine, Detroit, MI USA

**Keywords:** Cancer genomics, Gene regulatory networks, Epigenetics, Epigenomics, Gene regulation

## Abstract

Epigenetic mechanisms contribute to the initiation and development of cancer, and epigenetic variation promotes dynamic gene expression patterns that facilitate tumor evolution and adaptation. While the NCI-60 panel represents a diverse set of human cancer cell lines that has been used to screen chemical compounds, a comprehensive epigenomic atlas of these cells has been lacking. Here, we report an integrative analysis of 60 human cancer epigenomes, representing a catalog of activating and repressive histone modifications. We identify genome-wide maps of canonical sharp and broad H3K4me3 domains at promoter regions of tumor suppressors, H3K27ac-marked conventional enhancers and super enhancers, and widespread inter-cancer and intra-cancer specific variability in H3K9me3 and H4K20me3-marked heterochromatin domains. Furthermore, we identify features of chromatin states, including chromatin state switching along chromosomes, correlation of histone modification density with genetic mutations, DNA methylation, enrichment of DNA binding motifs in regulatory regions, and gene activity and inactivity. These findings underscore the importance of integrating epigenomic maps with gene expression and genetic variation data to understand the molecular basis of human cancer. Our findings provide a resource for mining epigenomic maps of human cancer cells and for identifying epigenetic therapeutic targets.

## Abstract (web summary)

The NCI-60 cancer cell line panel covers multiple cancer types but has not been extensively investigated at the epigenetic level. Here, the authors present H3K4me3, H3K27ac, H3K9me3, and H4K20me3 ChIP-Seq analysis of the cell lines, and describe features of chromatin states and integrative analyses of expression, epigenetic and genetic mutation data.

## Introduction

Epigenetic dysregulation contributes to tumor initiation and progression, and epigenetic variation promotes dynamic gene expression patterns that facilitate tumor evolution and adaptation to therapies^1–4^. Understanding cancer type and cell type-specific epigenomes may aid in the development of new cancer diagnostic methods and personalized epigenetic therapies that target patient-specific epigenetic and transcriptional networks. The National Cancer Institute-60 (NCI-60) human pan-cancer cell line panel represents a diverse set of human cancer cell lines, which was initially developed to perform pharmacologic screens^5^. The panel consists of human cancer cell lines representing nine cell and tissue types of origin including blood, breast, colon, central nervous system, kidney, lung, ovary, prostate, and skin^6^. The widely used panel represents a resource for application of high-throughput approaches for interrogating cancer cells, and has been described at the global level by RNA-Seq transcriptome analysis^7^, whole exome sequencing^8,9^, karyotyping^10^, copy number alteration (CNA)^11^, DNA methylation^12^, proteomic profiling^13^, and metabolomics approaches^14^. However, epigenome profiling of histone modifications has not yet been applied comprehensively to cell lines represented in the NCI-60 panel.

By integrating datasets from orthogonal studies utilizing various methodologies such as next-generation sequencing technologies applied across the NCI-60 panel, they can be related to one another^15^. This type of cumulative modular analytics approach can be used to predict chemosensitivity of human cancer cells by transcriptional profiling^16,17^. Because cancer cells exhibit alterations in chromatin structure and distributions of covalent histone modifications across the genome relative to normal cells^18–20^, constructing global maps of histone modifications for the NCI-60 panel by systematic high-throughput profiling will be instrumental in annotating *cis*-regulatory elements, demarcating cancer genomes into euchromatin and heterochromatin domains, and evaluating correlations between histone modifications and gene activity, or orthogonal genetic or epigenetic features such as DNA mutation or DNA methylation, respectively.

Despite advancements in high-throughput profiling techniques, it is unclear how alterations in the epigenetic landscape contributes to cellular heterogeneity, stemness, and chemoresistance in human cancers. To understand how epigenetic patterning contributes to the biology of cancer formation and tumor progression, we systematically profiled histone modifications of 60 cancer cells from the NCI-60 panel. We integrated ChIP-Seq data generated in this study with a diversity of next-generation sequencing assays such as RNA-Seq transcriptome analysis^7^, DNA mutation analysis^8,9^, and whole genome bisulfite sequencing (WGBS)/DNA methylation^12,21^ analyses. The compiled data represents a compendium of human cancer epigenomes, which serve as a resource for the broader metastatic scientific community. Here, we report histone modification profiling (H3K4me3, H3K27ac, H3K9me3, and H4K20me3) of 60 human cancer cells representing nine distinct types of cancer in the NCI-60 panel. Genomic positional enrichment of histone modifications was used to construct an atlas of chromatin states, to functionally annotate associated genes, to identify shared and cancer type-specific features of *cis*-regulatory regions, and to identify potential upstream regulators of these states using motif-enrichment analysis. We also constructed maps of heterochromatin domains marked by the repressive histone modifications, H3K9me3 and H4K20me3, as loss of H4K20me3 was previously found to be a hallmark of cancer^22^. Our findings reveal chromatin states in cancer cells, which exhibit differences in gene activity, gene density, association with nuclear lamina, and DNA methyation. We also observed enrichment of genetic mutations in H3K4me3-enriched and H3K27ac-enriched regions, whereas H3K9me3 and H4K20me3 marked regions exhibited a reduced mutation rate. These findings provide a framework to interrogate human cancer epigenomes using histone modification data.

## Results

### Pan-cancer epigenome mapping of human cancer cells

To interrogate the global epigenetic landscapes across 9 types of cancer represented in the NCI-60 panel (Supplementary Data 1) we performed chromatin immunoprecipitation followed by next-generation sequencing (ChIP-Seq)^23,24^ to profile activating and repressive histone modifications including H3K4me3, H3K27ac, H3K9me3, and H4K20me3. H3K4me3 is predominantly enriched at promoters and transcriptional start sites (TSS) of highly expressed genes^23^, where it is presumed to serve as a platform for RNA polymerase II (RNAPII) binding and target gene activation^25–27^. H3K27ac is highly enriched at typical enhancer^28^ and super-enhancer regions^29^. Moreover, the repressive histone modifications H3K9me3 and H4K20me3, are enriched at heterochromatin regions, which are refractory to DNA-binding factors and are largely transcriptionally silent^30^. H4K20me3 is involved in the formation of heterochromatin and repression of gene expression^31^, including repetitive DNA elements^32–34^, and is involved in regulating genome stability^32^. H3K9me3 is also important for heterochromatin formation^35,36^, and is known to co-localize with H4K20me3 at heterochromatic regions^37^.

**Fig. 1:**
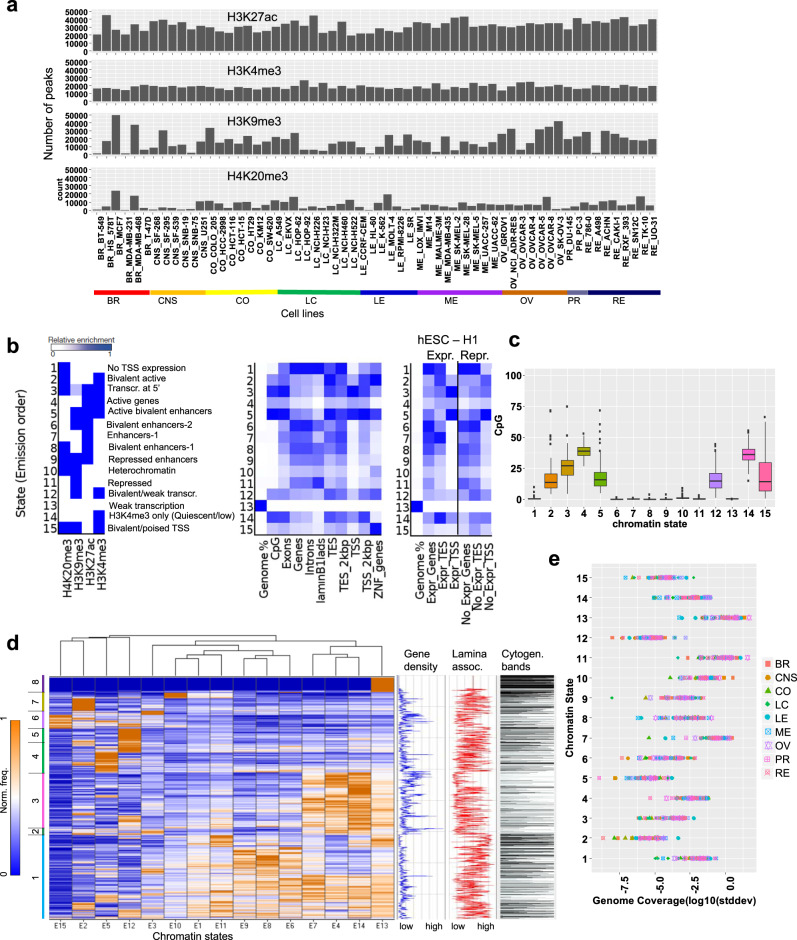
Cancer type-specific chromatin state dynamics. **a** Bar plot representation of the number of regions enriched with histone modifications (H3K4me3, H3K27ac, H3K9me3, H4K20me3) in the NCI-60 panel of human cancer cell lines. **b** Chromatin states defined by enrichment of histone modifications using ChromHMM^101^. Probabilities of histone modifications in chromatin states is depicted as a heatmap (left). Average genome coverage and annotation of genic and non-genic elements (middle). Annotation of positional expression of active and inactive genic regions in H1 ES cells (right) (TSS transcription start site, TES transcription end site). **c** Enrichment of CpG islands across *n* = 60 cancer cell lines for 15 chromatin states shows active clusters 2–5, 12, and 14, 15 relative to passive or inactive clusters 1, 6–11, and 13. Each boxplot shows CpG occupancy. Boxplots indicate the 1st and 3rd quartiles (25th and 75th percentile, upper and lower bounds), 2nd quartile (center), and minima−maxima (1.5*interquartile range, whiskers). **d** Hierarchical clustering of 2 Mb genome intervals (rows) for normalized observed vs. random relative chromatin state frequency, which was averaged across all cancer epigenomes. The gene density, cytogenic bands, and H1 ES cell LaminB1 enrichment for ES cells are depicted on the right. Hierarchical clustering heatmap: the *x*-axis shows the 15 chromatin states (E1–E15) and the *y*-axis shows the chromatin state frequency (0–1). **e** Relative chromatin state frequency for each human cancer cell epigenome. Source data are provided as a Source Data file.

Our dataset provides a global perspective of histone modifications across a wide variety of human cancer types. The comprehensive histone profiling of the NCI-60 panel of cell lines allows for multi-dimensional analyses of epigenomes across multiple types of cancer cells. Overall, we observed the greatest number of H3K27ac ChIP-enriched peaks (see the section “Methods”), followed by H3K4me3, H3K9me3, and H4K20me3 peaks (Fig. 1a). We used ChromHMM to learn chromatin states using a multivariate hidden Markov model (HMM)^38^. This approach evaluates the combined presence or absence of histone modifications to train chromatin state models. Our 15-state model identified active and inactive chromatin states with combinations of H3K4me3, H3K27ac, H3K9me3, and H4K20me3 marks (Fig. 1b). These chromatin states consist of active regions including active genes, bivalent active genes, enhancers, active bivalent enhancers, genes transcribed at the 5′ end, and bivalent/weakly transcribed. The inactive states include repressed enhancers, inactive transcription start site (TSS), heterochromatin, repressed, weak transcription, quiescent/low, and bivalent/poised TSS. Weakly transcribed and quiescent regions comprised 52% of the genome, while repressed and heterochromatin regions comprised 14% of the genome. Active enhancers were enriched at 4.4% of each reference epigenome on average, while bivalent enhancers, which were marked with H3K27ac and repressive H3K9me3 or H4K20me3 marks, were found at 11% of the genome. We also calculated the CpG island occupancy in the 15 states, and observed enrichment of CpG islands in chromatin states that are generally active including 2–5, 12, and 14–15 (Fig. 1c).

While ChromHMM defined chromatin states using a 200 bp genomic bin interval to obtain nucleosome level resolution, we further interrogated the enrichment of chromatin states across 60 cell types with lower resolution (2 Mb) to evaluate higher-order chromatin associations (Fig. 1d). Our results from these analyses show that enhancers and active genes are enriched in a fraction of the genome, whereas inactive regions comprise most of the genome (Fig. 1d). The presence of active and inactive chromatin states is consistent with previous studies reporting chromatin structural compartments^39,40^. These findings also revealed further stratification of these two compartments into regions marked by H3K27ac and H3K4me3, which were enriched across one-third of the genome (clusters 2, 3), inactive regions (clusters 1, 4, 5, 7, 8), and H3K4me3/H3K9me3/H4K20me3 bivalent regions (cluster 6). These broader regions can be further subdivided by their underlying chromatin state. The presence of active and inactive chromatin states is consistent with previous studies reporting chromatin structural compartments^39,40^.

We also evaluated gene density, LaminB1 occupancy, and cytogenetic bands for each 2 Mb interval (Fig. 1d). Heterochromatin regions are gene poor and associated with nuclear lamina. Heterochromatin and nuclear lamina are dysregulated in cancer, where nuclear lamina-associated regions exhibit increased DNA mutation frequencies relative to interlamina regions in the core of the nucleus^41^. Moreover, defective nuclear lamina and heterochromatin is associated with aneuploidy and genome instability in cancer^42^. Defects in heterochromatin are associated with tumorigenesis^43^, where de-repression of condensed chromatin can lead to structural defects such as translocations and deletions. Our findings reveal dynamic patterning of LaminB1 and cytogenetic banding across 15 chromatin states, which is consistent with variation in patterning of heterochromatin marks H3K9me3 and H4K20me3 in multiple cancer cells (Fig. 1b). In addition, we evaluated the coverage of each chromatin state across 60 epigenomes (Fig. 1e, Supplementary Fig. 1), and for each cancer type (Supplementary Fig. 2). These results reveal distinct patterns of genome coverage for 15 chromatin states across 60 cancer cell lines representing 9 types of cancer, and further reveal intra-cancer and inter-cancer heterogeneity in genome coverage (Supplementary Fig. 1). Melanoma and renal cancers were enriched with regions of no TSS expression, leukemia cells were enriched with bivalently active regions with H3K4me3/H4K20me3 and transcription at 5′ end of genes. Breast, CNS, colon, lung, and leukemia cancers were enriched with active genes more than melanoma, ovarian, prostate or renal cancers. Lung cancer and leukemia were enriched with active bivalent enhancers marked with H3K27ac/H3K4me3/H3K9me3. Breast cancer cells were enriched with enhancers, bivalent enhancers marked with H3K27ac/H3K9me3 and heterochromatin regions. Ovarian cancer was enriched with repressed chromatin regions. Melanoma cancer cells were enriched with bivalently marked H3K9me3/H3K4me3 and weak transcription regions. Breast cancer and ovarian cancer cells were enriched with weak transcription regions without H3K4me3, H3K27ac, H3K9me3, or H4K20me3 (Supplementary Figs. 1 and 2). Overall, these findings reveal the variation in combinatorial patterning of chromatin states across 60 cancer cell lines, including the overall active and inactive chromatin landscapes of 60 cancer cells, and further subdivisions into enhancer, quiescent, bivalent, weakly transcribed regions, etc. These results may help to understand cancer-specific and cell type-specific sensitivity to epigenetic drugs, where cancer cells with an aberrantly repressive or heterochromatinized chromatin landscape unable to activate tumor suppressor genes, or an overly permission chromatin landscape which may be capable of sampling various transcriptional programs, some of which may allow cancer cells to adapt to various environments or evade anti-proliferative therapies.

Next, we surveyed transcription factor-binding site (TFBS) enrichment in 15 chromatin states using data generated in human H1-ES cells and K-562 cells^44^. Coverage for TFBS was predominantly enriched in several chromatin states including 3 (transcription at 5′), 4 (active genes), 5 (active bivalent), 12 (bivalent/weak transcription), and 14 (H3K4me3 only), and to a lesser extent 2 (bivalent active; Supplementary Fig. 3a). TFBS enrichment around TSS sites was predominantly observed for chromatin states 3 and 5 (transcription at 5′, active bivalent enhancers), whereas TFBS enrichment around transcription end sites (TES) was observed for multiple states: highest TFBS enrichment was observed for chromatin states 3 and 5, followed by 2, 12, and 14 (Supplementary Fig. 3b). We also evaluated enrichment of repetitive DNA elements in the 15 chromatin states. Long interspersed elements (LINE) and long-terminal repeat (LTR) elements were enriched in chromatin states 1, 6, 8–9, 11–12, while states 2–5, 13–15 displayed decreased enrichment (Supplementary Fig. 3c). Repeat family members such as ERVK were enriched in states 1, 6, 9, 11–12 (Supplementary Fig. 3d), low complexity repeats were enriched in states 1 and 14, while RC/Helitron transposable elements (TE) were enriched in states 1–2, and 6. RNA repeats were enriched in states 2, 5 and 12, while SINE repeats were enriched in states 1, 6–8. Moreover, satellite repeats were mainly enriched in state 10, 12, and 15. In addition, an investigation of repeat subfamily members revealed dynamic organization of repeat elements in the 15 chromatin states (Supplementary Fig. 3e). Inspection of a UCSC browser view of a ChromHMM genome annotation across 60 cell lines representing 9 cancer types showed enrichment of chromatin states at a representative locus (Supplementary Fig. 3f).

To investigate enrichment of cancer genes in the 15 chromatin states, we evaluated the density of tumor suppressor genes, oncogenes, and housekeeping genes^45,46^ in 60 cancer cell lines, and across cancer types. These findings revealed heterogenous enrichment of tumor suppressor and oncogenes in several chromatin states (2–4, 12, and 14) (Supplementary Fig. 4a), and enrichment of tumor suppressors and oncogenes in chromatin states (2–5, 14) in 60 cancer cells (Supplementary Fig. 4b). Chromatin states associated with tumor suppressor and oncogenes are enriched with H3K4me3 domains (Fig. 1b).

To investigate enrichment of mutations in 15 chromatin states in 60 cancer cells and 9 cancer types, we calculated mutation density using public whole exome-sequencing data^47^. An evaluation of mutation density and subtype revealed that colon and leukemia cancer cells exhibited the greatest mutation burden (Supplementary Figs. 5a–c and 6). Variable enrichment of mutations was observed between cancer types across several chromatin states, where chromatin state 15 exhibited the highest mutation density in colon cancer cells (Supplementary Figs. 5a and 6). These findings link chromatin state domains and histone modification profiles with mutation profiles in a cancer type-specific manner, thus providing insight into the relationship between epigenetic and mutational profiles, and tumorigenesis.

**Fig. 2:**
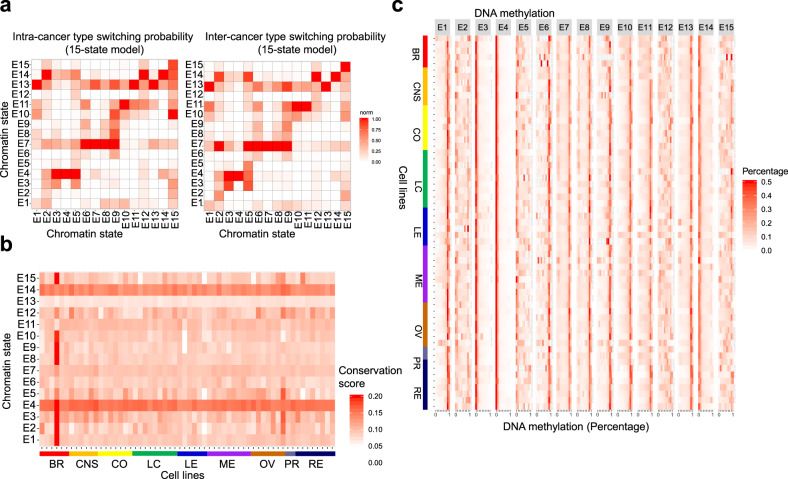
Chromatin state switching and DNA methylation in human cancer cells. **a** Intra-cancer type switching probabilities for 15 chromatin states across 60 human cancer epigenomes (left) relative to inter-cancer type switching (right). State transition (*x*-axis to *y*-axis). **b** Conservation scores for 60 epigenomes in the 15 chromatin states. **c** DNA methylation levels obtained from whole-genome bisulfite sequencing (WGBS)^9^. The percentage of methylated CpG dinucleotides is shown for the 15-state model (red, high CpG methylation). Cells from 9 types of cancer (60 cell lines) are shown on the *y*-axis and the 15 chromatin states are shown on the *x*-axis. Source data are provided as a Source Data file.

To understand the organization of chromatin states across cancer epigenomes, we surveyed chromatin state switching frequencies across all 60 cells, between samples of the same type of cancer (intra-cancer switching; Fig. 2a, left), and between samples from different types of cancer (inter-cancer switching; Fig. 2a, right). These findings revealed enrichment of switching between active and inactive chromatin states. We also observed enrichment of chromatin state switching around active enhancers and bivalently marked enhancers, which may indicate alternative patterning of enhancers in cancer genomes. In addition, we observed greater switching between states comprised of enhancers, bivalently marked enhancers, and repressed enhancers (e.g. states 5–9) between samples of the same cancer type, and between samples from different types of cancer (Fig. 2a, right). These findings reflect increased dynamic regulation of enhancer regions relative to TSS or gene body/transcribed regions. Moreover, a higher frequency of 2:7 (bivalent active:enhancers) and 5:7 (active bivalent enhancer:enhancer) state transitions were enriched more in inter-cancer relative to samples of the same type (intra-cancer).

A further analysis of chromatin switching in 9 cancer types revealed variable switching probabilities between several states (Supplementary Fig. 7). Notably, the frequency of 4:14 (active genes:quiescent) state transitions was lower in breast, colon, leukemia, and ovarian cancer relative to CNS, lung, melanoma, prostate and renal. We also observed variability in state switching 13:11 (weak transcription:repressed), 1:10 (no TSS expression:heterochromatin), and 8:1 (bivalent enhancers:no TSS expression) switching between cancer types. These findings highlight dynamic regulation of chromatin state partitioning in human cancer epigenomes, including transitions from active to repressed chromatin states. Alterations in chromatin state transitions may lead to aberrant gene activation or silencing, or genome instability, due to spreading of active or repressed chromatin domains.

Next, an investigation of enrichment of mutations relative to chromatin state transitions revealed cancer type-specific variability (Supplementary Fig. 8). We observed co-enrichment of mutations and state switching events involving repressed chromatin states 10:11 (heterochromatin:repressed) and 11:10, states 11:13 (repressed:weak transcription) and 13:11, states 14:13 (H3K4me3 only:weak transcription) and 13:14 in multiple types of cancer cells, and switching events 9:10 (repressed enhancers:heterochromatin) in lung and ovarian cancer cells (Supplementary Figs. 7 and 8). We also observed co-enrichment of mutations and chromatin switching involving active states 4:14 (active:H3K4me3 only) and 14:4 in CNS, colon, leukemia, melanoma, ovarian cancer cells, states 3:4 (transcribed 5′:active) and 4:3 in lung, melanoma, ovarian, and renal cancer cells, and states 13:14 (weak transcription:H3K4me3 only) and 14:13 in CNS and lung cancer cells. Co-enrichment of mutations and switching events was observed in chromatin states containing transcriptional enhancers 4:7 (active:enhancers) and 7:4 in lung, leukemia, melanoma, ovarian, prostate, and renal cancer cells, and states 1:8 (no TSS expr:bivalent enhancers) in breast, CNS, lung, leukemia, ovarian, and prostate cancer. Moreover, cancer type-specific co-enrichment of mutations and switching events was observed in chromatin states containing bivalently marked chromatin 3:2 (transcribed 5′:bivalent active) in colon cancer cells, states 3:15 (transcribed 5′:active bivalent enhancer) in colon, leukemia, melanoma, and renal cancer, states 12:11 (bivalent/weak:repressed) in CNS, colon, and lung cancer, states 15:10 (bivalent TSS:heterochromatin) in breast, colon, leukemia, and ovarian cancer, states 2:1 (bivalent active:no TSS expr) in CNS and melanoma cancer, and states 3:8 (transcribed 5′:bivalent enhancer) in colon and prostate cancer (Supplementary Fig. 8). In addition, we evaluated enrichment of mutation subtype relative to chromatin state switching across all cancer cell lines (Supplementary Figs. 9 and 10). These results link chromatin state transitions and histone modification profiles with mutation profiles in a cancer type and cell type-specific manner, thus providing insight into the relationship between mutational profiles and organization of repressed chromatin, active chromatin, enhancers, and bivalently marked chromatin regions in cancer.

Our model also showed increased evolutionary conservation for several chromatin states 4 and 14 including active genes and genes marked by H3K4me3 (Fig. 2b). However, regions of weak transcription without histone modifications (state 13) displayed decreased conservation (Fig. 2b). We also observed increased conservation at several chromatin states in MDA-MB-231 (E1–3, E8–10, E15) and SK-OV-3 cells (E2, E3, E5) (Fig. 2b). These findings likely reflect dysregulated patterning of H4K20me3, including depletion of these marks in heterochromatin regions and throughout the genome, but maintenance of a subset of H4K20me3 marks at genomic regions, such as promoters and exons with elevated conservation scores.

Variation in conservation between cell lines may be related to differences in combinatorial enrichment of activating and repressive histone modification patterns, where higher conservation may be observed in chromatin states of cell lines with a lower number of H4K20me3 and H3K9me3 peaks relative to other cancer cells (Fig. 1a). As activating histone modifications such as H3K4me3 and H3K27ac are associated with conserved genomic sequences while repressive histone modifications H3K9me3 and H4K20me3 are associated with non-conserved sequences^48^, depletion of H3K9me3 or H4K20me3 marks may lead to alterations in conservation of combinatorial patterning within regions that comprise chromatin states.

Integration of information about DNA methylation^9^ in our 15-state model showed that chromatin states enriched with CpG islands exhibited low DNA methylation. Our findings revealed low DNA methylation for states 3–4, 14 (transcription at 5′, active genes, and H3K4me3 only; Fig. 2c). We also found that some leukemia and melanoma cell lines displayed decreased DNA methylation across several additional chromatin states (states 6, 9–13, 15). Lower DNA methylation levels may indicate de-repression of chromatin regions. Genetic variation of DNA methyltransferases in cell lines which exhibit DNA hypomethylation, such as HL-60 and MOLT-4, may contribute to dysregulation of DNA methyltransferases. MOLT-4 exhibits pathogenic missense (substitution) mutations in the maintenance DNA methyltransferase, DNMT1 (c.2189G>A; c.4031T>C), frameshift (deletion) mutations in the de novo DNA methyltransferase DNMT3A (c.1529delG; c.2096delG), nonsense (substitution) mutations in DNMT3B (c.970C>T; c.934C>T), and missense (substitution) mutations in DNMT3L (c.721G>A; c.184C>T)^47^ (Supplementary Data 2). Moreover, HL-60 exhibits missense (substitution) mutations in DNMT3B (c.1586G>A; c.1610G>A). The combination of deleterious mutations may contribute to the DNA hypomethylation observed in HL-60 and MOLT-4 cell lines.

Misexpression of maintenance and de novo DNA methyltransferases in cancer cell lines may also contribute to altered DNA methylation. Expression of the maintenance DNA methyltransferase DNMT1 is lower in LOX IMVI melanoma cells relative to 72 percent of NCI-60 cell lines, while expression of the de novo DNA methyltransferase DNMT3A is higher in LOX IMVI cells relative to 80 percent of cell lines in the NCI-60 panel^7^ (Supplementary Data 2). Variable expression of maintenance and de novo DNA methyltransferases may contribute to dysregulated patterns of DNA methylation in cancer cells where elevated expression of de novo DNA methyltransferases may lead to aberrant DNA methylation at novel genomic sites while decreased expression of maintenance DNA methyltransferases may result in hypomethylation. Hypomethylation of DNA methyltransferases may lead to genome instability and de-repression of underlying repetitive DNA sequences, and allow cancer genomes to sample transcriptional programs, some of which may allow cancer cell to adapt and evade anti-proliferative therapies. In addition, we observed variability in DNA methylation across 60 epigenomes for several bivalent states (2, 5–6, 12). These findings reveal relationships between histone modifications, DNA methylation, and RNA transcription.

### H3K4me3 patterning distinguishes cancer epigenomes

**Fig. 3:**
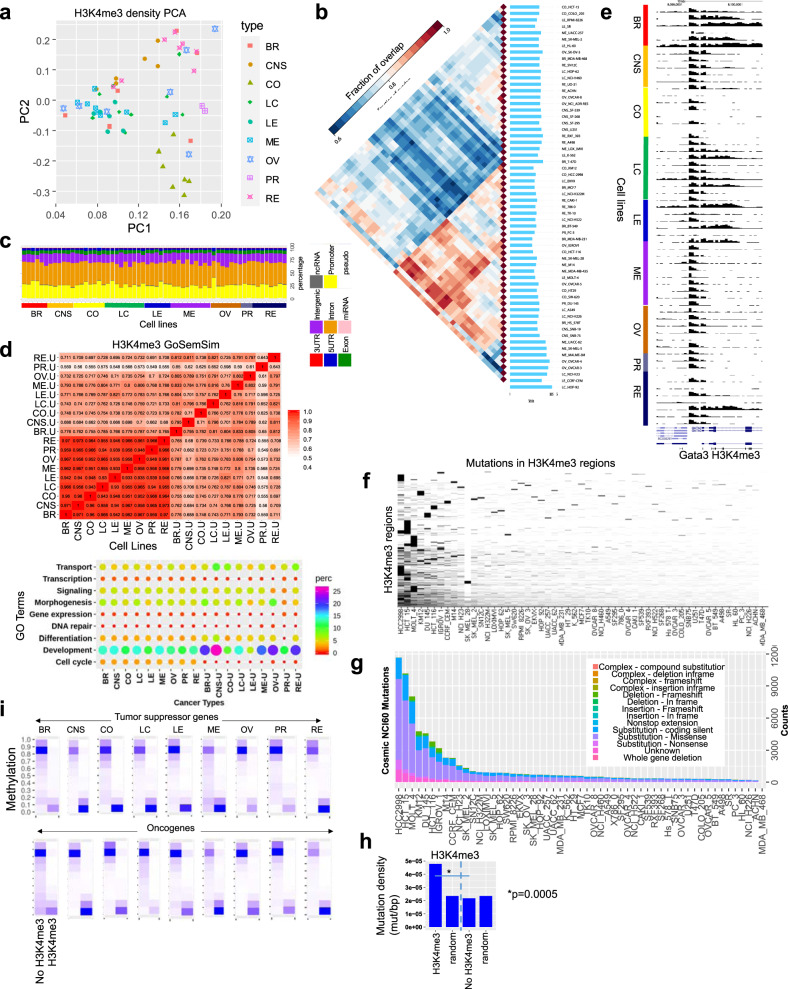
H3K4me3 dynamics and mutation analysis across 60 human cancer cell lines. **a** Principal component analysis (PCA) of H3K4me3 density levels (norm. tag density) in 60 cell lines. Nine cancer types are color coded (BR breast, CNS central nervous system, CO colon, LC lung cancer, LE leukemia, ME melanoma, OV ovary, PR prostate, RE renal). **b** Pairwise intersection of SICER^100^-defined (FDR < 0.0001) H3K4me3-enriched regions. Heat map of pairwise intersection of H3K4me3 regions was generated using Intervene^49^. **c** Genomic annotation of H3K4me3 regions in 60 cancer cell lines using HOMER^50^. **d** H3K4me3 peaks nearby TSS of genes were annotated using gene ontology (GO) functional annotation terms enriched by DAVID^102^ analysis and clustered using GoSemSim semantic similarity analysis^51^. NCBI DAVID was used to calculate *p*-values. Heatmap of semantic similarity matrix (top) and bubble plot showing enrichment of top biological process GO terms in 9 cancer types, and specific to each cancer type (u unique, bottom). **e** UCSC browser view of H3K4me3 distributions at a representative gene across 60 cancer cells. **f** Cosmic^47^ mutation analysis of H3K4me3 regions across 60 cancer cell lines. Hierarchical clustering heat map density of cosmic mutations in H3K4me3 regions. **g** Stacked bar plot showing number and type of mutation in 60 cell lines. **h** Mutation density (mutation/bp) in H3K4me3-marked regions relative to random regions of similar size and frequency, and regions without H3K4me3. *p*-value was determined using a two-sided Fisher’s exact test. **i** DNA methylation level at regions with or without H3K4me3 at tumor suppressors (top) and oncogenes (bottom). Source data are provided as a Source Data file.

To further evaluate the H3K4me3 landscape across multiple types of cancer cells, we compared H3K4me3 densities for 60 cell lines. Principal component analysis (PCA) demonstrated that colon, CNS, leukemia, prostate and renal cancer cells clustered relatively close to one another, while lung and melanoma cells were more dispersed in the 2D space, and breast and ovarian cells were more scattered (Fig. 3a). Pairwise intersections of H3Kme3 ChIP-enriched peaks (see the “Methods” section) using Intervene^49^ revealed correlations between H3K4me3 occupancy across 60 cell lines (Fig. 3b). These findings reveal relatively similar patterning of H3K4me3 marks in a subset of renal, lung, ovarian, and CNS cells, and an even greater overlap in H3K4me3 occupancy between a subset of melanoma, ovarian, breast, lung, renal, and prostate cells. This analysis also revealed significant differences between H3K4me3 occupancy across nine types of cancer cells. Annotation of H3K4me3 occupancy using HOMER^50^ revealed enrichment in active genome features, such as promoter and intron regions, followed by intergenic regions (Fig. 3c). An evaluation of overrepresented gene ontology (GO) functional annotation terms (biological process) was performed using NCBI DAVID, and further evaluated by semantic analysis of gene ontology and GoSemSim software^51^. While we observed a high correlation between GO terms enriched in all H3K4me3-occupied regions across 60 cell lines in nine types of cancer, annotation of cancer type-specific H3K4me3 peaks revealed co-enrichment of GO terms in a subset of cancer types (Fig. 3d, top, Supplementary Fig. 11; Supplementary Data 3; DAVID was used to calculate *p*-values). For example, breast and melanoma cancer-specific H3K4me3 peaks exhibited similar GO term enrichment. Moreover, enrichment of specific GO terms such as development are enriched more in cancer type-specific H3K4me3 peaks relative to all peaks (Fig. 3d, bottom). Dysregulation of developmental programs may suggest blockage of differentiation or reprogramming towards a more primitive cellular state^52,53^. As chromatin functions in part to stabilize cell fates during development, dysregulation of chromatin networks contributes to tumorigenesis. Custom views of H3K4me3 across 60 cell lines revealed alternative patterning at a representative locus, including enrichment in promoter regions and broad domains in gene body regions in breast, lung, leukemia, and renal cancer cells (Fig. 3e).

Next, we investigated whether coding variants, or mutations, in the NCI-60 panel are enriched in regions marked by H3K4me3. To this end, we calculated enrichment of genomic variants in H3K4me3 regions across 60 cell lines using public whole exome sequencing data^47^. We then clustered mutations that co-localize with H3K4me3 regions, and observed enrichment of mutations in a subset of cancer cells (Fig. 3f). An evaluation of the diversity and number of mutations showed that HCC2998, HCT15, and MOLT4 exhibited the greatest tumor mutation burden, while KM12, DU-145, HCT-116, IGROV1, M14, and CCRF-CEM also exhibited a high number of mutations (Fig. 3g). Missense substitution mutations were the most prevalent across 60 cancer cell lines. Deleterious in-frame deletion mutations were also observed in cancer cells. Next, we investigated whether mutations were enriched in H3K4me3 occupied regions or regions without H3K4me3. Our analyses demonstrated that H3K4me3-marked regions exhibit higher mutation density relative to random genomic regions of similar size and frequency, and regions without H3K4me3 (Fig. 3h). These results highlight differences in mutation densities between chromatin regions containing H3K4me3 marks, which are predominantly transcriptionally active, and chromatin regions without H3K4me3, which generally exhibit low transcriptional activity.

While a strong negative correlation is known to exist between DNA methylation and H3K4me3 enrichment at CpG islands and promoter regions^54^, and loss of H3K4me3 at promoter regions due to DNA hypermethylation, or gain of H3K4me3 due to hypomethylation occurs during malignant transformation^55^, it is unclear how dynamic regulation of H3K4me3 occurs at promoter regions without CpG islands, or at intergenic H3K4me3 regions across a diverse set of cancer cell lines. Therefore, we investigated whether there is a correlation between DNA methylation level and occurrence of H3K4me3 at tumor suppressors and oncogenes in nine types of cancers. Using a curated database of annotated tumor suppressors and oncogenes for different types of cancers^47^, overall we found that regions containing H3K4me3 exhibited lower DNA methylation levels (Fig. 3i) relative to regions without H3K4me3. In addition, we observed variability in the number of regions with high DNA methylation and without H3K4me3, and regions with low DNA methylation but with H3K4me3 occupancy in several types of cancer cells. Colon, lung, leukemia, and renal cancers exhibited relatively symmetrical counts of regions with high DNA methylation and without H3K4me3 at tumor suppressor genes, and asymmetrical counts in breast, CNS, melanoma, ovarian, and prostate cancers (Fig. 3i). However, at oncogenes, CNS and colon cancer cells exhibited relatively symmetrical counts of regions with high DNA methylation and without H3K4me3, whereas breast, lung, leukemia, melanoma, ovarian, prostate, and renal cancer cells exhibited asymmetrical counts. These findings highlight the relationship between active histone modification marking by H3K4me3 and repressive DNA methylation at tumor suppressor and oncogenes in a diverse set of cancer cells, and suggest dynamic regulation of methylation at tumor suppressors and oncogenes may lead to distinct chromatin signatures in different types of cancers, where aberrant DNA methylation of tumor suppressor or hypomethylation of oncogenes may facilitate tumor potentiation and progression. Our integrative analysis of DNA methylation and H3K4me3 profiles provide additional insight into disparate usage of H3K4me3 and DNA methylation patterning at cancer-specific genes in malignant cells.

### Broad H3K4me3 domains are associated with tumor suppressor genes in cancer cells

**Fig. 4:**
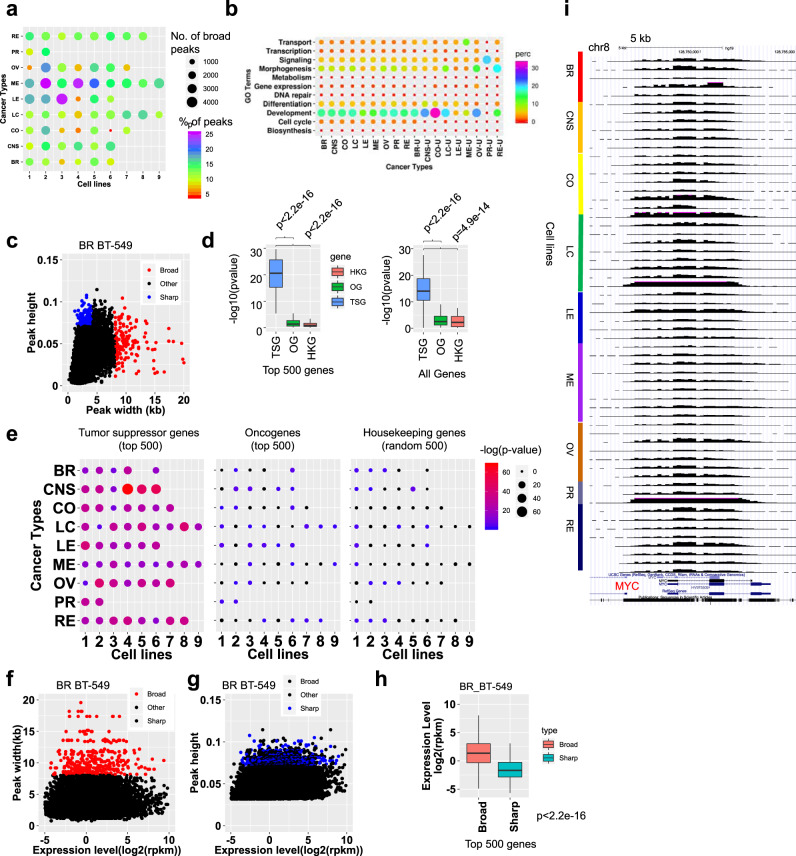
Promoter-associated broad H3K4me3 domains are associated with tumor suppressor genes. **a** Number of broad (>4 kb) H3K4me3 peaks across 60 cancer cells representing 9 types of cancer. Size of circle indicates the number of broad H3K4me3 peaks while the color indicates the percentage of total H3K4me3 peaks. **b** DAVID GO functional annotation analysis of genes associated with promoter H3K4me3 peaks. Bubble plot showing enrichment of top biological process GO terms in 9 cancer types, and specific to each cancer type (u: unique, bottom). NCBI DAVID was used to calculate *p*-values. **c** Scatter plot of H3K4me3 height (*y*-axis) and width (*x*-axis). Blue and red points represent sharp and broad peaks, respectively. **d** Boxplot of enrichment *p*-values (*y*-axis) of tumor suppressors (TSG), oncogenes (OG), and housekeeping genes for genes associated with promoter broad H3K4me3 peaks for each cancer cell line. Left: the top *n* = 500 tumor suppressors, oncogenes, and 500 random housekeeping genes were used for this analysis. Right: all (*n* = 1000) TSG, OG, and housekeeping genes were used. *p*-values (*y*-axis) were determined using two-sided Fisher’s exact tests. Boxplots indicate the 1st and 3rd quartiles (25th and 75th percentile, upper and lower bounds), 2nd quartile (center), and minima−maxima (1.5*interquartile range, whiskers). *p*-value (*x*-axis) were determined using two-sided Kolmogorov–Smirnov tests. **e** Bubble plots indicating enrichment *p*-values of TSG, OG, and housekeeping genes for genes associated with broad H3K4me3 for 60 cancer cell lines. *p*-value (−log10) represented by bubble size and color. p-values were determined using two-sided Fisher’s exact tests. Scatter plots of H3K4me3 (**f**) widths (*y*-axis) or (**g**) heights (*x*-axis) and gene expression (*x*-axis) for a representative cancer cell line. Red and blue points indicate broad and sharp peaks, respectively. **h** Boxplot showing expression level of genes associated with top *n* = 500 broad or sharp H3K4me3 peaks in a representative cancer cell line. *P* < 1 × 10^−20^ (ks-test). Boxplots indicate the 1st and 3rd quartiles (25th and 75th percentile, upper and lower bounds), 2nd quartile (center), and minima−maxima (1.5*interquartile range, whiskers). *p*-value was determined using a two-sided Kolmogorov–Smirnov test. **i** UCSC browser view of broad H3K4me3 distributions at a representative locus in 60 cancer cells (scale: 0–0.15 norm. tag density). Source data are provided as a Source Data file.

Broad H3K4me3 domains mark genes involved in cell identity^56^ and are located at tumor suppressor genes in normal cells^57^. Moreover, alterations in the length of broad domains at tumor suppressor genes (TSG) is associated with dysregulated transcription, where shortening of broad domains is associated with transcriptional repression. To interrogate the H3K4me3 breadth repertoire across multiple types of cancers, and to compare enrichment of broad H3K4me3 at TSGs, we performed a systematic analysis of H3K4me3 height and width at promoter regions in NCI60 cancer cells. We observed variable numbers of broad H3K4me3 peaks (>4 kb) and percentage of total H3K4me3 peaks for multiple types of cancer cells (Fig. 4a). Pairwise intersection analysis using Intervene software revealed a subset of broad H3K4me3 domains are shared between cancer types (Supplementary Fig. 12a, b). GO annotation of genes associated with broad H3K4me3 peaks using DAVID and GoSemSim revealed variable enrichment of multiple GO terms including development and morphogenesis in a cancer type-specific manner (Fig. 4b; Supplementary Data 3; DAVID was used to calculate *p*-values). GoSemSim analysis showed a high correlation between DAVID GO terms enriched in all broad H3K4me3-occupied regions across cells in nine types of cancer, while annotation of cancer type-specific broad H3K4me3 peaks revealed co-enrichment of GO terms in a subset of cancer types (Supplementary Fig. 12c).

We further investigated enrichment of cancer type-specific GO terms by performing hierarchical clustering of GO term *p*-values (−log10) of genes associated with all broad H3K4me3 peaks (Supplementary Fig. 13a) and genes associated with cancer type-specific broad H3K4me3 peaks (Supplementary Fig. 13b, Supplementary Data 3; DAVID was used to calculate *p*-values). These analyses reveal enrichment of GO terms in breast cancer cells (e.g. cell migration, regulation of membrane depolarization, chemotaxis, mammary gland development, neuron development, and neuron differentiation), CNS cells (e.g. neurogenesis, striated and cardiac muscle cell differentiation, synapse organization, axon and telencephalon development, cell motility and migration, blood vessel development and morphogenesis, glial differentiation, forebrain and cerebral cortex development), colon cancer cells (e.g. cellular biosynthetic process, sex differentiation, gonad development, protein glycosylation, glycoprotein biosynthetic process, stem cell proliferation, cardiovascular development), lung cancer cells (e.g. tube morphogenesis, CNS development, neuron projection development, ameboidal-type cell migration, regulation of epithelial cell migration, response to acid chemical, response to fluid shear stress), leukemia cells (e.g. hemopoiesis, immune system development, lymphocyte activation, T cell differentiation, leukocyte aggregation, lymphocyte proliferation, defense response), melanoma cells (e.g. actin cytoskeleton organization, establishment of vesicle localization¸ developmental pigmentation, neuron recognition), ovarian cancer cells (e.g. formation of primary germ layer, mesoderm formation, O-glycan processing, regulation of RNA biosynthetic process), prostate cancer cells (response to steroid hormone, hormone-mediated signaling pathway¸ cellular response to steroid hormone stimulus), and renal cancer cells (e.g. angiogenesis, response to wounding¸ regulation of cell communication¸ cilium organization) (Supplementary Data 3). While these findings provide insight into the cancer phenotype, such as melanoma cells exhibiting enrichment of pigmentation GO terms, or leukemia cells exhibiting enrichment of lymphocyte proliferation GO terms, these results also highlight distinct cellular pathways activated in various types of cancer cells.

A comparison of public ChIP-Seq data from normal cells using Intervene pairwise intersection analysis showed that broad H3K4me3 cancer type-specific patterns are mostly distinct from broad H3K4me3 patterns in normal cells (Supplementary Fig. 14). With the exception of normal blood cells and leukemia cells, variability between cancer cells and normal cells was greater than intra-cancer or intra-normal cell heterogeneity. Because broad H3K4me3 patterns are mostly distinct between cancer cells and normal cells, and heterogeneous in cancer cells, our results may suggest that cancer type-specific heterogeneity in broad H3K4me3 domain genome-wide distributions may be acquired during carcinogenesis.

We found a subset of low density H3K4me3 peaks that were wide and another subset of high density H3K4me3 peaks that were narrow (Fig. 4c; Supplementary Fig. 15). To investigate a correlation between broad H3K4me3 domains and cancer, we utilized the top 500 tumor suppressors and oncogenes, as defined by somatic mutation profiles from >8000 paired tumor-normal samples^45^. Housekeeping genes (500 random) were also used as a control^46^. Using genes associated with broad H3K4me3 peaks for each cancer cell line, we evaluated enrichment of the top 500 tumor suppressors, oncogenes, and 500 random housekeeping genes (Fig. 4d, left, Fig. 4e, Supplementary Fig. 16a, Supplementary Data 4), or all tumor suppressors, oncogenes, and housekeeping genes (Fig. 4d, right, Supplementary Fig. 16b, Supplementary Data 4). We observed greater enrichment of tumor suppressors relative to oncogenes and housekeeping genes across cell lines from distinct cancer types (Fig. 4e, Supplementary Fig. 16b). We also performed an analysis using cancer type-specific tumor suppressors and oncogenes (Supplementary Data 4), and similarly observed greater enrichment of tumor suppressors relative to oncogenes and housekeeping genes across distinct cancer types (Supplementary Fig. 17a) and for most cancer cell lines (Supplementary Fig. 17b).

Also, genes associated with broad H3K4me3 peaks (>4 kb) were expressed at a higher level relative to genes associated with sharp (<4 kb) peaks (Fig. 4f–h, Supplementary Fig. 18–20). While broad H3K4me3 domains were enriched at tumor suppressor genes, we also observed broad H3K4me3 domains at a subset of oncogenes such as *MYC* (Fig. 4i). While broad H3K4me3 domains were found at the *MYC* gene for all NCI-60 cell lines, H3K4me3 levels were variable across the NCI-60 panel (Fig. 4i). We also observed variable levels and distributions of H3K27ac nearby *MYC* broad H3K4me3 domains, including dynamic cancer type-specific patterning of intergenic H3K27ac marked enhancers (Supplementary Fig. 21), where broad H3K4me3 levels were more highly correlated with H3K27ac levels at promoter regions relative to intergenic H3K27ac levels. Broad H3K4me3 peaks at oncogenes may promote sustained expression to drive tumor potentiation or tumor progression.

As genes with conserved broad H3K4me3 peaks represent pan-cancer tumor suppressors^57^, to investigate a relationship between alterations in length of broad H3K4me3 domains and level of gene expression, we performed a systematic comparison of relative shortening or lengthening of conserved H3K4me3 domains across multiple types of cancer cells. Conserved H3K4me3 peaks that intersect TSS regions were defined as those found in more than 50% of cancer cell lines (>30 cell lines). Using a subtraction cutoff of 500 bp, we defined lengthening of H3K4me3 peaks as an increase in breadth >500 bp relative to the average breadth across 60 cancer cell lines, and shortening as a decrease in breadth less than 500 bp. Next, we evaluated the expression of tumor suppressors, oncogenes, and housekeeping genes associated with conserved H3K4me3 peaks that lengthen or shorten relative to the average. Using this approach, we found that shortening of conserved H3K4me3 domains was mostly associated with lower expression of tumor suppressors (Supplementary Fig. 22) and oncogenes (Supplementary Fig. 23), while lengthening was associated with higher or lower expression. In contract, shortening of conserved H3K4me3 domains resulted in nominal changes in expression of housekeeping genes for most cancer cells (Supplementary Fig. 24). These findings suggest that variation in length of conserved broad H3K4me3 is associated with disparate expression patterns of tumor suppressors and oncogenes across multiple types of cancer cells.

### H3K27ac enhancer profiling in a compendium of cancer cells

**Fig. 5:**
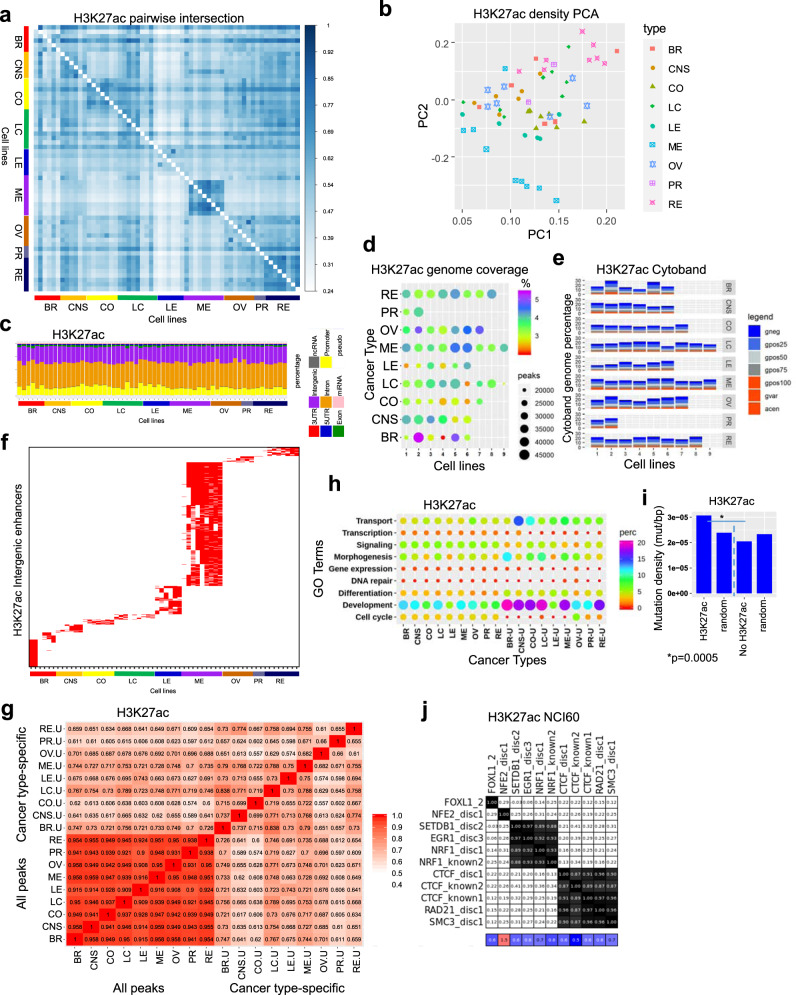
Typical H3K27ac enhancer profiling across multiple types of cancer. **a** Pairwise intersection of SICER-defined H3K27ac peaks (FDR < 0.0001) in 60 cancer cell lines. Heat map of pairwise intersection of H3K27ac regions was generated using Intervene. **b** PCA showing H3K27ac density (norm. tag density) across 60 cancer cell lines. **c** Annotation of genomic regions enriched with H3K27ac peaks in 60 cancer cell lines using HOMER. **d** Bubble plots showing H3K27ac genomic coverage for 60 cancer cells representing 9 types of cancers. Each row represents a cancer type. The size of the circle indicates the number of H3K27ac peaks and the color indicates the percentage of genome coverage. **e** Stacked barplot showing cytogenetic banding pattern of H3K27ac peaks. Cytobands were obtained from the UCSC genome browser. **f** Cancer type-specific H3K27ac-marked enhancer modules across 60 cell lines. H3K27ac-marked intergenic enhancers were diagonally sorted. **g** H3K27ac peaks nearby TSS of genes were functionally annotated using DAVID, and clustered using GoSemSim semantic similarity analysis. Biological process GO terms identified using DAVID. All H3K27ac peaks for 60 cell lines and cancer type-specific peaks were annotated. **h** Bubble plot showing enrichment of top biological process GO terms identified from all peaks and cancer type-specific peaks from 9 cancer types comprising 60 cell lines (u unique). **i** Mutation density (mutation/bp) in H3K27ac relative to random regions of similar size and frequency, and regions without H3K27ac. *p*-value was determined using a two-sided Fisher’s exact test. **j** Evaluation of enhancer regulatory motifs enriched in intergenic regions across 60 cancer cells. Encode motifs^64^ was used to perform motif analysis for intergenic H3K27ac ChIP-Seq datasets for 60 cells.

Enhancers are a non-coding DNA regulatory element typically bound by multiple transcription factors (TFs)^58,59^, which control cell type-specific gene regulatory profiles, and activity of enhancers is largely cell type-specific^60,61^. Enhancers play a critical role in cancer formation^62^, where enhancer activity is increased in cancer cells relative to normal tissue. While dynamic transcriptional networks and enhancer landscapes are often dysregulated in cancer cells^63^, it is unclear whether cancer type-specific enhancers or universal enhancers are activated in cancer cells. To interrogate dynamic enhancer activity at *cis*-regulatory elements across a panel of human cancer cells, we analyzed H3K27ac ChIP-Seq data generated in this study. Pairwise intersection analysis using Intervene revealed a high correlation of H3K27ac occupancy in multiple melanoma cell lines and renal cancer cells, and a correlation between CNS and colon cancer cells (Fig. 5a). This analysis also showed shared H3K27ac occupancy for several cancer types including renal, lung, and CNS cancers. We also observed overall differences in H3K27ac profiles between cells of different cancer types (inter-cancer), and cells within the same cancer type (intra-cancer), suggesting widespread heterogeneity of enhancer marking across multiple types of cancer. An evaluation of H3K27ac densities at ChIP-enriched peaks using PCA confirmed that melanoma, renal, colon, and CNS cancer cells clustered close to one another in the 2D space (Fig. 5b), while breast, lung, leukemia, ovarian, and prostate cancer cells clustered further away from one another, further suggesting greater heterogeneity in enhancer marking in those cancer cells. Functional annotation of regions enriched with H3K27ac using HOMER showed enrichment in predominantly intergenic and intron regions (Fig. 5c). An analysis of genome coverage revealed higher occupancy for several breast (HS-578T, MDA-MB-468), melanoma (LOX IMVI, SK-MEL-2, SK-MEL-28), and ovarian (NCI/ADR-RES, OVCAR-5, OVCAR-8, SK-OV-3) cancer cells, and lower occupancy for several breast (MDA-MB-231), colon (HCT-15, KM12), lung (NCI-H23), and leukemia (CCRF-CEM, MOLT-4) cells (Fig. 5d). The number of H3K27ac ChIP-enriched peaks is not always correlated with genome coverage, suggesting variability in the width of H3K27ac peaks. A comparison of H3K27ac peaks with cytogenetic banding patterns revealed that the majority of H3K27ac regions are located in relatively decondensed chromatin regions (Fig. 5e).

We further investigated enrichment of genomic mutations in regions with distinct cytogenetic banding patterns overlapping H3K27ac peaks. These findings reveal that substitution missense and coding silent mutations are enriched in chromatin regions containing G-negative light bands and G-positive bands intersecting H3K27ac peaks, while substitution nonsense mutations are enriched more in chromatin regions containing G-positive bands in several cancer cell lines (Supplementary Fig. 25). In addition, deletion frameshift mutations were enriched in chromatin regions containing G-positive bands. In contrast, insertion inframe, deletion inframe, and insertion frameshift mutations are enriched in chromatin regions containing G-negative and G-positive light bands. Moreover, we observed enrichment of complex insertion inframe mutations in G-positive light bands. Overall, these findings reveal variation in genomic location of mutation subtypes relative to cytogenetic banding patterns and H3K27ac peaks across 60 cancer cell genomes. These results also highlight heterogeneity in enrichment of mutations and chromosomal aberrations in H3K27ac regions with distinct cytogenetic banding patterns.

To identify cancer cell type-specific enhancer modules we focused on intergenic H3K27ac regions. Pairwise intersection analysis using Intervene and clustering analysis of all intergenic H3K27ac regions revealed cancer type-specific and intra-cancer heterogeneity of enhancer patterning (Supplementary Fig. 26a, b). A further comparison of public ChIP-Seq data from normal cells using Intervene pairwise intersection analysis revealed that intergenic H3K27ac patterning is mostly distinct between NCI-60 cancer cells and normal cells (Supplementary Fig. 27), with the exception of normal renal cells and astrocytes, which exhibited decreased overlap with other normal cells or cancer cells. Moreover, k-means clustering analysis of H3K27ac intergenic enhancers further revealed cancer type-specific and normal H3K27ac intergenic patterns (Supplementary Fig. 28a, b). Clusters which exhibit heterogeneity in H3K27ac patterning in cancer cells (clusters 3, 5, 6, 8, 10, 11, 13) exhibit decreased H3K27ac enrichment in normal cells (Supplementary Fig. 28b, c), while clusters with heterogeneous occupancy of intergenic H3K27ac in normal cells (clusters 2, 4, 7, 9, 12, 17) exhibit decreased occupancy in cancer cells. We also observed heterogeneous intergenic H3K27ac patterning across cancer and normal cells at a smaller number of enhancers (clusters 18–21). These findings also reveal that activity of a subset of enhancers is similar between normal cells and cancer cells. Enhancers in cluster 5 were activated in renal cancer cells and normal cells, and cluster 16 enhancers exhibited decreased activity in leukemia cells and normal blood cells.

Combined, these findings suggest that a subset of H3K27ac marked enhancers are distinct between cancer cells and normal cells, while a subset of enhancers are active in a cell type-specific manner in both cancer and normal cells. These results suggest that cancer type-specific heterogeneity in intergenic H3K27ac patterning may be due in part to a combination of differential enhancer patterning in the cell of origin and acquisition of differential enhancer patterns during cancer formation.

Next, we subsequently clustered, in ascending order, cancer type-specific regions that were found in at least 50% of cell lines for a cancer type (Fig. 5f). This strategy enabled us to distinguish intergenic enhancer modules specific to each cancer type, and to identify shared enhancer modules across cell lines from the same cancer type (intra-cancer). We next sought to identify patterns of functional annotation GO terms enriched within cancer type-specific modules. Following identification of enriched GO terms using NCBI DAVID, we performed semantic analysis using GoSemSim. These findings highlight overall similar enrichment of GO terms for all annotated intergenic H3K27ac peaks, but reveal distinct cancer type-specific enrichment of GO terms (Fig. 5g). Developmental and differentiation GO terms were overrepresented in breast and lung cancer, followed by CNS, colon, melanoma, and renal cancer cells (Fig. 5h). Ovarian, prostate, and leukemia cells exhibited lower enrichment of developmental and differentiation terms.

We also investigated whether genomic mutations are enriched in H3K27ac occupied regions or regions with H3K27ac. Our results demonstrate that H3K27ac occupied regions exhibit a higher density of mutations relative to random genomic regions and regions without H3K27ac (Fig. 5i). These finding are consistent to our results described for H3K4me3, and suggest that chromatin regions with active histone modifications have increased mutation rates relative to regions without active chromatin marks.

In addition, we performed a systematic search for composite regulatory motifs using ENCODE motifs^64^, and observed enrichment of several DNA sequence motifs for transcription factor binding within intergenic H3K27ac enhancer modules, including FOXL1, NFE2, SETDB1, EGR1, NRF1, CTCF, and the CTCF subunits RAD21, and SMC3 (Fig. 5j). While CTCF is a genomic insulator, which can block enhancer activity and prevent crosstalk between active and inactive chromatin regions^65^, CTCF has also been shown to mediate enhancer–promoter interactions^66^. These findings suggest that a subset of enhancer modules may be co-regulated across multiple types of cancers, which may be controlled by common upstream regulators. In addition, we investigated whether DNA sequence motifs for TF binding contain genetic mutations. While we observed mutations in DNA sequence motifs for binding of FOXL1, NFE2, SETDB1, EGR1, NRF1, CTCF, RAD21, and SMC3 on a genome-wide scale (Supplementary Fig. 29), we did not observe mutations in H3K27ac intergenic regions. We also found that the binding motif of CTCF exhibited an increased mutation frequency in the first two nucleotides (Supplementary Fig. 29). Genetic alteration of DNA-binding motifs may negatively impact TF binding and affect expression of associated genes.

### Super-enhancer (SE) activity is correlated with cancer type-specific genes

**Fig. 6:**
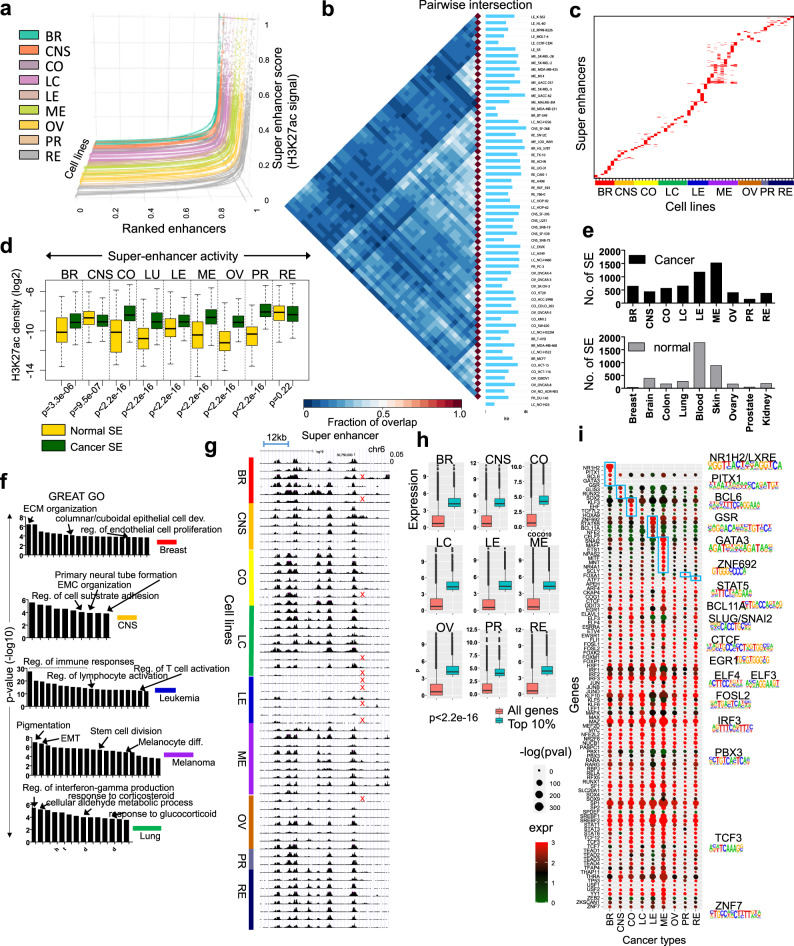
Identification of super enhancers (SE) in cancer epigenomes. **a** Super slope saturation curves of H3K27ac densities across 60 human cancer cell line datasets. The number of ranked typical and super enhancers (SE) marked by H3K27ac are plotted. H3K27ac normalized ChIP-Seq signal across a subset of all H3K27ac marked enhancers. SE were identified using HOMER (see the “Methods” section). SE are identified as regions that are located beyond where the slope is 1. **b** Intervene pairwise intersection of H3K27ac-defined SE. **c** Heat map showing diagonally sorted SE identified in 60 cancer cells. **d** Boxplot depicting SE activity at typical or normal enhancers (yellow) and SE (green) in cancer. H3K27ac densities (log2 norm. tag density) are shown. Boxplots indicate the 1st and 3rd quartiles (25th and 75th percentile, upper and lower bounds), 2nd quartile (center), and minima−maxima (1.5*interquartile range, whiskers). *p*-values were calculated using two-sided K–S tests. **e** Number of SE in cancer (black) and normal cells (gray). **f** *p*-values were calculated using GREAT^70^ GO functional annotation of cancer type-specific SE regions (−log10 *p*-value). **g** UCSC browser view of a SE cluster. Red ‘x’ indicates absence of a super enhancer. **h** Boxplot of RNA-Seq expression (log2 RPKM) of the top 10% expressed transcript encoding and all transcripts across 9 types of cancer in 60 cells. *p*-value for all <2.2e−16 (K–S test). Boxplots indicate the 1st and 3rd quartiles (25th and 75th percentile, upper and lower bounds), 2nd quartile (center), and minima−maxima (1.5*interquartile range, whiskers). **i** Heat map showing enrichment of transcription factor-binding sites (TFBS) in H3K27ac-defined SE regions across 9 cancer types. TFs expressed in the top 10% of all transcripts in at least one cancer type, and whose recognition motif was significantly enriched in SE regions (*p* < 0.05). The size of the circle is proportional to the *p*-value of the motif enrichment (−log10 (*p*-value), and the color of the circle is representative of the expression level of the TF in a given cancer type (red, high expression; green, low expression). Representative sequence logos of enriched motifs are shown. HOMER motif analysis^50^ was used to calculate *p*-values. Blue boxes show cancer-specific enrichment of TF-binding sites. Source data are provided as a Source Data file.

SEs are clusters of enhancers in close proximity that promote transcription of genes that define cell states^29,67^. In cancer, SEs are generated at oncogenes and at cancer promoting genes^67^, where cancer cells are addicted to aberrant enhancer activity^68^. To understand the relationship between SEs and cellular states that define cancer, we interrogated the super-enhancer repertoire across multiple types of cancer cells. Using ChIP-Seq data and HOMER^50^, we distinguished SE from typical enhancers (TE) based on H3K27ac signal, using a strategy based on Whyte et al. ^29^. H3K27ac peaks identified within a 12.5 kb region were stitched together and putative enhancers with the highest score were defined as SEs (see the “Methods” section)^50^. A subset of enhancers exhibit a high level of H3K27ac occupancy (Fig. 6a). In contrast to TE (Supplementary Fig. 26b, Fig. 5f), the majority of SEs are cell type-specific (Fig. 6b, c). We observed decreased overlap in H3K27ac occupancy across cell types for SEs relative to normal enhancers (Fig. 5a). In addition, we compared activity of SEs identified from normal cells to cancer-cell specific SEs identified in this study. A comprehensive list of SEs from nine normal tissues was extracted from the human super-enhancer database (SEdb)^69^. Results from this analysis demonstrate that breast, colon, lung, leukemia, melanoma, ovarian, and prostate cancer SEs exhibit increased activity relative to normal tissue SEs (Fig. 6d), while CNS and renal exhibited a slight decrease in activity. We also observed a greater number of cancer SEs relative to normal tissue SEs (Fig. 6e). Functional characterization of genes associated with SEs using GREAT^70^ gene ontology analysis revealed that genes are linked to biological processes related to their respective cancer type (Fig. 6f; Supplementary Data 5; *p*-values were calculated using GREAT^70^). A representative view of a super-enhancer cluster, identified in a subset of NCI-60 cell lines (~80% of cell lines), shows dynamic H3K27ac patterning (Fig. 6g). Using a list of super enhancer regions obtained from SEdb^69^, we found that most normal cells do not exhibit super enhancer patterning in this genomic region. A further comparison of public ChIP-Seq from normal cells revealed decreased H3K27ac levels at this representative genomic region relative to cancer cells (Supplementary Fig. 30a, b). Combined, these findings demonstrate that super enhancers are cell type-specific in cancer cells, and distinct between normal and cancer cells.

To investigate transcription factor-binding site enrichment across multiple types of cancers, we analyzed frequencies of cognate consensus DNA-binding motifs at super-enhancer regions using HOMER motif analysis^50^. Next, we evaluated the average expression of TFs whose recognition motifs were statistically significant (consensus binding motifs) for each cancer type, and subsequently focused on the highest expressing TFs (top 10%) for each cancer type (Fig. 6h). Using this approach, we identified 103 transcription factor recognition motifs for nine types of cancer cells (Fig. 6i). Known oncogenes such as MYC were expressed at a high level across nine types of cancer cells. We also identified TFs that were expressed at a high level in a cancer type specific manner, and whose motifs were statistically enriched. We identified breast cancer enriched TFs including GATA3, PITX2, NR1H2, BCL6, and GSR, CNS-enriched TFs RUNX2, SOX2, and GLIS3, colon cancer-enriched TFs TCF7L2, EHF, HOXA9, and KLF3, leukemia-enriched TFs ZNF692, STAT5B, CELF2, BCL11A, and NFE2, melanoma-enriched TFs including MITF, SNAI2, MAFF, NR4A1, NPAS2, MNT, ETS1, and SCLY, the prostate cancer-enriched TF, FOXA1, and the renal cancer-enriched TF, ATF7 (Fig. 6i). Overall, these findings reveal key insight into cancer type-specific regulation of SEs, and further suggest that common upstream TFs may regulate distinct target genes across various cancer types.

### The heterochromatin landscape of cancer cells

The chromatin landscape is demarcated into two classes: euchromatin, which is open and transcriptionally active, and heterochromatin, which is compact and generally transcriptionally silent^30^. Heterochromatin exists as two states: constitutive heterochromatin, which is stably heterochromatinized in a condensed state and facultative heterochromatin, which is dynamically repressed during development. Cancer formation involves the dysregulation of constitutive and facultative heterochromatin states. H3K9me3 and H4K20me3 co-localize at constitutive and facultative heterochromatin enriched with repetitive DNA elements^71–74^, and these histone modifications serve as proxies for repressed chromatin regions. H3K9me3 domains mark constitutive and tissue-specific regions, which are refractory to binding by transcriptional regulators^75^.

H3K9me3 and H4K20me3 may function as redundant histone modifications that promote heterochromatin formation^76^, as the H3K9 histone methyltransferases (HMTases) SUV39H1 and SUV39H2 have been shown to act upstream of the H4K20 HMTases SUV420H1 and SUV420H2^33,77^. Dysregulated expression or mutation of H3K9me3/H4K20me3 histone methyltransferases (HMTases) in cancer cells may lead to perturbed sequential deposition of H3K9me3 and H4K20me3, or altered patterns of H3K9me3 and H4K20me3 deposition, thus leading to heterochromatin dysregulation and genome instability.

**Fig. 7:**
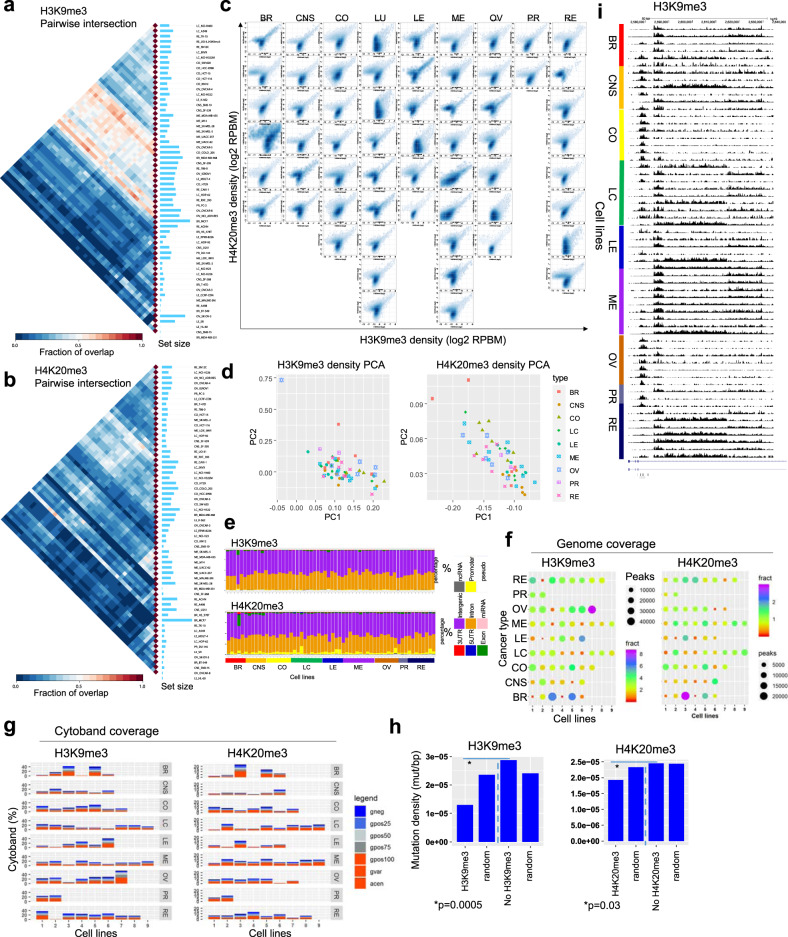
Heterochromatin dynamics in cancer epigenomes. **a** and **b** Pairwise intersection of SICER-defined (FDR < 0.0001) **a** H3K9me3 and **b** H4K20me3 enriched regions in 60 cancer cells. Heat map of pairwise intersection was generated using Intervene. **c** Scatter plots of H3K9me3 and H4K20me3 densities (log2 norm. tag density) across 60 cancer cell lines representing 9 cancer subtypes. **d** PCA analysis of H3K4me9 (left) and H4K20me3 (right) densities (norm. tag density) in 60 cell lines. Cancer types are color coded. **e** Genomic positional annotation of regions enriched with H3K9me3 (top) and H4K20me3 (bottom) in 60 cancer cell lines using HOMER. **f** Bubble plots showing H3K9me3 (left) and H4K20me3 (right) genomic coverage for 60 cancer cells representing 9 types of cancers. Each row represents a cancer type. The size of the circle indicates the number of H3K9me3 or H4K20me3 peaks and the color indicates the percentage of genome coverage. **g** Stacked barplot showing cytogenetic banding pattern of H3K9me3 (left) and H4K20me3 (right) peaks. **h** Mutation density (mutation/bp) in H3K9me3 (left) and H4K20me3 (right) regions relative to random regions of similar size and frequency, and regions without H3K9me3 or H4K20me3. *p*-value was determined using a two-sided Fisher’s exact test. **i** UCSC browser view of H3K9me3-marked domains in 60 cells. Source data are provided as a Source Data file.

To understand dynamic regulation of repressed chromatin in cancer cells, we interrogated the heterochromatin landscape by surveying H3K9me3 and H4K20me3 occupancy across a spectrum of cancer types. An evaluation of pairwise intersections between H3K9me3-enriched regions revealed largely distinct repressive chromatin landscapes between 60 cancer cells, suggesting heterogeneous marking of heterochromatin regions (Fig. 7a). In addition, we observed a decreased overlap of H4K20me3 peaks (Fig. 7b) relative to H3K9me3, H3K27ac, or H3K4me3 peaks, suggesting loss of H4K20me3 across multiple types of cancers. We also compared H3K9me3 and H4K20me3 densities across 60 cancer cells, and observed co-enrichment across the heterochromatin landscape (Fig. 7c). Dual marking of H3K9me3 and H4K20me3 may reflect a central heterochromatin feature. By interrogating H3K9me3 and H4K20me3 densities using PCA, we observed heterogeneous repressive landscapes between cells from different types of cancers (Fig. 7d). Annotation of genomic regions occupied by H3K9me3 or H4K20me3 revealed enrichment in intergenic and intronic regions (Fig. 7e). Moreover, we observed a greater percentage of MDA-MB-231 H4K20me3 peaks located in exon regions relative to other NCI-60 cells, and a greater percentage of SK-OV-3 H4K20me3 peaks located in promoter regions relative to most NCI-60 cell lines. Next, we performed functional annotation of genes nearby H3K9me3 or H4K20me3 domains using NCBI DAVID. Using this approach, we found heterogeneous developmental GO term enrichment of H3K9me3-associated genes (Supplementary Fig. 31a, c), and even greater diversity of enrichment of developmental GO terms for genes associated with H4K20me3 (Supplementary Fig. 31b, d). These results suggest that developmental gene expression programs may exhibit aberrant marking or loss of H3K9me3 or H4K20me3 in cancer cells. Genome coverage analyses showed several cell lines with higher coverage of H3K9me3 domains across the genome (ovarian: SK-OV-3; leukemia: SR; breast: MCF7, MDA-MB-468) (Fig. 7f). Moreover, MCF7 breast cancer cells exhibited the greatest coverage of H4K20me3 across 60 cancer cells. While cytogenetic banding patterns of genomic regions occupied by H3K9me3 or H4K20me3 revealed enrichment in condensed chromatin regions (Fig. 7g), a subset of regions were enriched in open chromatin regions.

We also investigated a correlation between regions enriched with H3K9me3 or H4K20me3 and the presence of genomic variations. These findings show that H3K9me3 and H4K20me3 marked regions have a lower density of mutations relative to random genomic fragments or regions without H3K9me3 or H4K20me3 (Fig. 7h). However, these mutations do not include structural translocation events, which may occur as a result of loss of H4K20me3. These findings suggest that compact heterochromatinized genomic regions are more refractory to genomic mutations relative to open euchromatin regions (Fig. 3h). Overall, these results describe dynamic regulation of heterochromatin regions across multiple types of cancer cells.

## Discussion

Here, we interrogated the epigenetic landscape of multiple types of cancer cells from the NCI-60 panel. We describe results from an integrative analysis of histone modification profiling and epigenetic and genomic features to gain insight into dynamic regulation of active and inactivate chromatin states across a diverse set of 60 cancer cell lines. We observed high variability in histone modifications associated with enhancer (H3K27ac) or heterochromatin (H3K9me3, H4K20me3) regions relative to promoter regions (H3K4me3). Activity of regulatory regions is related to their chromatin context. Variability in H3K27ac distributions between cancer cells may reflect dysregulation of enhancer landscapes through re-purposing of enhancers to drive oncogene expression^67,78^. The ability of cancer cells to exhibit distorted enhancer regulatory profiles of driver oncogenes may allow cancer cells to adapt to a new environment or evade anti-proliferative therapies.

Findings described in this study also provide a resource to interrogate associations between histone modification patterning and gene activity at the genic or genome-wide level. An investigation of associations between H3K4me3, H3K27ac, H3K9me3, and H4K20me3 densities and gene activity demonstrated that while H3K4me3 and H3K27ac marks are both positively correlated with gene activity, H3K4me3 densities are more strongly correlated with the level of gene expression relative to intergenic H3K27ac (Supplementary Figs. 32 and 33). In contrast, H3K9me3 and H4K20me3 densities are negatively correlated with gene expression level (Supplementary Figs. 34 and 35).

Our analyses also provide insight into relationships between cancer type-specific chromatin states. We observed combinations of histone modifications that reflect various bivalent chromatin states, including bivalent enhancers and promoters, marked by activating histone modifications H3K4me3 or H3K27ac, and repressive histone modifications H3K9me3 or H4K20me3. While previous work has revealed the co-occurrence of H3K4me3 and H3K27me3^79^, and H3K4me3 and H4K20me3^74^, it is unclear whether cancer cells exhibit altered patterning of bivalently marked chromatin, or whether cancer cells establish de novo bivalently marked chromatin regions. The presence of bivalently marked chromatin regions may serve to poise chromatin regions for activation upon differentiation, or repression during differentiation, which was suggested previously^74^. Several studies have suggested the existence of H3K4me3/H3K27me3 bivalent domains in cancer cells^80^, although H3K4me3/H4K20me3 have not been previously described for cancer cells. Moreover, H3K27ac/H3K9me3 bivalent enhancers have not been previously described. Aberrant dual marking of chromatin regions may lead to altered gene activity of oncogenes and tumor suppressors.

We also uncovered coordinated activity of enhancer modules across several types of cancers, while SEs were largely cell type-specific. Genes associated with SEs were also expressed in a cancer-cell-specific manner, and linked to biological processes distinct to cancer types. We identified potential upstream regulators of highly expressed TFs whose binding sites are enriched across multiple types of cancers, and cancer type-specific regulatory modules (Fig. 6i). These results highlight known oncogenic TFs such as MYC, and associated protein MAX^81^, whose expression is correlated with decreased survival, and additional TFs which may represent novel cancer disposition genes: we identified other TFs whose expression and recognition motifs were enriched in super-enhancer regions, and whose expression is correlated with decreased survival. These results provide a resource to further interrogate cancer cell identity and therapeutic targeting of cancer type and cell type-specific genes and pathways. Along this line, targeting of SEs has been shown to be a therapeutic strategy to mitigate expression of oncogenes and related downstream pathways^82^.

Our results also reveal that DNA sequence variation in cancer cells is associated with active chromatin regions, which is consistent with previous findings, which showed that genomic variation is enriched in transcriptional regulatory regions with accessible chromatin^83^. While previous findings observed enrichment of SNPs in regulatory DNA, our findings demonstrate that diverse types of mutations (deletions, insertions, substitution, complex) are enriched in active chromatin regions relative to H3K9me3 and H4K20me3 marked regions. We found that mutations were overrepresented in H3K4me3 occupied regions, which are proxies for active promoter regions, and H3K27ac marked regions, which are proxies for typical and super-enhancer regions. Because promoters and enhancers regulate expression of genes that define cell identity, mutations in regulatory regions which results in dysregulated expression may contribute to cancer formation and tumor progression. These findings suggest that an evaluation of the mutational profile of non-coding transcriptional regulatory regions may provide additional insight into aberrant gene expression programs in cancer cells, which may not be detectable using exome-sequencing.

While cancer cells can be distinguished by their overall promoter and enhancer profiling, our results suggest that heterochromatin patterning is more dynamic between cancer cells, suggesting aberrant patterning of heterochromatin is largely cell-specific, rather than cancer type-specific. Intriguingly, we observed heterogeneous enrichment of developmental and differentiation biological process GO terms across cells from nine types of cancers. Dysregulated heterochromatin formation or instability may lead to cancer susceptibility or tumor progression by aberrant repression of tumor suppressor genes^84^, where stochastic repression of tumor suppressors may facilitate cancer formation. Aberrant repression of genes may occur following DNA damage repair, where repressive histone modifications are deposited at the break site, and if they are not removed may lead to sustained silencing^85^.

Aberrant DNA methylation as an epigenetic mechanism for gene silencing has been extensively studied in the context of cancer^86^, and evidence suggests that the repressive histone modifications H3K9me3^87^ and H4K20me3^88^ can also be dysregulated in cancer cells. Targeting the heterochromatin landscape may represent a therapeutic strategy to reactivate repressed genes, as the general methylation inhibitor DZNep was capable of removing H3K27me3^89^. Moreover, depletion of SUV39H1 resulted in reactivation of silenced genes^87^.

Alterations in patterning of repressive histone modifications such as H3K9me3 or H4K20me3 may lead to instability of the underlying repetitive DNA sequences. Decreased enrichment of H3K9me3 or H4K20me3 in cancer cells may result in a more relaxed chromatin state with altered local chromatin topologies, including changes in chromatin loop dynamics^90^, which may affect translocations between neighboring chromosomes^91^. Dysregulation of chromatin loop configurations may result in proximal double-strand breaks, illegitimate joining, and translocations. DNA polymerase slippage may also occur during replication of repetitive DNA regions due to chromatin decondensation in cancers cells with altered H3K9me3 and H4K20me3 levels and distributions. Alternatively, polymerase slippage during DNA replication may lead to an expansion of repetitive DNA regions and subsequent translocation events or chromosomal aberrations during differentiation.

Our results reveal enrichment of repetitive DNA elements in chromatin states with depleted levels of H4K20me3 at heterochromatin regions marked by H3K9me3 (E11) or depleted levels of H3K9me3 at regions marked by H4K20me3 (E1). LINE and LTR repeats are enriched in chromatin states E1 and E11, while RNA, satellite, rRNA, and tRNA repeats are enriched in chromatin state E12 (H3K9me3/H3K4me3), and several repeats such as DNA, SINE, and to a lesser extent LINE and LTR repeats are enriched in chromatin state E8 (H4K20me3/H3K27ac). In addition, we observed cancer type-specific variability in enrichment of mutations in chromatin states marked by H4K20me3 (E1) or H3K9me3 (E11) (Supplementary Fig. 5C, Fig. 1B). H3K9me3-marked regions (E11) exhibit increased enrichment of mutations relative to H4K20me3 regions (E1) in melanoma and CNS tumor cells, while H4K20me3 regions (E1) exhibit increased enrichment of mutations relative to H3K9me3 regions (E11) in colon, leukemia, lung, ovarian, prostate, and kidney cancer cells. Moreover, we also observed variable enrichment of mutations between H3K9me3/H3K4me3 (E12) and H4K20me3/H3K27ac (E8) regions, where H3K9me3/H3K4me3 (E12) regions exhibited increased enrichment of mutations relative to H4K20me3/H3K27ac (E8) regions. These results link histone modification profiles with DNA repeats and genetic mutations in a cancer type-specific manner, which provide insight into the relationship between alterations in H3K9me3 and H4K20me3 heterochromatin patterning and the dysregulated cancer epigenome. Moreover, stochastic chromosomal aberrations resulting from changes in heterochromatin patterning may drive tumorigenesis or tumor progression.

Genetic variation of genes encoding H3K9 or H4K20 methyltransferases may lead to dysregulated patterns of H3K9me3 and H4K20me3 deposition, which may contribute to variability in enrichment of mutations in chromatin states marked by H4K20me3 or H3K9me3, and in chromatin regions enriched with repetitive DNA elements. Pathogenic missense mutations (substitution) in EHMT1, which deposits H3K9me1/2, were observed in DU-145 (c.3744G>T), HCT-15 (c.1919C>T), NCI-H23 (c.3595C>T), and SF539 (c.1A>G) cells, while pathogenic missense mutations (substitution) in EHMT2, which deposits H3K9me2, were found in LOX IMVI (c.124G>A), M14 (c.2980 T > G), and MOLT-4 (c.2995G>A¸ c.1679C>A, c.2056C>T¸ c.1067 C > T) cells (Supplementary Data 2). SETDB1, which methylates H3K9 up to trimethylation (H3K9me3), is mutated in COLO-205 (substitution missense; c.796 G > A), IGROV1 (substitution missense; c.3709 G > A), and OVCAR-5 (substitution missense; c.2963 A > G) cells, while SETDB2, which also deposits H3K9me3, is mutated in KM12 cells (substitution missense; c.1621A > G). SUV39H2, which deposits H3K9me3, is mutated in DU-145 (substitution missense; c.953 G > T¸ c.1133 G > T), HCC2998 (substitution missense; c.2 T > G¸ c.182 T > G), and MOLT-4 cells (substitution missense; c.43 C > T). SUV420H2, which deposits H4K20me3, is mutated in HCT-15 (substitution missense; c.56 C > A) cells. SETD8, which deposits H4K20me1, is mutated in MOLT-4 cells (substitution missense; c.524 C > T), and mutations in SUV420H1, which deposits H4K20me1/2 were found in HCC2998 (substitution missense; c.1450 C > A), KM12 (substitution nonsense; c.2095 C > T), and SK-MEL-2 (deletion frameshift; c.843delT) cells. In addition, HCC2998 cells exhibit a substitution nonsense mutation in HP1 (c.463 G > T), which is critical for heterochromatin formation.

We also observed variable RNA-Seq expression^7^ of repetitive DNA elements across 60 cancer cell lines. While expression of rRNA or RNA repeats was higher than other DNA repeat class members across 60 cancer cells (Supplementary Fig. 36a), several cancer cell lines with a lower number of H4K20me3 and H3K9me3 peaks (MDA-MB-231, SW-620, NCI-H226, NCI-H522, HL-60, OVCAR-5) (Fig. 1a) exhibited relatively higher expression of several repetitive DNA class members (Supplementary Fig. 36a). In addition, we observed variable cancer type-specific expression of repetitive DNA elements in chromatin states with depleted H4K20me3 (E11) or H3K9me3 (E1), or bivalently marked chromatin states E12 (H3K9me3/H3K4me3) or E8 (H4K20me3/H3K27ac) (Supplementary Fig. 36b).

Cancer cell heterogeneity can occur between tumors originating from the same cell type or tissue type (inter-tumor heterogeneity) or within individual tumors (intra-tumor heterogeneity)^92^. Understanding whether cell-of-origin heterogeneity or acquired heterogeneity during carcinogenesis explains cell type or cancer type-specific differences in epigenomic patterning is a fundamental question in cancer biology. Cancer type and cell type-specific heterogeneity may also be explained in part through cancer cell plasticity^93^, where cancer cells dedifferentiate or undergo reprogramming^94^ towards an alternate cellular fate. Heterogeneity may also arise due to epigenetic plasticity^95^, where cancer cells sample distinct chromatin states, some of which allow cancer cells to adapt to environments and evade therapies. In addition, the cancer stem cell (CSC) model, which posits that cancer cells are organized in a hierarchy of CSCs, differentiated cancer cells, and non-tumorigenic progeny, also provides a possible explanation for cancer cell heterogeneity. Reversible or irreversible alterations in cancer cell phenotypes and epigenomic profiles may occur in a cancer type or cell type-specific manner.

Overall, results from this study provide a catalog of the dynamic patterning of activating (H3K4me3 and H3K27ac) and repressive histone modifications (H3K9me3 and H4K20me3) across 60 cancer cells. These findings also serve as a resource for modeling active and inactive chromatin domains in the NCI-60 panel of cancer cells.

## Methods

### NCI-60 cell culture

The NCI-60 cell lines were obtained from the NCI DTP Tumor Repository. The NCI DTP Tumor Repository performed Applied Biosystems AmpFLSTR Identifiler testing with PCR amplification to confirm consistency with the published Identifiler STR profile for each of the NCI-60 cell lines (Supplementary Data 6). Cells were cultured in RPMI 1640/5% FBS media containing glutamine and pen/strep at 37 °C with 5% CO_2_.

### ChIP-Seq analysis

ChIP-Seq experiments were performed as previously described with minor modifications^96,97^. The rabbit monoclonal antibody H3K4me3 (17-614) antibody was obtained from Millipore, and the rabbit polyclonal H3K27ac (ab4729), rabbit polyclonal H3K9me3 (ab8898), and rabbit polyclonal H4K20me3 (ab9053) antibodies were obtained from Abcam. In brief, 15 million human cancer cells were harvested by trypsinizing into a single-cell suspension and crosslinked with formaldehyde (1%) for 10 min. at 37 °C. Fixed cell pellets were subsequently flash frozen and stored at −80 °C. Next, cell pellets were thawed and subsequently sonicated, and cell extracts equivalent to 5 million cells were used for ChIP assays using 4 µL antibody. ChIP-enriched DNA was end-repaired using the End-It DNA End-Repair kit (Epicentre), followed by addition of a single A nucleotide, and ligation of Illumina adapters. PCR was performed using Phusion 2× high fidelity PCR master mix. ChIP libraries were sequenced on an Illumina HiSeq platforms according to the manufacture’s protocol. Sequence reads were mapped to the human genome (hg19) using bowtie2^98^ with default settings. C++ programs to convert a SAM formatted file to a BED6 format from bowtie2 (Sam2Bed6_Bowtie2), to remove redundant reads from a BED6 file (RemoveRedundantReads), and to convert a BED6 file to a BEDGraph file (GenerateRPBMBasedSummary) were described previously^99^.

ChIP-Seq read-enriched regions (peaks) were identified relative to control Input DNA using “Spatial Clustering for Identification of ChIP-Enriched Regions” (SICER) software^100^ with a window size setting of 200 bps, a gap setting of 400 bps, a FDR setting of 0.001. The SICER-compare function was used to compare multiple samples (FDR < 0.001, fold-change > 1.5). ChIP-Seq libraries were normalized by library size: the RPBM measure (read per base per million reads) was used to quantify densities at genomic regions from ChIP-Seq datasets. Two biological replicates were performed for the ChIP-Seq analyses. The Kolmogorov–Smirnov test was used to obtain *p*-value statistics for comparing density of ChIP-enrichment at genomic regions. The UCSC genome browser was used to visualize normalized ChIP data.

### Broad H3K4me3 domains

We included a stringent definition of a broad H3K4me3 peak. H3K4me3 peaks that intersected TSS regions of hg19 refseq genes, and whose length exceeds 4 kb in length (≥ 4 kb) were considered broad H3K4me3 domains, while H3K4me3 peaks whose length was less than 4 kb (<4 kb) were considered sharp peaks.

### RNA-Seq analysis

Normalized hg19 RNA-Seq data for NCI60 cells was downloaded from Cellminer^7^. The FPKM measure (fragments per kilobases of exon model per million reads) was used to quantify the mRNA expression level of a gene from RNA-Seq data.

### Chromatin state learning

We identified chromatin states across 60 NCI-60 epigenomes using ChromHMM v1.2^101^ software, which utilizes a multivariate HMM. The ChromHMM model was learned by concatenating histone modification data (ChIP-enriched peaks, see ChIP-Seq methods above) for H3K4me3, H3K27ac, H3K9me3, and H4K20me3. For each ChIP-Seq dataset, peaks were evaluated in 200 bp bin intervals across the genome. Bins were binarized to two states, 1 indicating peak enrichment and 0 indicating no enrichment. The 15-state model was used because it identified major combinatorial patterns of histone modifications across the genome.

### Chromatin state annotations

The 15 chromatin states were annotated using CpG islands downloaded from the UCSC genome browser website. Genic features such as TSSs, TES, genes, exons, introns, were integrated into ChromHMM using data from http://hgdownload.cse.ucsc.edu/goldenPath/hg19/encodeDCC/wgEncodeGencodeV10/, as described previously^44^. Genes were categorized into either expressed or non-expressed transcripts by their RNA-Seq expression level in H1 human pluripotent stem cells (hPS) cells. Zinc finger genes were obtained from ENSEMBL annotation by filtering genes whose name start with ZNF. Transcription factor-binding site (TFBS) data was obtained from analyzed ENCODE ChIP-Seq data (http://hgdownload.cse.ucsc.edu/goldenPath/hg19/encodeDCC/wgEncodeAwgTfbsUniform/). Genomic evolutionary rate profiling (GERP) elements from 34 way placental alignment were obtained from: http://mendel.stanford.edu/SidowLab/downloads/gerp/. Genome coverage was evaluated using bedtools coverage.

### Variation in DNA methylation across chromatin states

DNA methylation (percentage of CpG methlation from whole genome bisulfite sequencing—WGBS data)^9^ was evaluated for regions associated with 15 chromatin states (Fig. 2c). The average methylation level (0–100%) of CpGs was computed for each region across all epigenomes. The average percent CpG methylation was plotted using the R package ggplot2.

### Relationship between histone modifications and DNA methylation

We evaluated DNA methylation level at tumor suppressors and oncogenes at regions occupied with or without H3K4me3 peaks. WGBS data^9^ was used to evaluate methylation level (0–1.0) of CpGs for each region across all epigenomes. The R package ggplot2 was used to plot a heatmap of DNA methylation for 9 subtypes of cancer.

### Lamina-associated domains

Lamina-associated domains (Fig. 1b) obtained from human lamin B1 in SHEF-2 ES cells (GEO: GSE22428) were downloaded from the ChromHMM (https://github.com/jernst98/ChromHMM/blob/master/COORDS/hg19/laminB1lads.hg19.bed.gz).

### Chromatin state switching

We calculated chromatin switching using data from the 15 chromatin states for any two states across 60 NCI60 epigenomes (Fig. 2a) in a similar manner as previously described^44^. For example, for a pair of states 1 and 2, we computed the number of genomic bins that contained (1,2) or (2,1) chromatin states in all pairs. Chromatin state switching frequencies were converted to switching probabilities by normalizing. Use of switching probabilities avoids the dominance of states with high frequencies by limiting the dependence on the number of epigenomes.

To investigate dynamic state switching in cell lines from the same cancer type (intra-cancer), we computed chromatin state frequencies for cell lines from the same cancer type. The frequencies were then averaged across all cancer sub-types and subsequently row normalized to generate switching probabilities. Figure 2a (left) shows intra-cancer type chromatin switching probabilities. We performed an analogous analysis to evaluate differences in chromatin switching between cancer sub-types (inter-cancer), by calculating inter-cancer switching frequencies and switching probabilities. Results of inter-cancer switching probabilities are shown in Fig. 2a (right).

### Conservation score calculation

The R libraries GenomicScores, regioneR, phastCons100way.UCSC.hg19, and GenomicRanges were used to calculate conservation scores. PhastCons conservation scores (phastCons100way.UCSC.hg19) were obtained from the UCSC Genome Browser, which were calculated from multiple genome alignments (human genome hg19–99 vertebrate species).

### Global chromatin structure

To evaluate chromatin structure on a global scale we computed frequencies of 15 states identified in 200 bp bins from ChromHMM across 60 NCI60 epigenomes. Next, we averaged frequencies in 2 Mb genomic bins across the genome, divided the observed frequency by the random frequency and calculated the normalized averaged frequency, and subsequently performed hierarchical clustering. Results shown in Fig. 1d reveal clusters of regions enriched with various chromatin states. These clusters are differentially enriched with gene density and lamin-B1, as shown in Fig. 1d. Figure 1d also shows cytogenetic banding patterns across the 2 Mb bins, which were downloaded from the UCSC genome browser.

### Mutation analysis

Whole-exome sequencing data for NCI-60 cancer cells^8^ was downloaded from Cosmic^47^. An evaluation of mutations in regions enriched with histone modifications was performed using bedtools intersect. To compare enrichment of mutations across 60 cancer genomes, hierarchical clustering was performed as shown in Fig. 3f. Annotation of mutations (e.g. deleterious or silent) and their frequencies across 60 cancer genomes is shown in Fig. 3g. Mutation density was calculated in SICER-defined regions enriched with histone modifications (H3K4me3, H3K27ac, H3K9me3, and H4K20me3), and control regions (random genomic fragments of equivalent size and frequency as histone modification peaks, regions without histone modification peak, and random regions without histone modification peak). Results from these analyses are shown in Figs. 3h, 5i, 7h.

### Cancer type-specific regulators

To identify cancer type and cell type-specific transcriptional regulators in 60 cancer cell lines, we first filtered intergenic H3K27ac ChIP-enriched peaks for each cell line that were enriched in the majority of cell lines for a given cancer sub-type (peak present in at least 50% of cell lines for a sub-type of cancer). Next, k-means clustering of cancer type-specific enhancer modules were plotted in a heatmap in ascending order as shown in Fig. 5f. To identify transcriptional regulators across 60 NCI60 epigenomes, we performed motif discovery using ENCODE motifs^64^ and known motifs. Results from this analysis are shown in Fig. 5j.

### Super enhancer analysis

H3K27ac super enhancers were identified using HOMER^50^. In brief, all enhancers were ranked using the findPeaks function of HOMER, and all enhancers were plotted by exporting addition of the “-superSlope -1000” option. H3K27ac peaks that were found within 12.5 kb of one another were stitched together. The signal of each super enhancer region is calculated by the number of normalized reads minus the number of normalized input reads. Regions are then sorted, normalized the highest score, and the number of typical enhancer regions. Super enhancers are defined as regions past the point where the slope is 1 (slope > 1). Results from this analysis are shown in Fig. 6a.

We then identified cancer-subtype and cell type-specific super enhancers, and plotted enhancer modules in ascending order as shown in Fig. 6c. Next, we annotated nearby genes and evaluated their expression using RNA-Seq data^7^, and filtered the top 10% expressed TFs for each cancer type. We then performed motif discovery using HOMER motif analysis^50^. Transcription factor genes whose expression is in the top 10%, and whose consensus binding motif is enriched in super enhancer regions in a cancer type-specific manner (*p* < 0.05), were plotted using ggplot2 in Fig. 6i.

### Gene ontology functional annotation

DAVID^102^ was used to functionally annotate genes, and subsequently evaluated by semantic analysis using GoSemSim software^51^ (*p* < 0.05 was considered significant). Enrichment of tumor suppressors, oncogenes, and housekeeping genes was evaluated using Fisher’s exact tests.

### Statistics and reproducibility

We generated biological duplicate H3K4me3, H3K27ac, H3K9me3, and H4K20me3 ChIP-Seq datasets for the NCI-60 panel.

### Reporting summary

Further information on research design is available in the Nature Research Reporting Summary linked to this article.

## Supplementary information

Supplementary Information

Description of Additional Supplementary Files

Supplementary Data 1

Supplementary Data 2

Supplementary Data 3

Supplementary Data 4

Supplementary Data 5

Supplementary Data 6

Supplementary Data 7

Reporting Summary

## Data Availability

The sequencing data from this study have been submitted to the NCBI Gene Expression Omnibus (GEO) under accession no. GSE143653 [https://www.ncbi.nlm.nih.gov/geo/query/acc.cgi?acc=GSE143653]. A description of the ChIP-Seq samples can be found in Supplementary Data 7. Publicly available H3K27ac ChIP-Seq datasets analyzed in this study: GSM1003459, GSM1027287, GSM1003559, GSM1102782, GSM2152595, GSM772859, GSM999004, GSM1102781, GSM1847878, GSM999000, GSM999001, GSM2741449, GSM773004, GSM1027288, GSM2527658, GSM1633870, GSM733763, GSM2698422, GSM2293347, GSM906395, GSM1013123, GSM956009, GSM4250668, GSM2699699, GSM910559, GSM1666386, GSM1662338, GSM2698631 (Supplementary Fig. 27). Publicly available H3K4me3 ChIP-Seq datasets analyzed in this study: GSM1427065, GSM1647618, GSM1666384, GSM1782766, GSM1874929, GSM2035818, GSM2067930, GSM2736544, GSM3011841, GSM3011844, GSM3011847, GSM3011850, GSM4315283, GSM529959, GSM529964, GSM529966, GSM529967, GSM621457, GSM621665, GSM733720, GSM733747, GSM773041, GSM883691, GSM883692, GSM945276, GSM947523, GSM971341, SRR11600891, SRR11600898 (Supplementary Fig. 14). All data are available from the authors upon reasonable request. Source data are provided with this paper.

## Source data

Source Data
