# Supplementary material for: Integrative pan cancer analysis reveals epigenomic variation in cancer type and cell specific chromatin domains
Source: Nat Commun. 2021 Mar 3;12:1419. doi: 10.1038/s41467-021-21707-1 (PMC7930052; doi:10.1038/s41467-021-21707-1)
Supplement: Supplementary file 1 — Supplementary Information [file 41467_2021_21707_MOESM1_ESM.pdf]

SUPPLEMENTARY INFORMATION

Integrative pan cancer analysis reveals epigenomic variation in cancer type and cell specific chromatin domains

Lijin K. Gopi[1-2], Benjamin L. Kidder[1-2*]

[1]Department of Oncology, Wayne State University School of Medicine, Detroit, MI, USA

[2]Karmanos Cancer Institute, Wayne State University School of Medicine, Detroit, MI, USA

[*]Correspondence:

Benjamin L. Kidder

Email: benjamin.kidder@wayne.edu

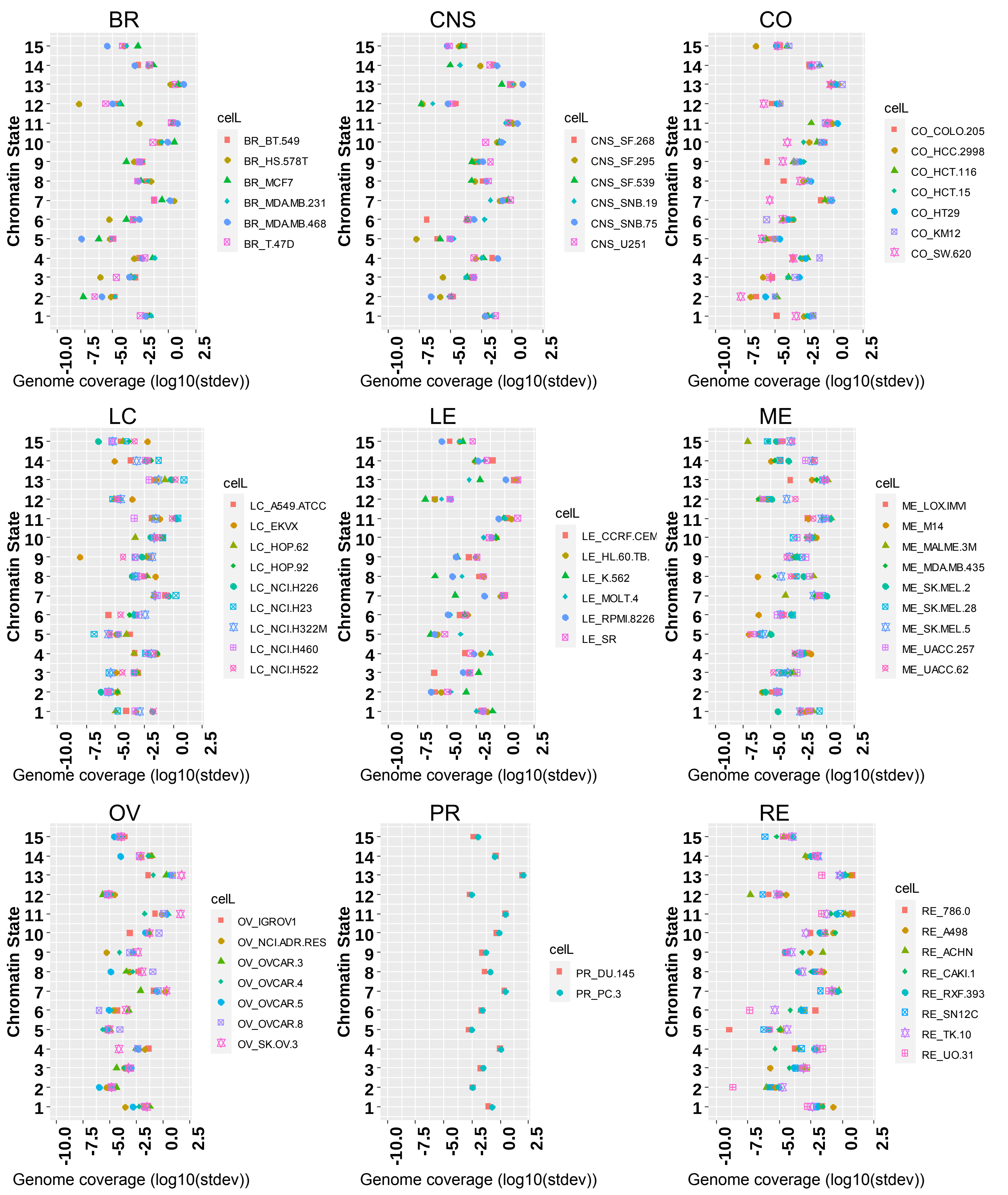

Supplementary Fig. 1

Supplementary Fig. 1. Genome coverage of 15 chromatin states. Relative chromatin state frequency for 60 cancer cells representing 9 types of cancers. Genome coverage (log10 standard deviation) of each chromatin state for each cancer cell epigenome is shown. Source data are provided as a Source Data file.

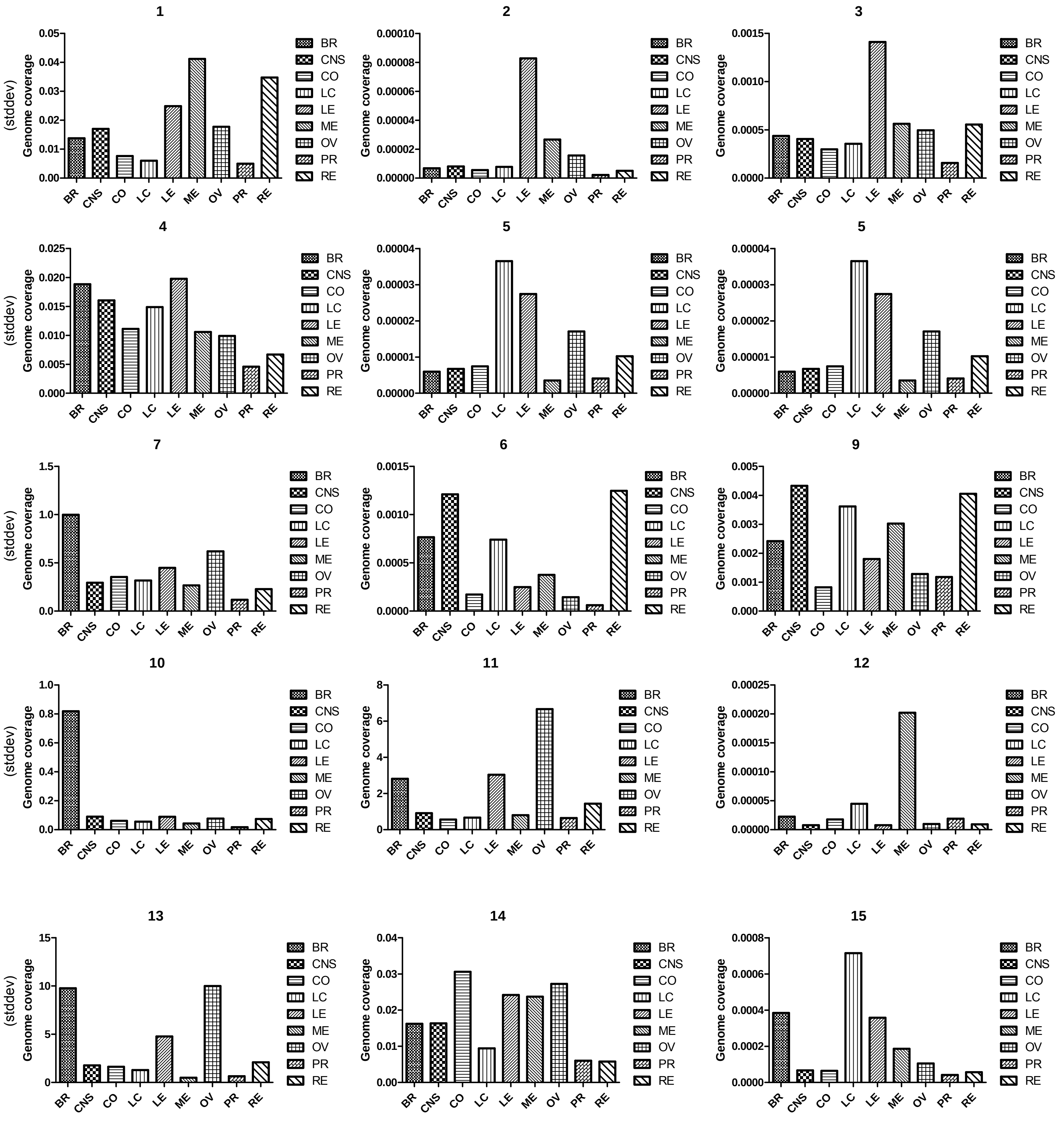

Supplementary Fig. 2

Supplementary Fig. 2. Genome coverage of 15 chromatin states. Relative chromatin state frequency for 9 types of cancers. Average genome coverage (stdev) of each chromatin state for each cancer type is shown. Source data are provided as a Source Data file.

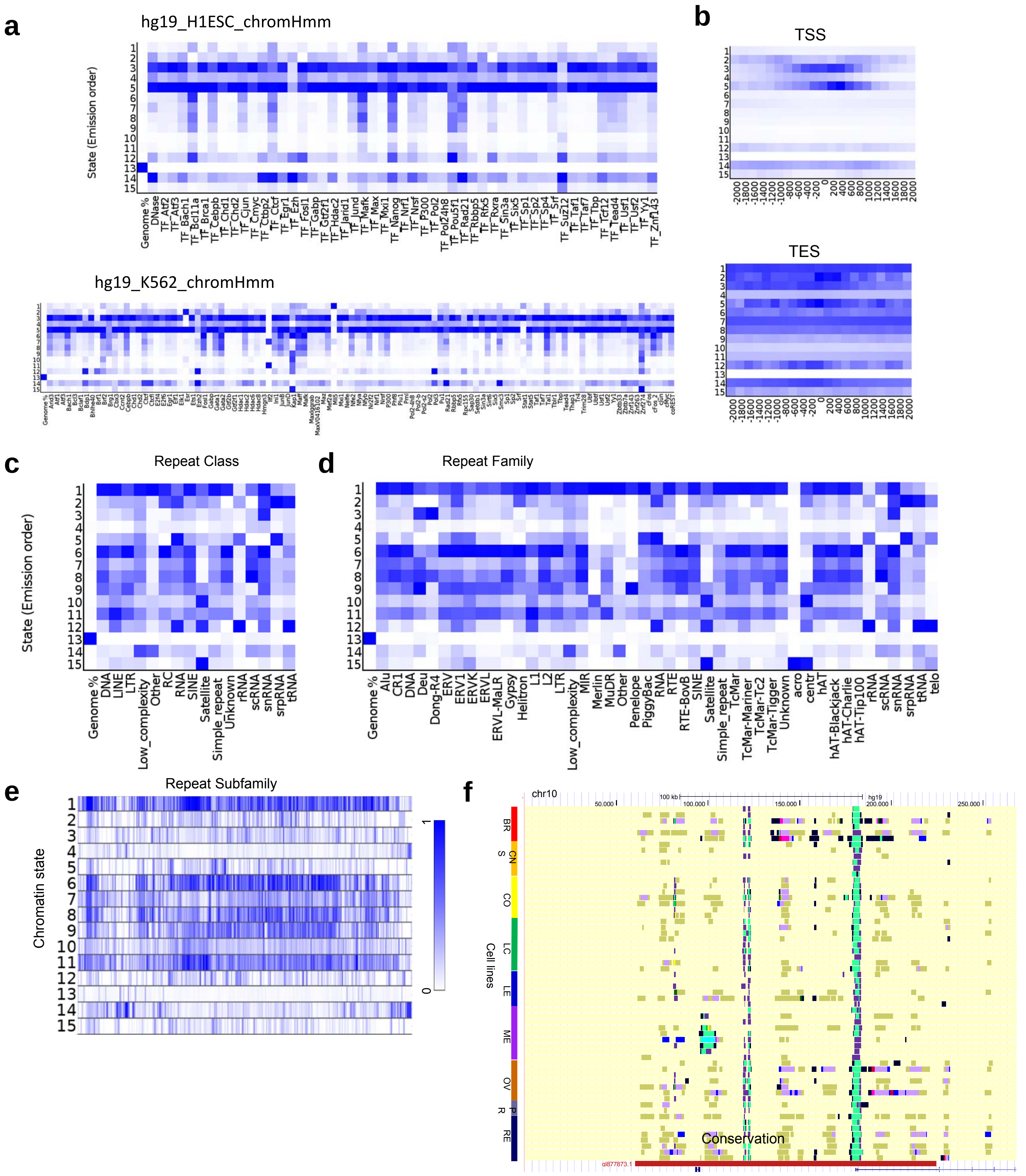

Supplementary Fig.3

Supplementary Fig. 3. Enrichment of repetitive DNA elements in chromatin states. (**a**) Chromatin state (15-state) enrichments generated from four histone modification profiles using 60 human cancer cells. Enrichment of transcription factor (TF) occupancy generated from ChIP-Seq data using H1 hES cells (top) and K-562 cells (bottom) in 15 chromatin states. Genome coverage and DNase hypersensitivity for H1 ES cells (top) and K-562 cells (bottom) is also depicted for the 15-state model. (**b**) ChromHMM neighborhood enrichment analysis: enrichment of 15 chromatin states at TSS (top) and TES (bottom) anchor positions. (**c-e**) Genome coverage and enrichment of repetitive DNA (**c**) class, (**d**) family, and (**e**) and subfamily members in the 15-state model. (**f**) UCSC browser view of ChromHMM annotations using the 15-state model, which was defined using 60 human cancer cell lines and profiling of four histone modifications. Each row represents one cell and cancer type. Source data are provided as a Source Data file.

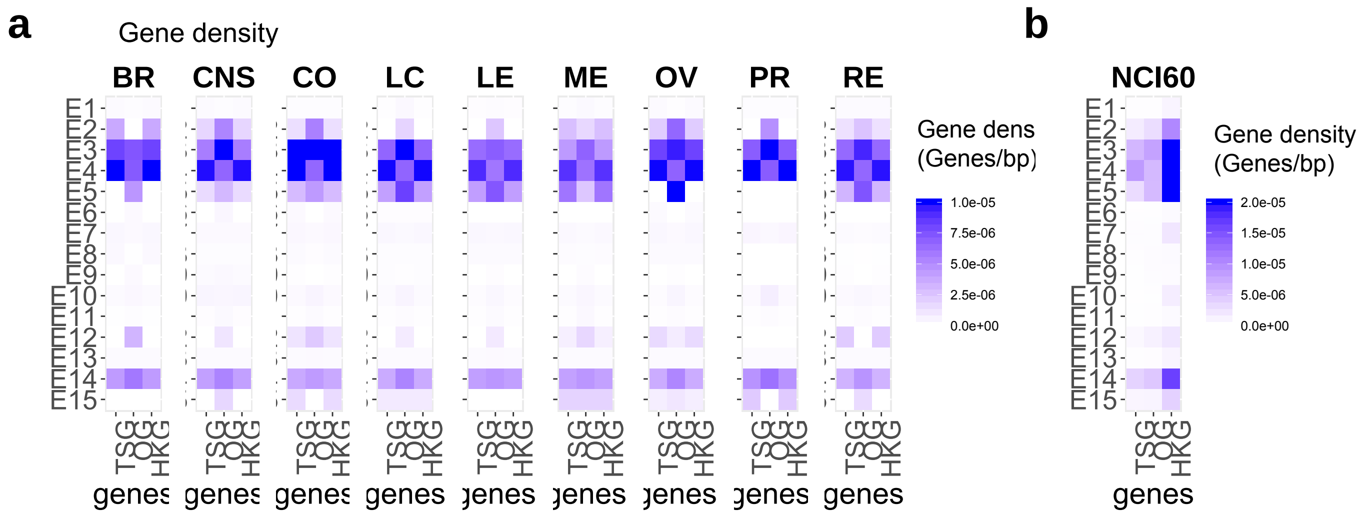

Supplementary Fig. 4

Supplementary Fig. 4. Enrichment analysis of tumor suppressors, oncogenes, and housekeeping genes in chromatin states. Analysis of tumor suppressor gene (TSG), oncogene (ONCG), and housekeeping gene (HKG) density (genes/bp) in 15 chromatin states for (**a**) 9 types of cancer and (**b**) 60 cancer cell lines. Source data are provided as a Source Data file.

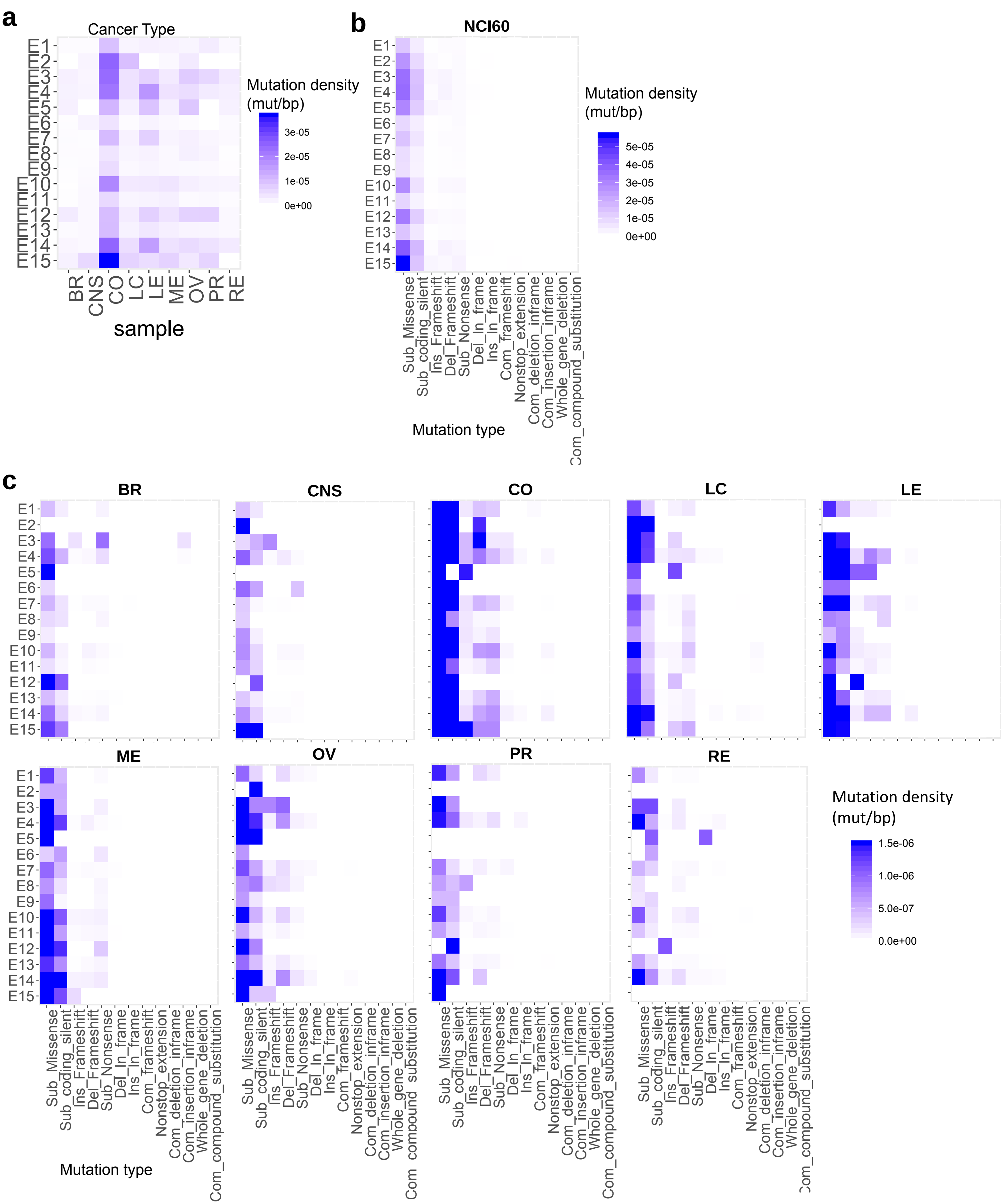

Supplementary Fig.5

Supplementary Fig. 5. Chromatin state cancer-type mutation analysis. (**a**) Cosmic mutation density analysis across 9 cancer types. Mutation density (mutation/bp). (**b**) Mutation type analysis across 60 cancer cell lines. Mutation density (mutation/bp). (**c**) Cancer-type mutation type density analysis. Mutation density (mutation/bp). Source data are provided as a Source Data file.

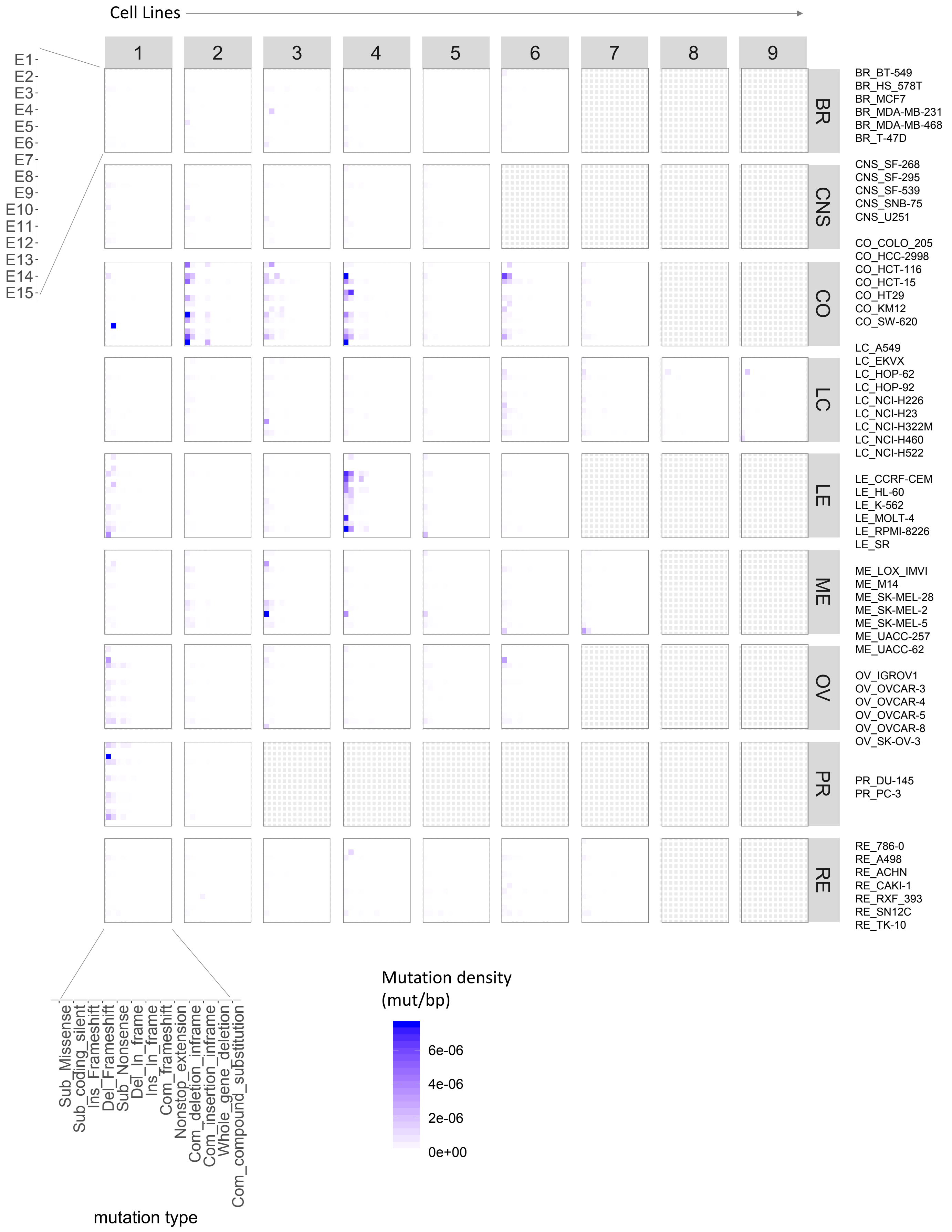

Supplementary Fig.6

Supplementary Fig. 6. Chromatin state cancer cell line mutation analysis. Cosmic mutation type analysis across 60 cancer cell lines. Mutation density (mutation/bp). Source data are provided as a Source Data file.

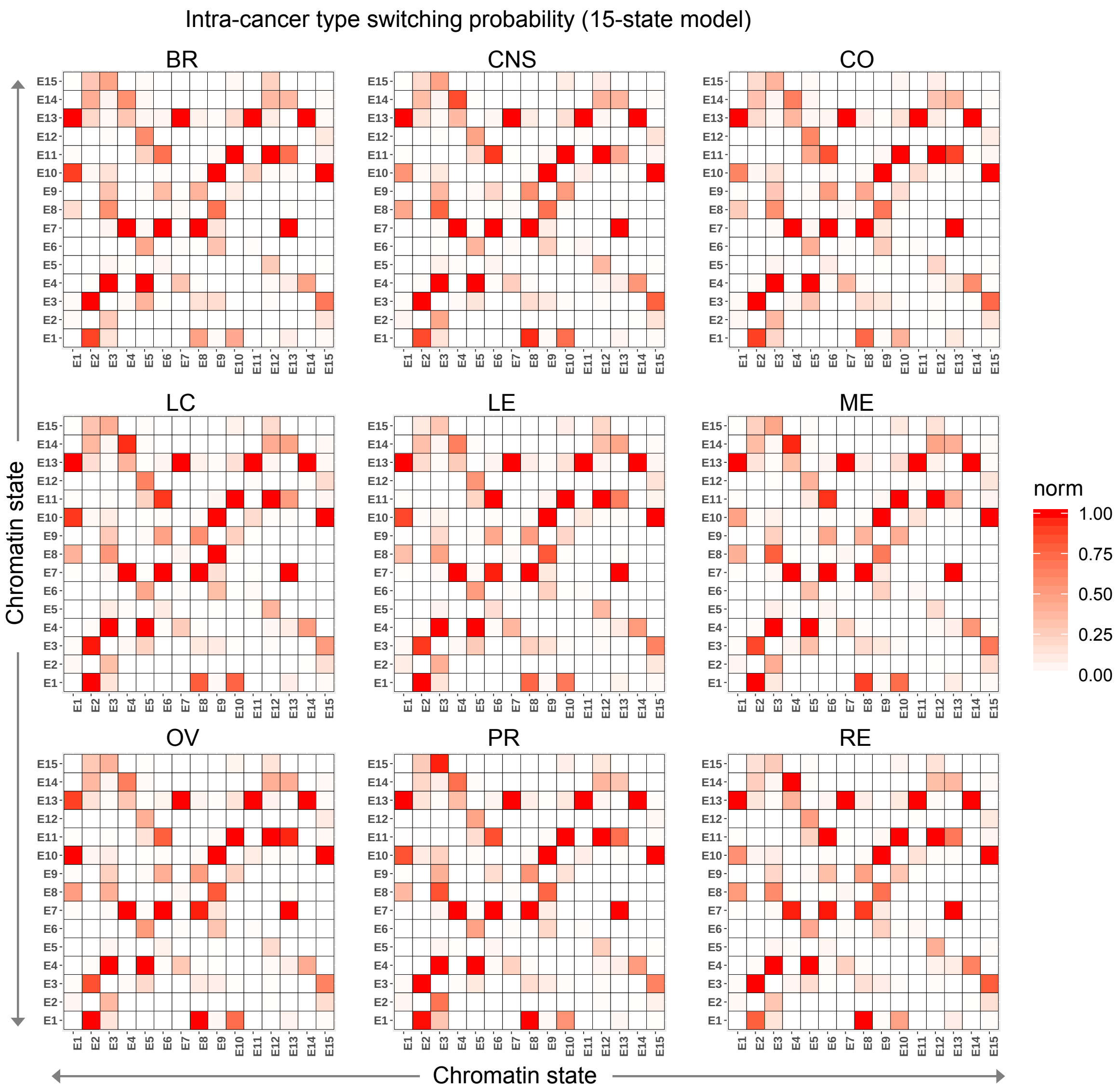

Supplementary Fig.7

Supplementary Fig. 7. Chromatin state switching dynamics in cancer cells. Intra-cancer type switching probabilities for 15 chromatin states across 9 cancer types comprised of 60 human cancer epigenomes. State transition (x-axis to y-axis). Source data are provided as a Source Data file.

Mutation density - Intra-cancer type state switching (15-state model)

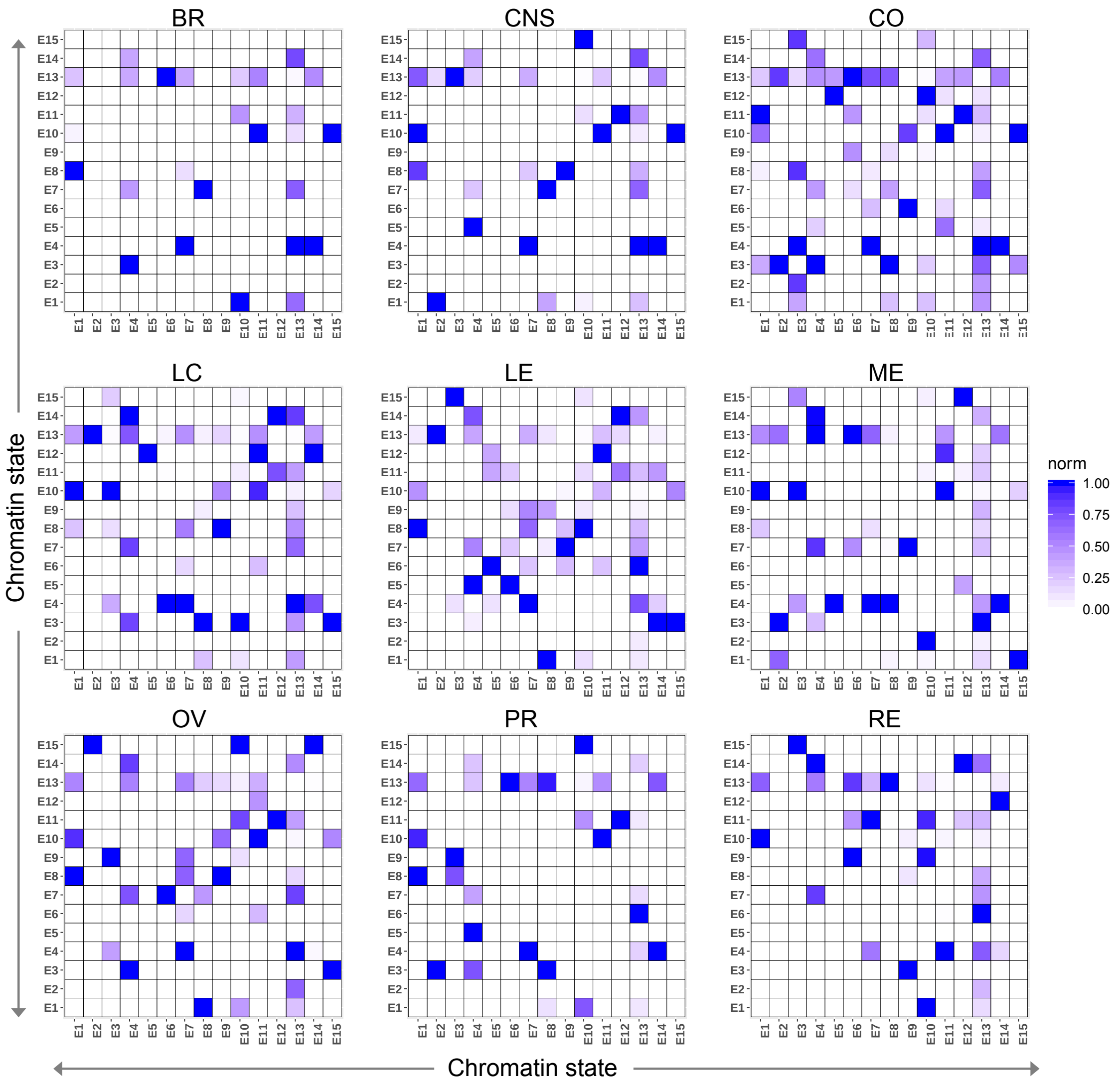

Supplementary Fig.8

Supplementary Fig. 8. Intra-cancer chromatin state switching mutation analysis. Cosmic mutation density analysis (column normalized 0 to 1) of intra-cancer type chromatin switching across 9 cancer types comprised of 60 human cancer epigenomes. State transition (x-axis to y-axis). Source data are provided as a Source Data file.

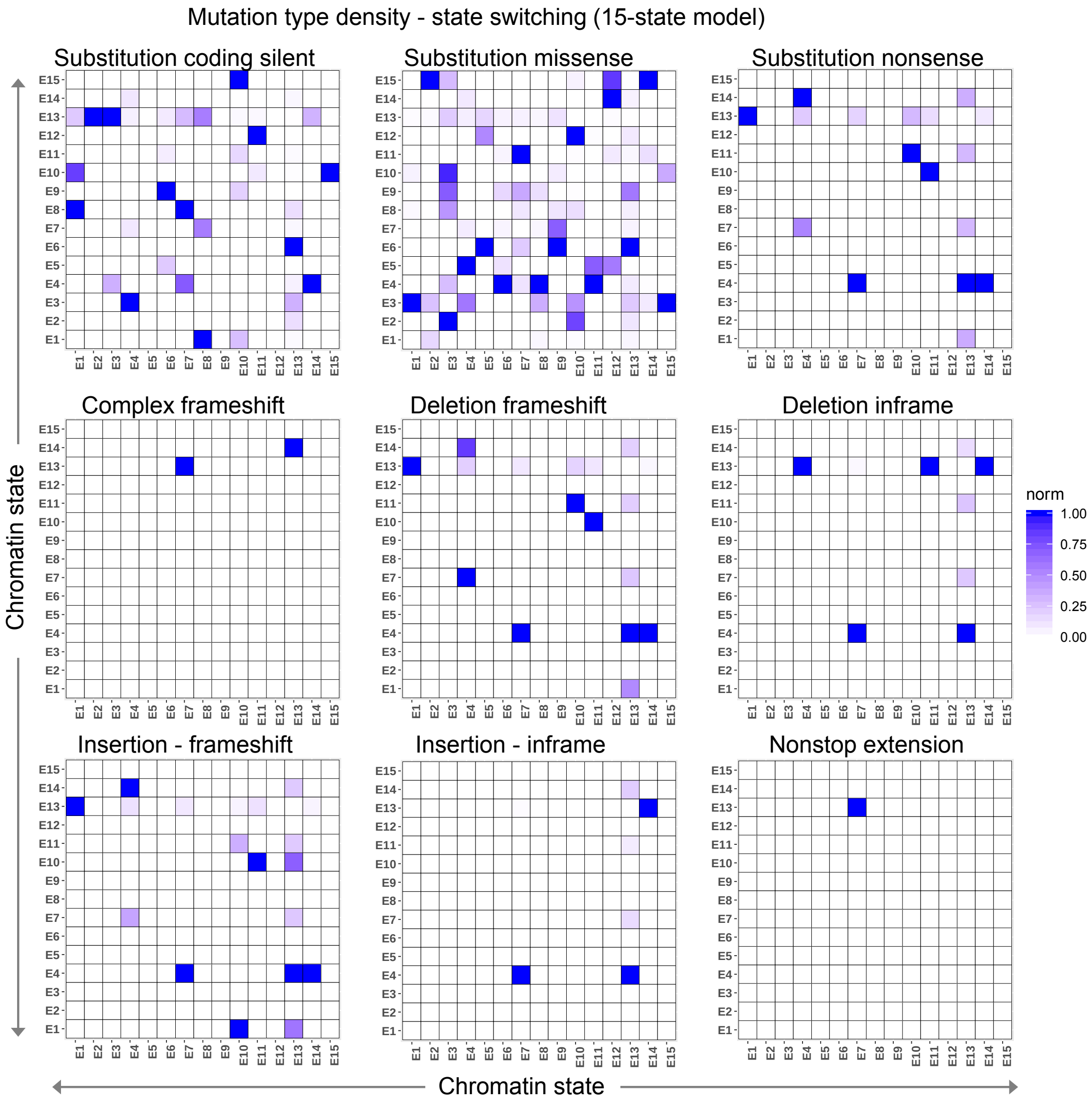

Supplementary Fig. 9

Supplementary Fig. 9. Chromatin state switching mutation type analysis. Mutation type density analysis (column normalized 0 to 1) of chromatin switching. Source data are provided as a Source Data file.

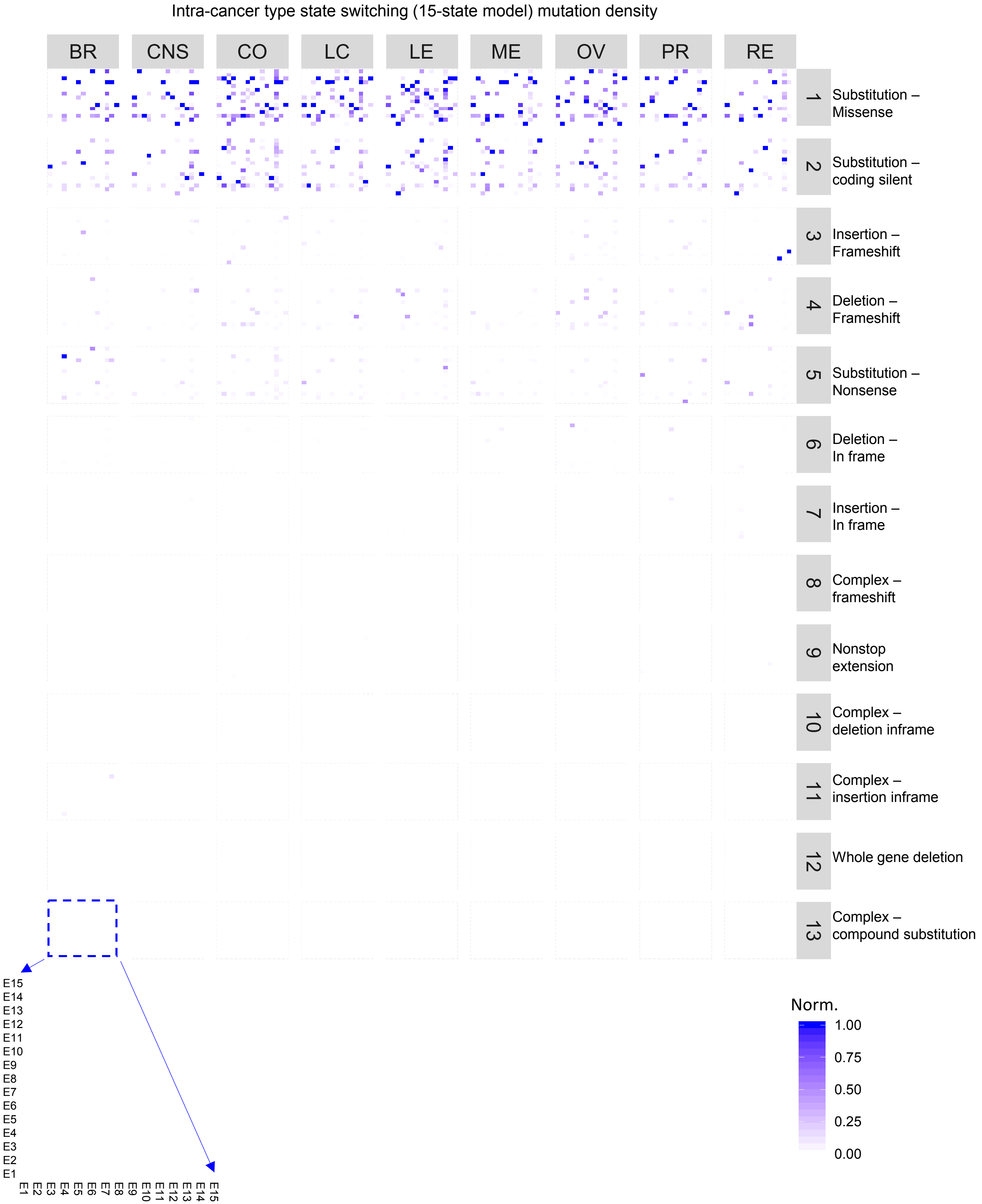

Supplementary Fig. 10

Supplementary Fig. 10. Cancer cell type chromatin state switching mutation analysis. Mutation type normalized density analysis (column normalized 0 to 1) of intra-cancer type chromatin switching across 60 human cancer epigenomes.

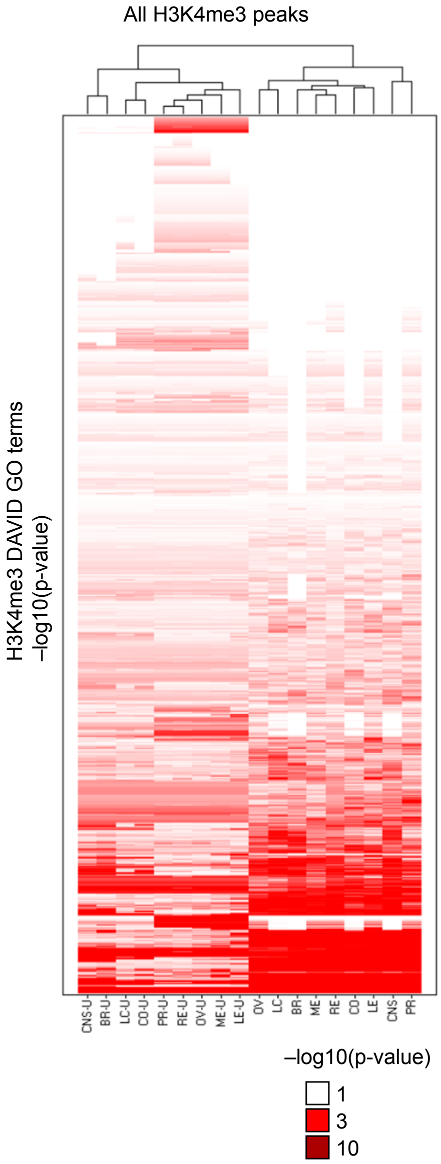

Supplementary Fig. 11

Supplementary Fig. 11. DAVID GO functional annotation of genes associated with H3K4me3. Hierarchical clustering heat map of enrichment of DAVID biological process GO terms (-log10 p-value) identified from genes associated with all H3K4me3 peaks and cancer type-specific H3K4me3 peaks from 9 cancer types comprising 60 cell lines (u: unique). NCBI DAVID was used to calculate p-values. Source data are provided as a Source Data file.

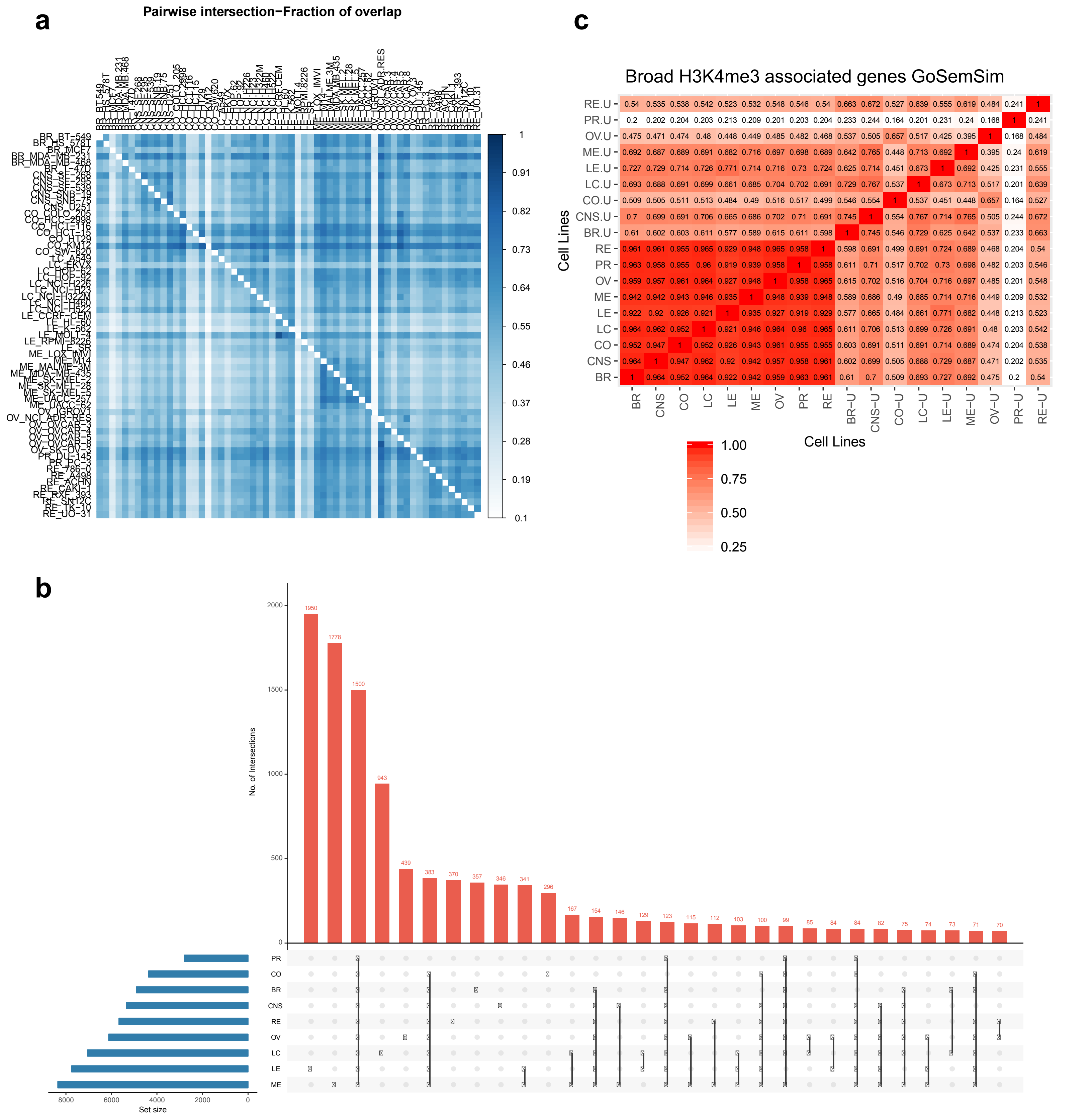

Supplementary Fig. 12

Supplementary Fig. 12. Broad H3K4me3 domain analysis of human cancer cells. (a) Pairwise intersection of broad H3K4me3 peaks (>4 kb) in 60 cancer cell lines. Heat map of pairwise intersection using Jaccard statistics of broad H3K4me3 regions was generated using Intervene. (**b**) Intervene UpSet plot of intersection of broad H3K4me3 peaks for 9 types of cancer. (**c**) Heatmap of semantic similarity matrix depicting enrichment of top biological process GO terms identified from all broad H3K4me3 peaks and cancer-type specific broad H3K4me3 peaks from 9 cancer types comprising 60 cell lines (u: unique). Source data are provided as a Source Data file.

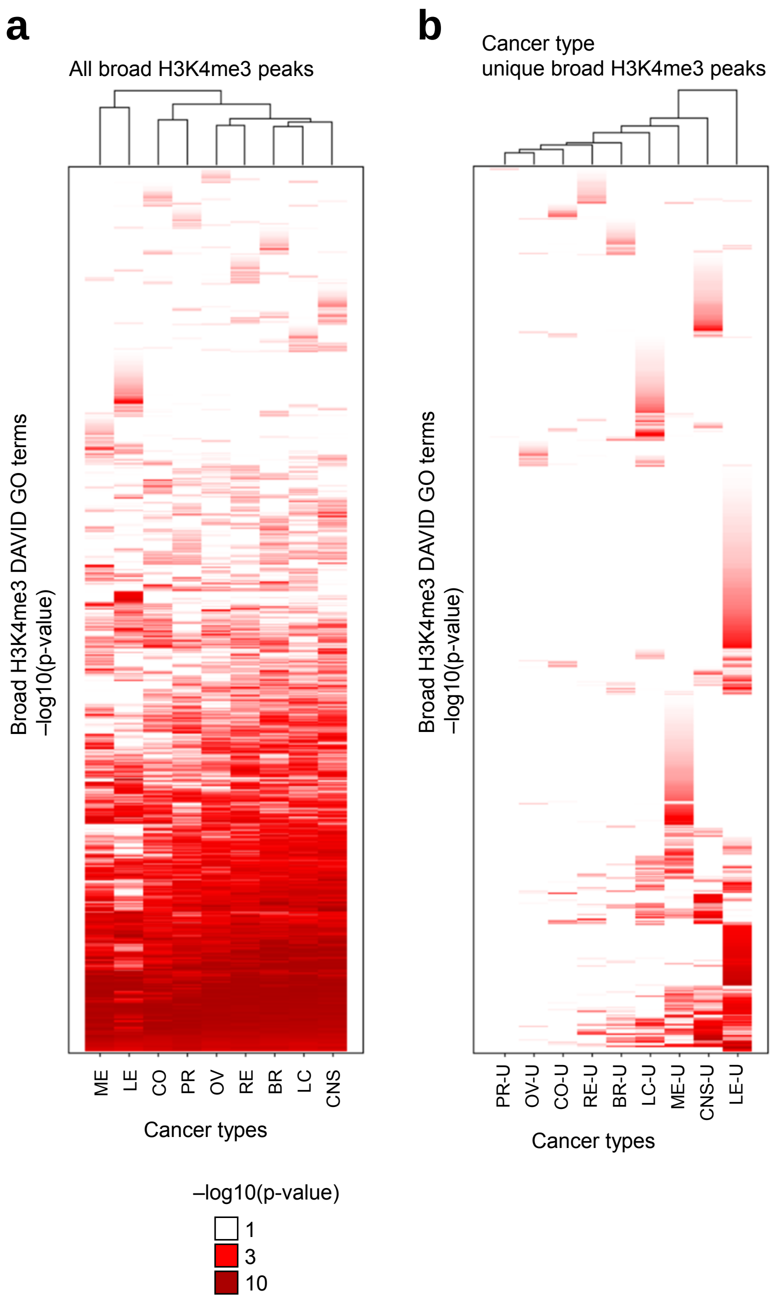

Supplementary Fig. 13

Supplementary Fig. 13. DAVID GO functional annotation of genes associated with broad H3K4me3. Hierarchical clustering heat map of enrichment of DAVID biological process GO terms (-log10 p-value) identified from genes associated with (**a**) all broad H3K4me3 peaks and (**b**) cancer-type specific H3K4me3 peaks from 9 cancer types comprising 60 cell lines (u: unique). NCBI DAVID was used to calculate p-values. Source data are provided as a Source Data file.

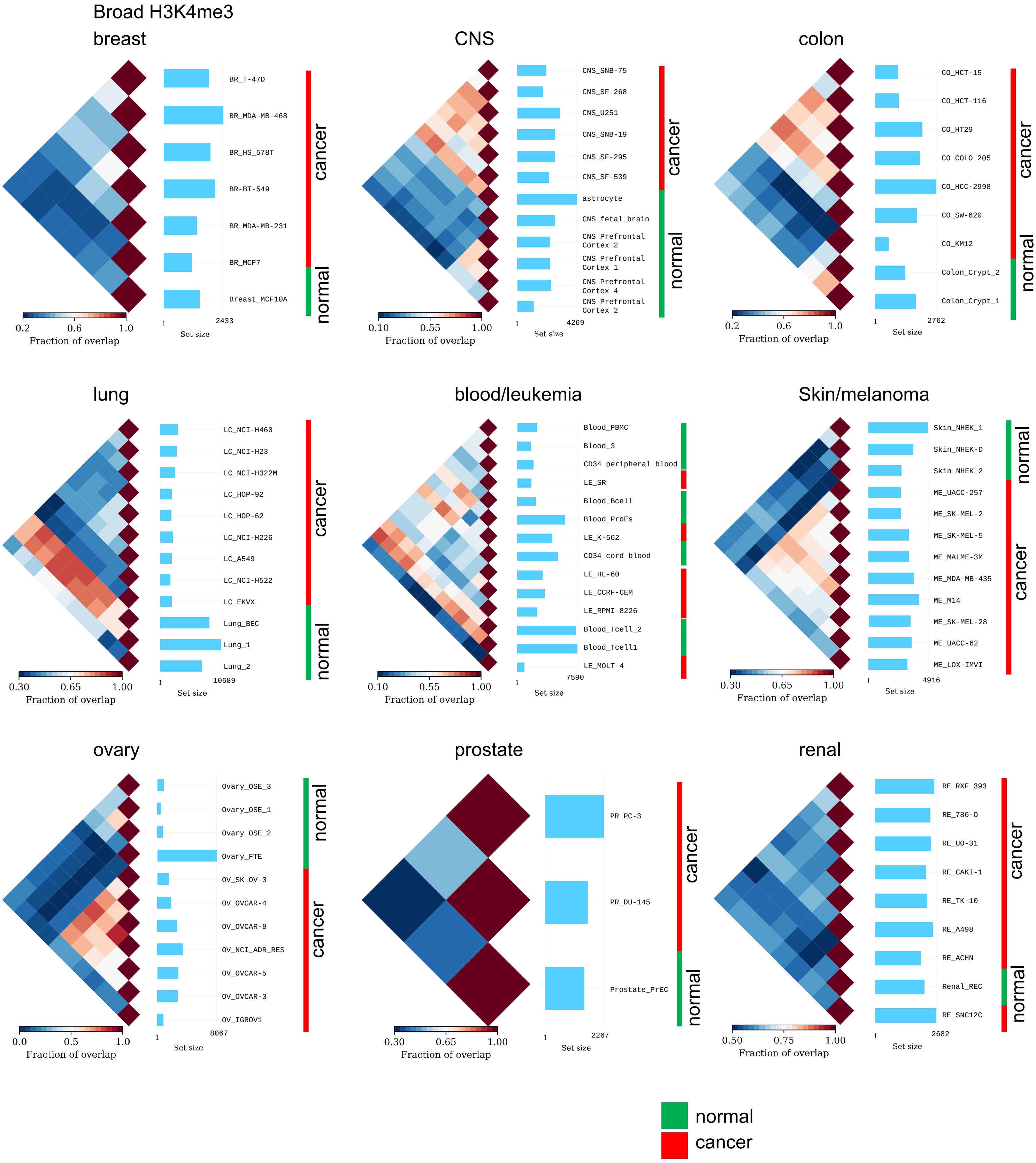

Supplementary Fig. 14

Supplementary Fig. 14. Intervene pairwise intersection of broad H3K4me3 regions in cancer and normal cells. Pairwise intersection of broad H3K4me3 enriched regions in cancer and normal cells[1-15]. Heat maps of pairwise intersections using Jaccard statistics of broad H3K4me3 regions was generated using Intervene. Cancer cells representing 9 types of cancer were compared to normal cells.

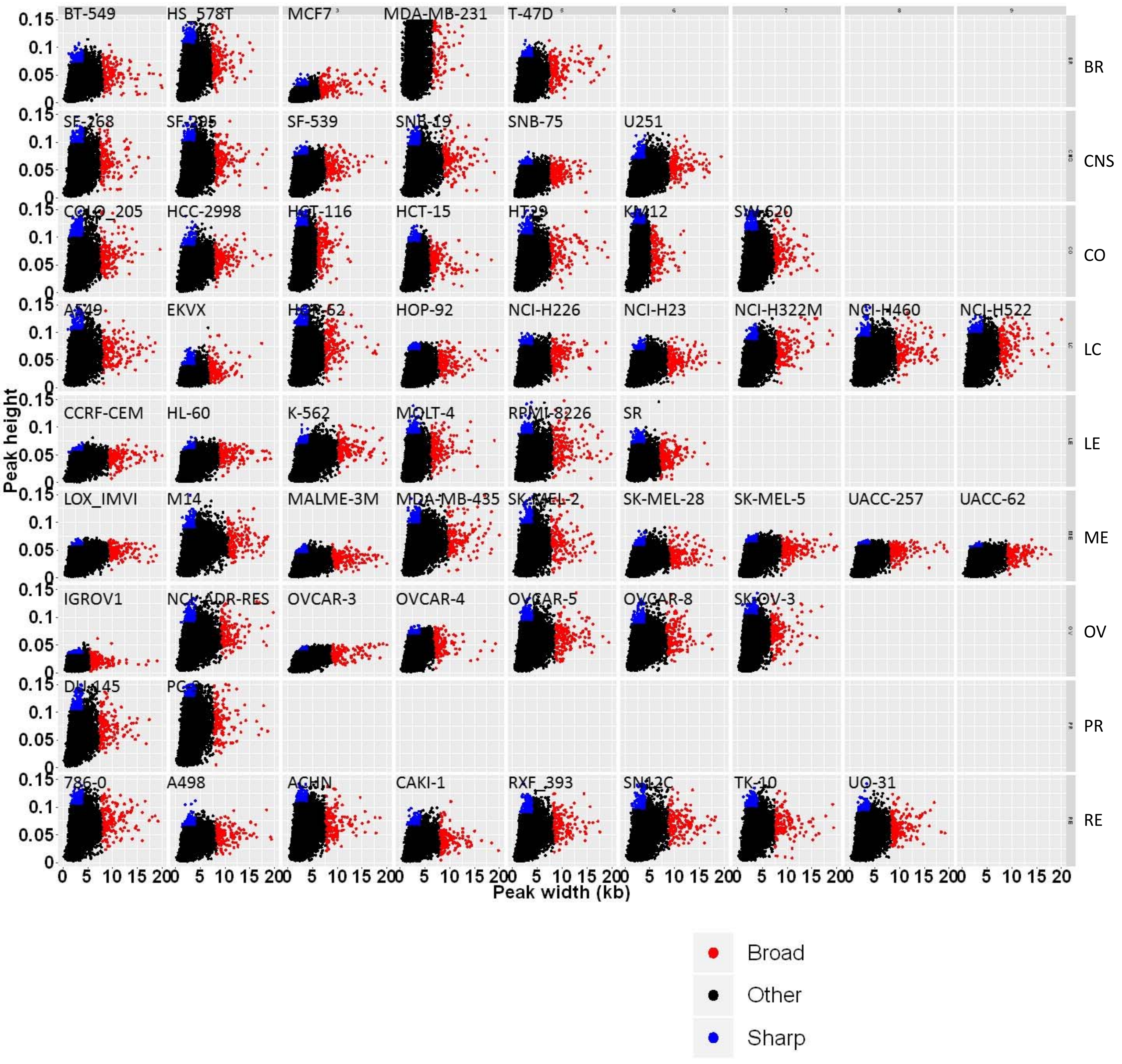

Supplementary Fig. 15

Supplementary Fig. 15. Promoter associated H3K4me3 domain width and height for 60 cancer cells. Scatter plots of H3K4me3 height (y-axis) and width (x-axis). Blue and red points represent sharp and broad peaks, respectively.

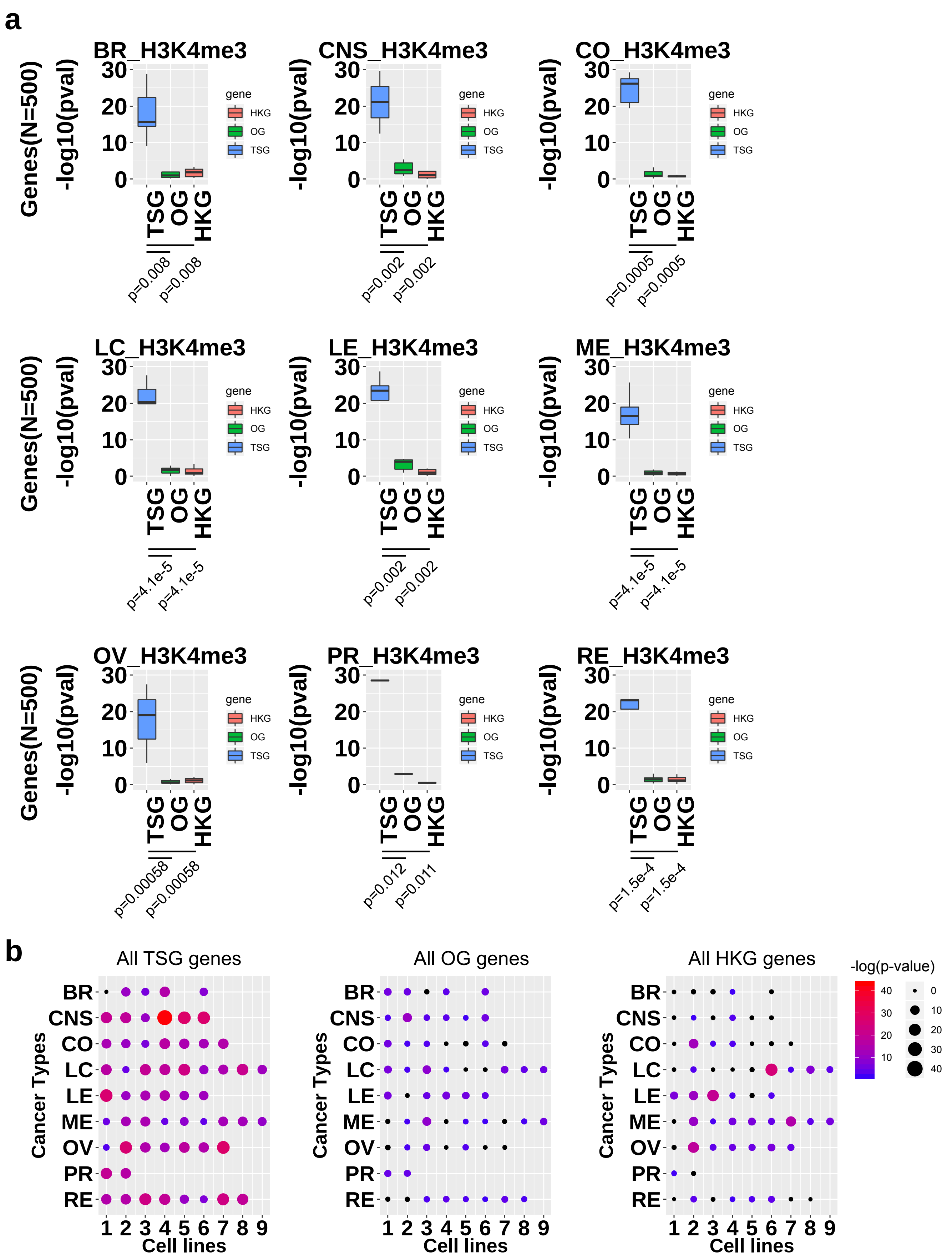

Supplementary Fig. 16

Supplementary Fig. 16. Association of broad H3K4me3 domains with tumor suppressor genes. (**a**) Boxplot of enrichment p-values (y-axis) of tumor suppressors, oncogenes, and housekeeping genes for genes associated with promoter broad H3K4me3 peaks for each cancer cell line. P-values (y-axis) were determined using two-sided Fisher's exact tests. The top 500 tumor suppressors, oncogenes, and 500 random housekeeping genes were used for this analysis. Boxplots indicate the 1st and 3rd quartiles (25th and 75th percentile, upper and lower bounds), 2nd quartile (centre), and minima-maxima (1.5*interquartile range, whiskers). P-values (x-axis) were calculated using two-sided Kolmogorov–Smirnov tests. **(b)** Bubble plots indicating enrichment p-values of TSG, OG, and housekeeping genes for genes associated with broad H3K4me3 for each cancer cell line. All tumor suppressor genes were used for this analysis. Source data are provided as a Source Data file.

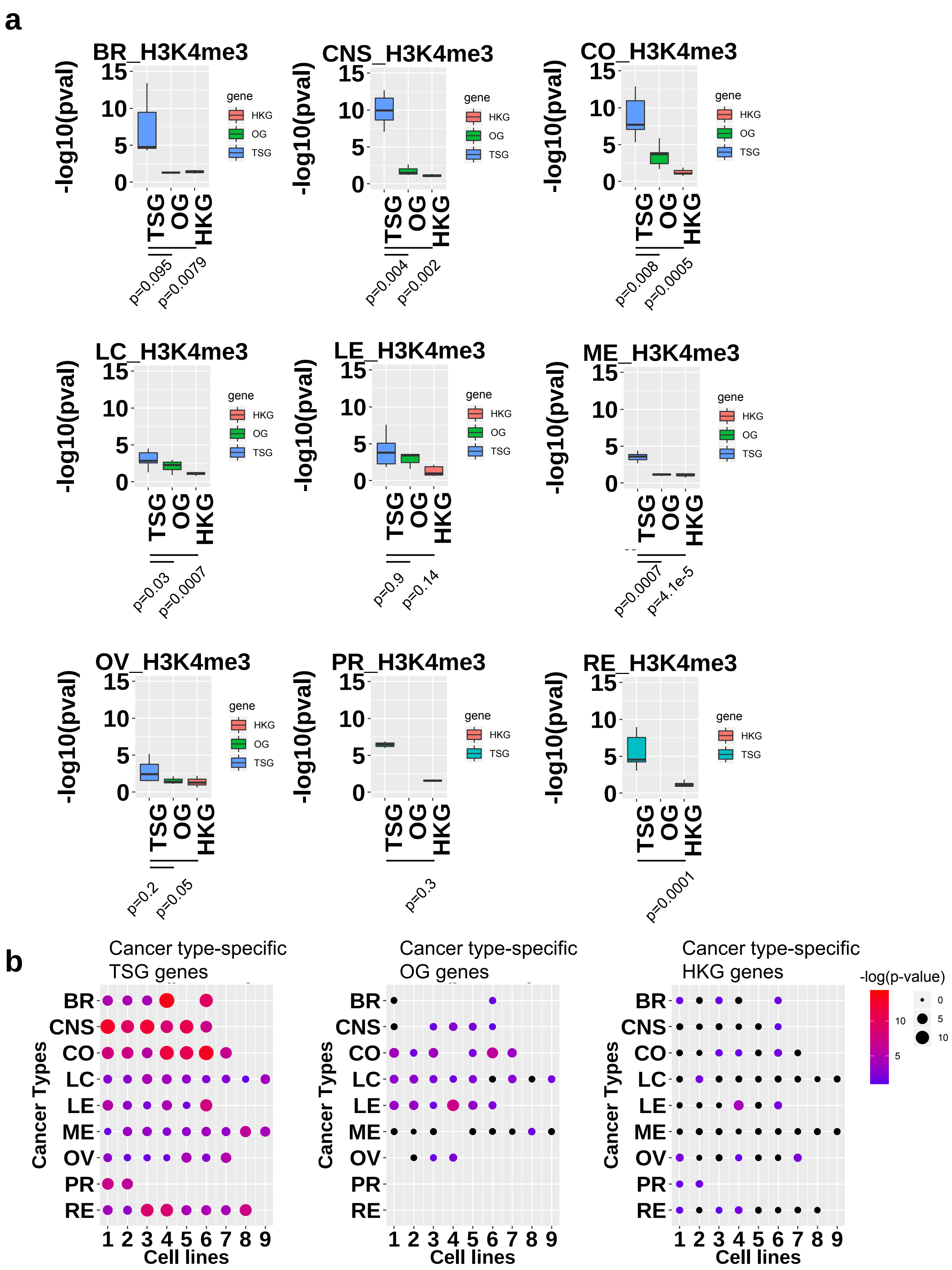

Supplementary Fig. 17

Supplementary Fig. 17. Association of broad H3K4me3 domains with cancer type-specific tumor suppressor genes. (**a**) Boxplot of enrichment p-values (y-axis) of cancer type-specific tumor suppressor genes (TSG), oncogenes (OG), and housekeeping genes (HKG) for genes associated with promoter broad H3K4me3 peaks for each cancer cell line. P-values (y-axis) were determined using two-sided Fisher's exact tests. Cancer type-specific tumor suppressors, oncogenes, and random housekeeping genes were used for this analysis. Boxplots indicate the 1st and 3rd quartiles (25th and 75th percentile, upper and lower bounds), 2nd quartile (centre), and minima-maxima (1.5*interquartile range, whiskers). P-values (x-axis) were calculated using two-sided Kolmogorov–Smirnov tests. (**b**) Bubble plots indicating enrichment p-values of cancer type-specific tumor suppressors (TSG), oncogenes (OG), and housekeeping genes for genes associated with broad H3K4me3 for each cancer cell line. Cancer type-specific tumor suppressor genes and oncogenes listed in Table S4 were used for this analysis. P-values were determined using two-sided Fisher's exact tests. Source data are provided as a Source Data file.

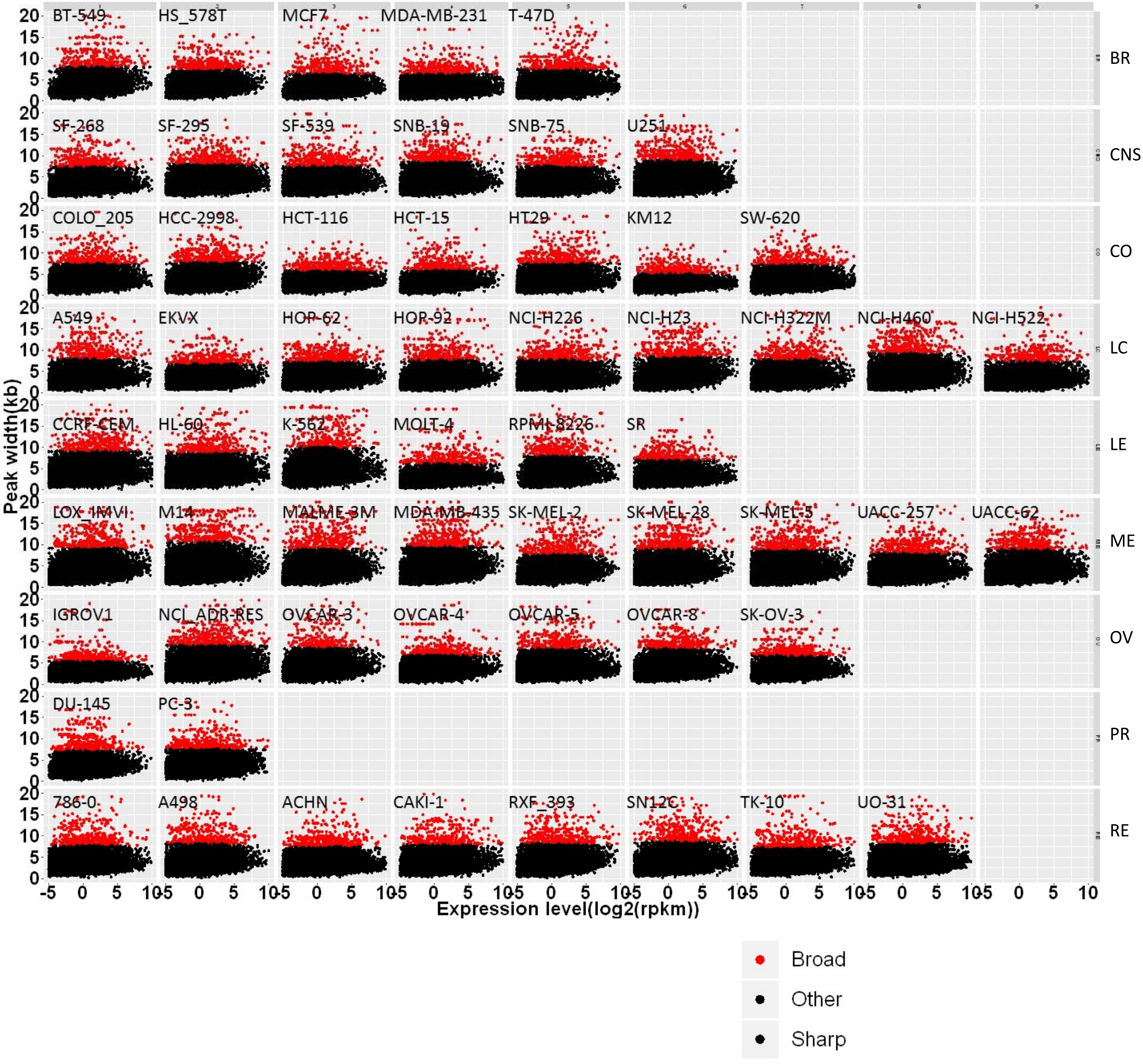

Supplementary Fig. 18

Supplementary Fig. 18. Promoter associated H3K4me3 peak width and gene expression level across 60 cancer cells. Scatter plots of H3K4me3 peak width (y-axis) and gene expression level (x-axis) for 60 cancer cell lines. Red points indicate broad peaks.

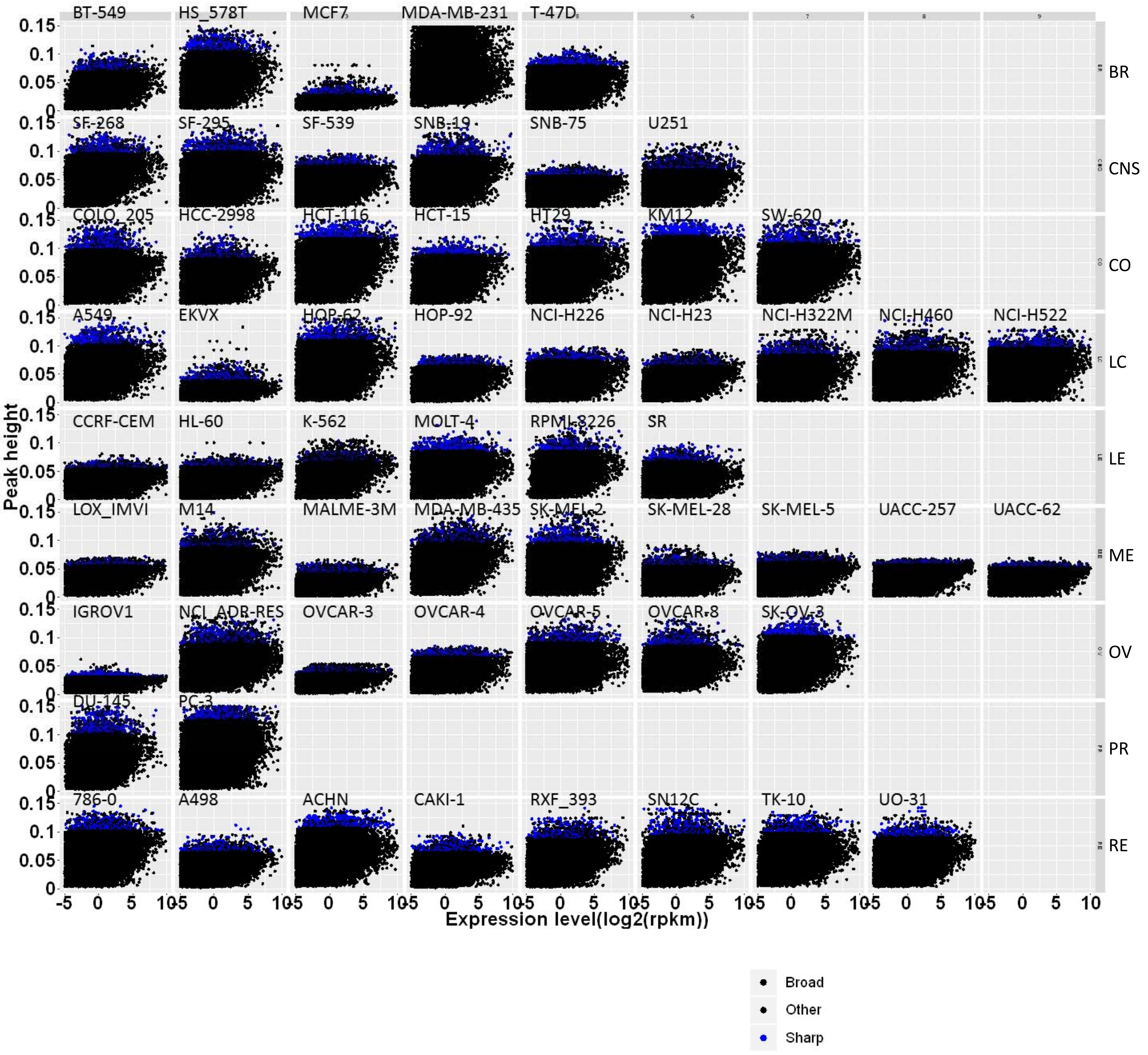

Supplementary Fig. 19

Supplementary Fig. 19. Promoter associated H3K4me3 peak height and gene expression level across 60 cancer cells. Scatter plots of H3K4me3 peak height (y-axis) and gene expression level (x-axis) for 60 cancer cell lines. Blue points indicate sharp peaks.

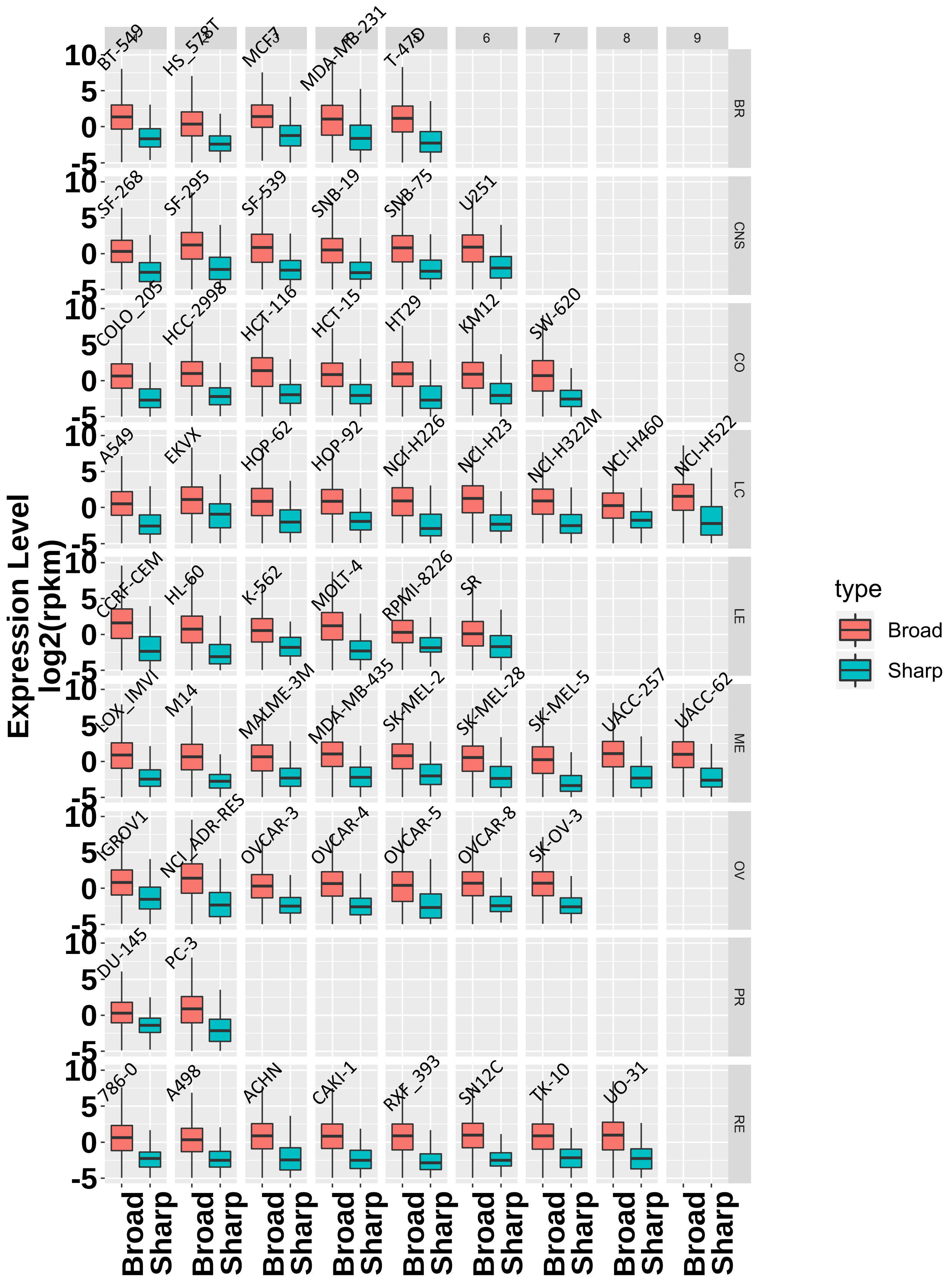

p-value for all broad vs. sharp <2.2e-16

Supplementary Fig. 20

Supplementary Fig. 20. Expression analysis of genes associated with sharp or broad H3K4me3 peaks in NCI-60 cancer cells. Boxplots showing expression level of genes associated with broad or sharp H3K4me3 peaks for 60 cell lines. Boxplots indicate the 1st and 3rd quartiles (25th and 75th percentile, upper and lower bounds), 2nd quartile (centre), and minima-maxima (1.5*interquartile range, whiskers). P-value for all $P < 1x10^{-16}$. P-values were calculated using two-sided Kolmogorov–Smirnov tests. Source data are provided as a Source Data file.

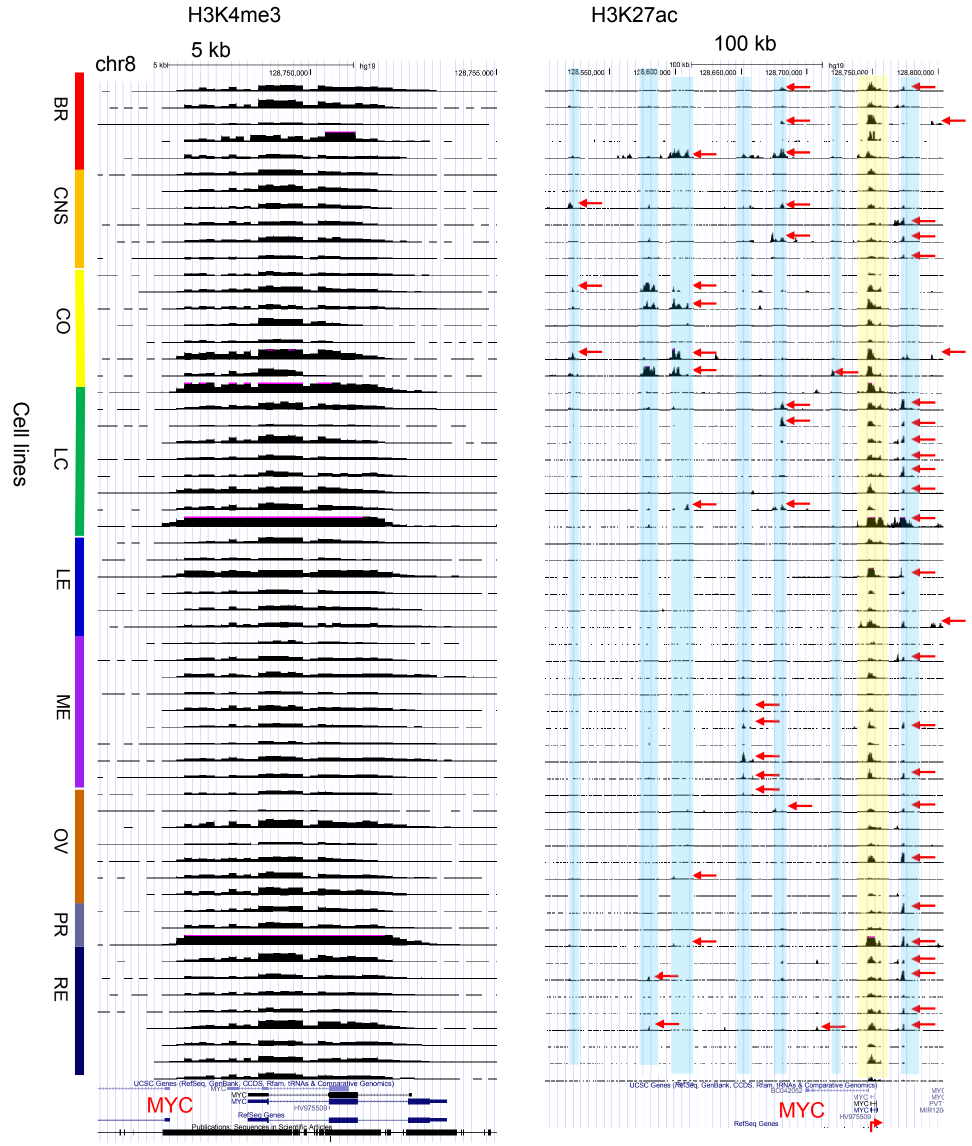
H3K4me3
H3K27ac
5 kb
100 kb
chr8
Cell lines
BR
CNS
CO
LC
LE
ME
OV
PR
RE
MYC
MYC

Supplementary Fig. 21. View of H3K4me3 and H3K27ac at a broad H3K4me3 domain. UCSC browser view of H3K4me3 (left; scale: 0-0.15 norm. tag density) and H3K27ac (right; scale: 0-0.05 norm. tag density) distributions at a representative broad H3K4me3 domain in 60 cancer cells (scale: 0-0.05 norm. tag density). Red arrows indicate variable H3K27ac levels and distributions.

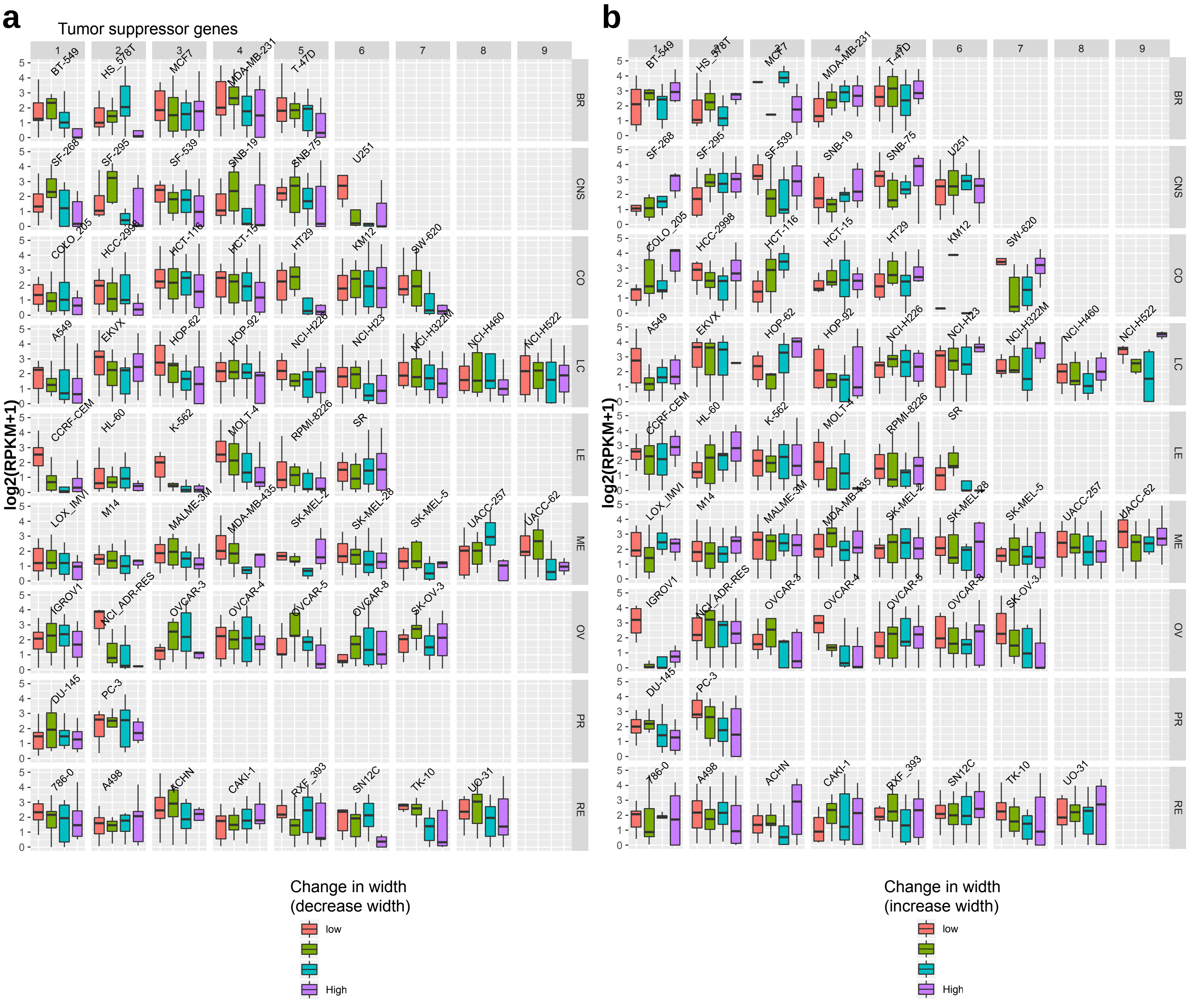

Supplementary Fig. 22

Supplementary Fig. 22. Expression analysis of tumor suppressor genes associated with broad H3K4me3 peaks in NCI-60 cancer cells. Boxplots showing expression level (y-axis) of tumor suppressor genes associated with conserved broad H3K4me3 peaks for 60 cell lines. Conserved H3K4me3 peaks that intersect TSS regions were defined as those found in more than 50% of cancer cell lines (>30 cell lines). Shortening of H3K4me3 (decrease width) was defined as a decrease in breadth less than 500 bp relative to the average breadth across 60 cancer cell lines, while lengthening of H3K4me3 (increase width) was defined as an increase in breadth greater than 500 bp relative to the average across 60 cancer cell lines. Boxplots indicate the 1st and 3rd quartiles (25th and 75th percentile, upper and lower bounds), 2nd quartile (centre), and minima-maxima (1.5*interquartile range, whiskers). (**a**) Genes associated with H3K4me3 peaks that shorten were sorted into four groups based on shortening of H3K4me3 (red, least shortening 25%; purple, greatest shortening 25%). (**b**) Genes associated with H3K4me3 peaks that lengthen were sorted into four groups based on lengthening of H3K4me3 (red, least lengthening 25%; purple, greatest lengthening 25%). Source data are provided as a Source Data file.

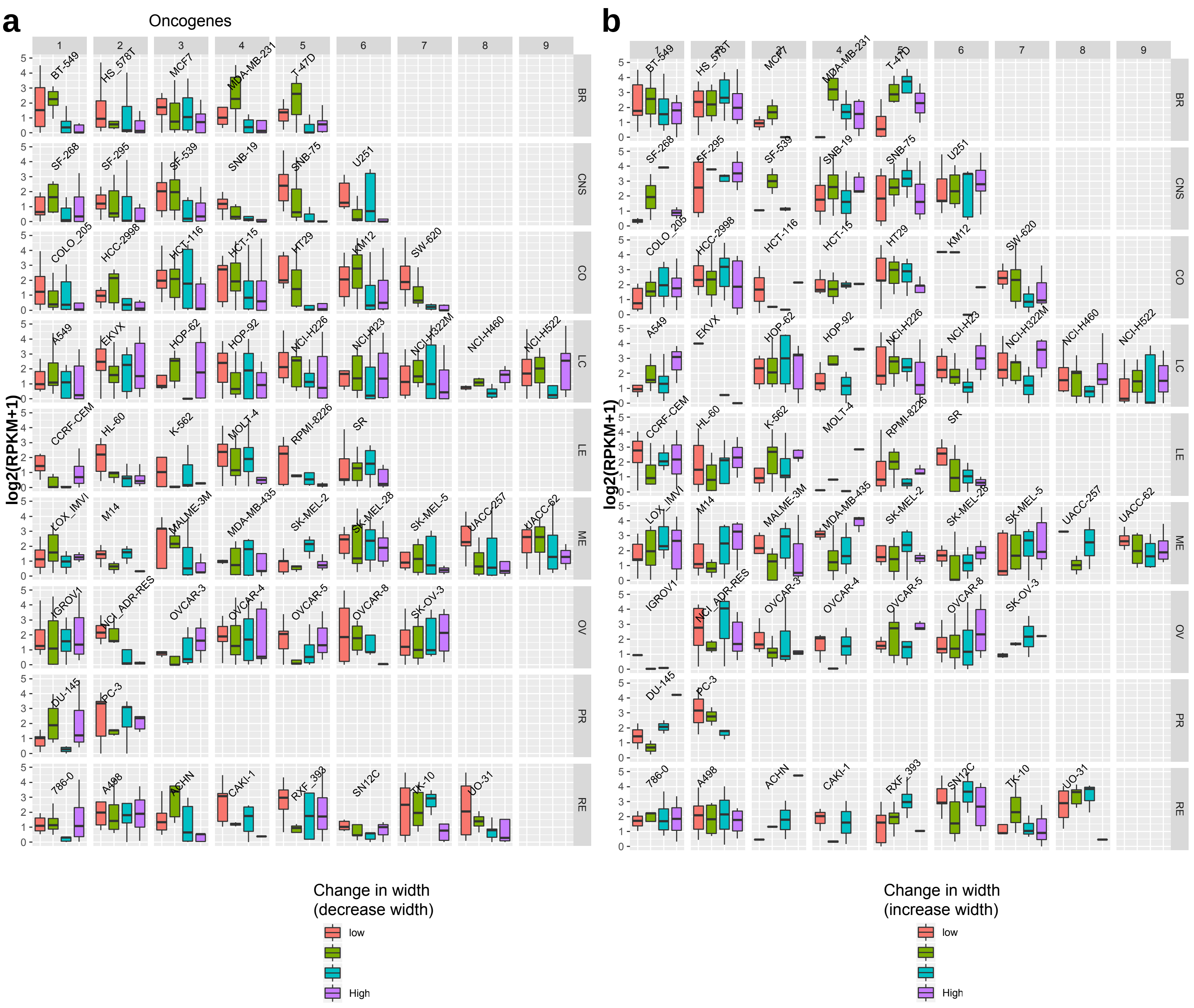

Supplementary Fig. 23

Supplementary Fig. 23. Expression analysis of oncogenes associated with broad H3K4me3 peaks in NCI-60 cancer cells. Boxplots showing expression level (y-axis) of oncogenes associated with conserved broad H3K4me3 peaks for 60 cell lines. Conserved H3K4me3 peaks that intersect TSS regions were defined as those found in more than 50% of cancer cell lines (>30 cell lines). Shortening of H3K4me3 (decrease width) was defined as a decrease in breadth less than 500 bp relative to the average breadth across 60 cancer cell lines, while lengthening of H3K4me3 (increase width) was defined as an increase in breadth greater than 500 bp relative to the average across 60 cancer cell lines. Boxplots indicate the 1st and 3rd quartiles (25th and 75th percentile, upper and lower bounds), 2nd quartile (centre), and minima-maxima (1.5*interquartile range, whiskers). (**a**) Genes associated with H3K4me3 peaks that shorten were sorted into four groups based on shortening of H3K4me3 (red, least shortening 25%; purple, greatest shortening 25%). (**b**) Genes associated with H3K4me3 peaks that lengthen were sorted into four groups based on lengthening of H3K4me3 (red, least lengthening 25%; purple, greatest lengthening 25%). Source data are provided as a Source Data file.

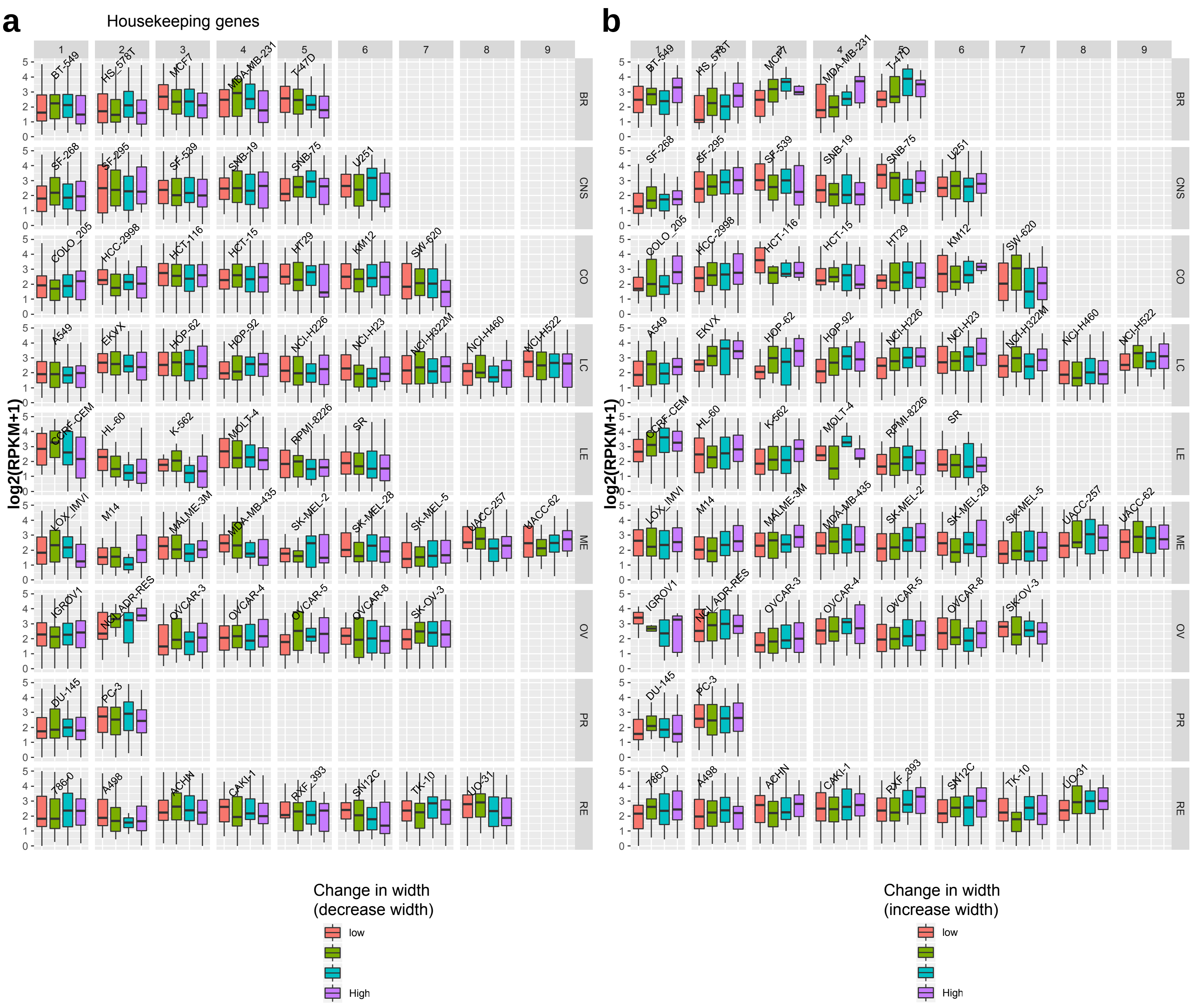

Supplementary Fig. 24

Supplementary Fig. 24. Expression analysis of housekeeping associated with broad H3K4me3 peaks in NCI-60 cancer cells. Boxplots showing expression level (y-axis) of housekeeping genes associated with conserved broad H3K4me3 peaks for 60 cell lines. Conserved H3K4me3 peaks that intersect TSS regions were defined as those found in more than 50% of cancer cell lines (>30 cell lines). Shortening of H3K4me3 (decrease width) was defined as a decrease in breadth less than 500 bp relative to the average breadth across 60 cancer cell lines, while lengthening of H3K4me3 (increase width) was defined as an increase in breadth greater than 500 bp relative to the average across 60 cancer cell lines. Boxplots indicate the 1st and 3rd quartiles (25th and 75th percentile, upper and lower bounds), 2nd quartile (centre), and minima-maxima (1.5*interquartile range, whiskers). (**a**) Genes associated with H3K4me3 peaks that shorten were sorted into four groups based on shortening of H3K4me3 (red, least shortening 25%; purple, greatest shortening 25%). (**b**) Genes associated with H3K4me3 peaks that lengthen were sorted into four groups based on lengthening of H3K4me3 (red, least lengthening 25%; purple, greatest lengthening 25%). Source data are provided as a Source Data file.

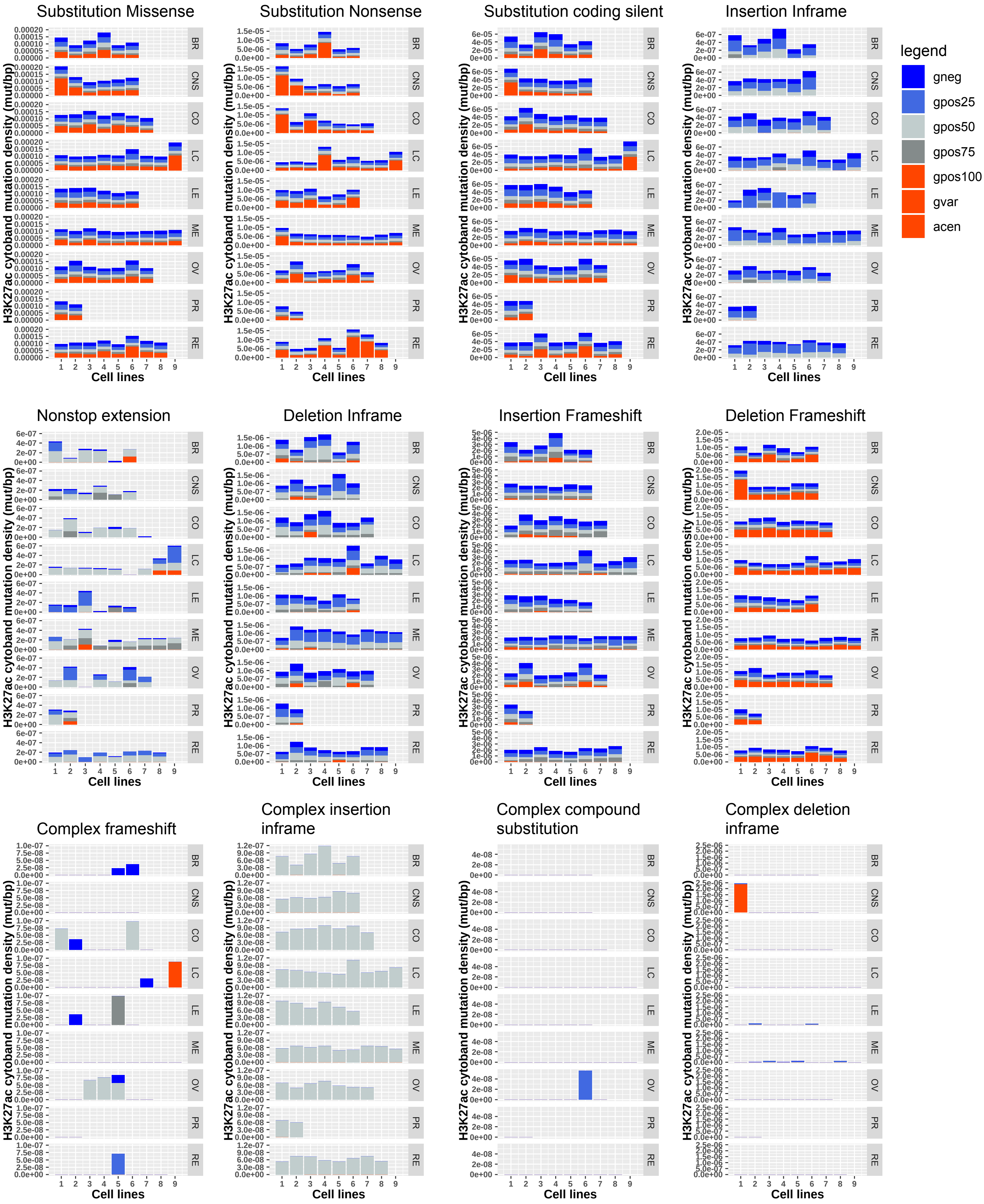

Supplementary Fig. 25

Supplementary Fig. 25. Mutation analysis of H3K27ac cytogenetic banding patterns. Stacked barplots showing cosmic mutation type density (mutation/bp) at chromatin regions overlapping cytogenetic banding patterns and H3K27ac peaks across 60 cancer cells. Cytobands were obtained from the UCSC genome browser. Source data are provided as a Source Data file.

a

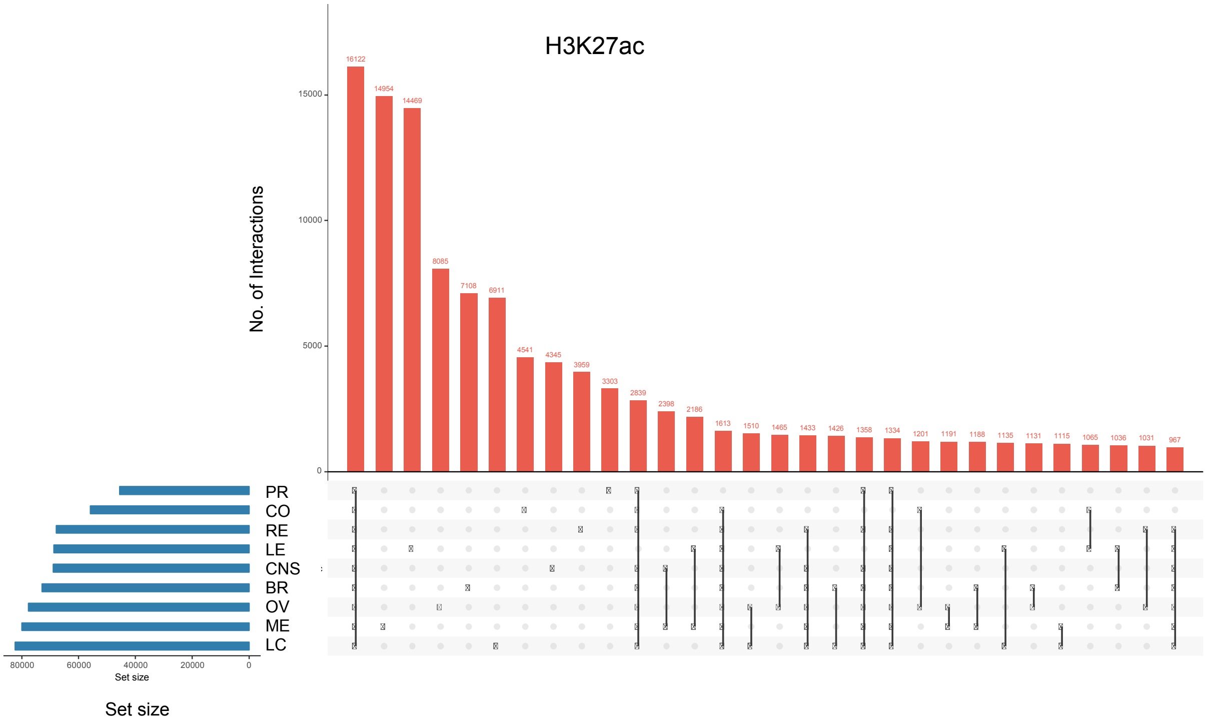

b

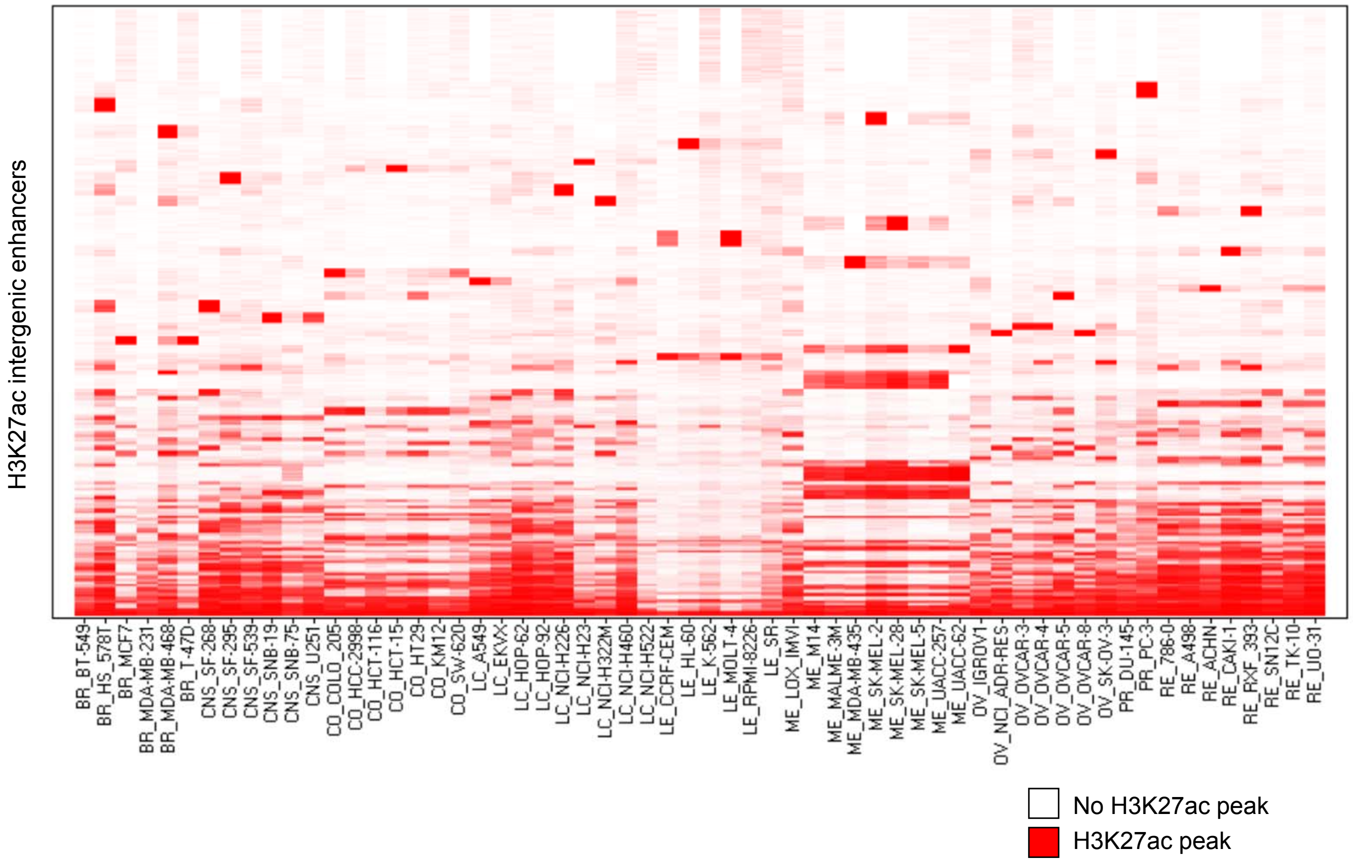

Supplementary Fig. 26

Supplementary Fig. 26. H3K27ac profiling in cancer cells. (**a**) Intervene UpSet plot of intersection of intergenic H3K27ac peaks for 9 types of cancer. (**b**) All intergenic H3K27ac enhancer modules across 60 cell lines. Heatmap of k-means clustered H3K27ac-marked intergenic enhancers (red: enhancer; white: no enhancer). Source data are provided as a Source Data file.

H3K27ac intergenic enhancers

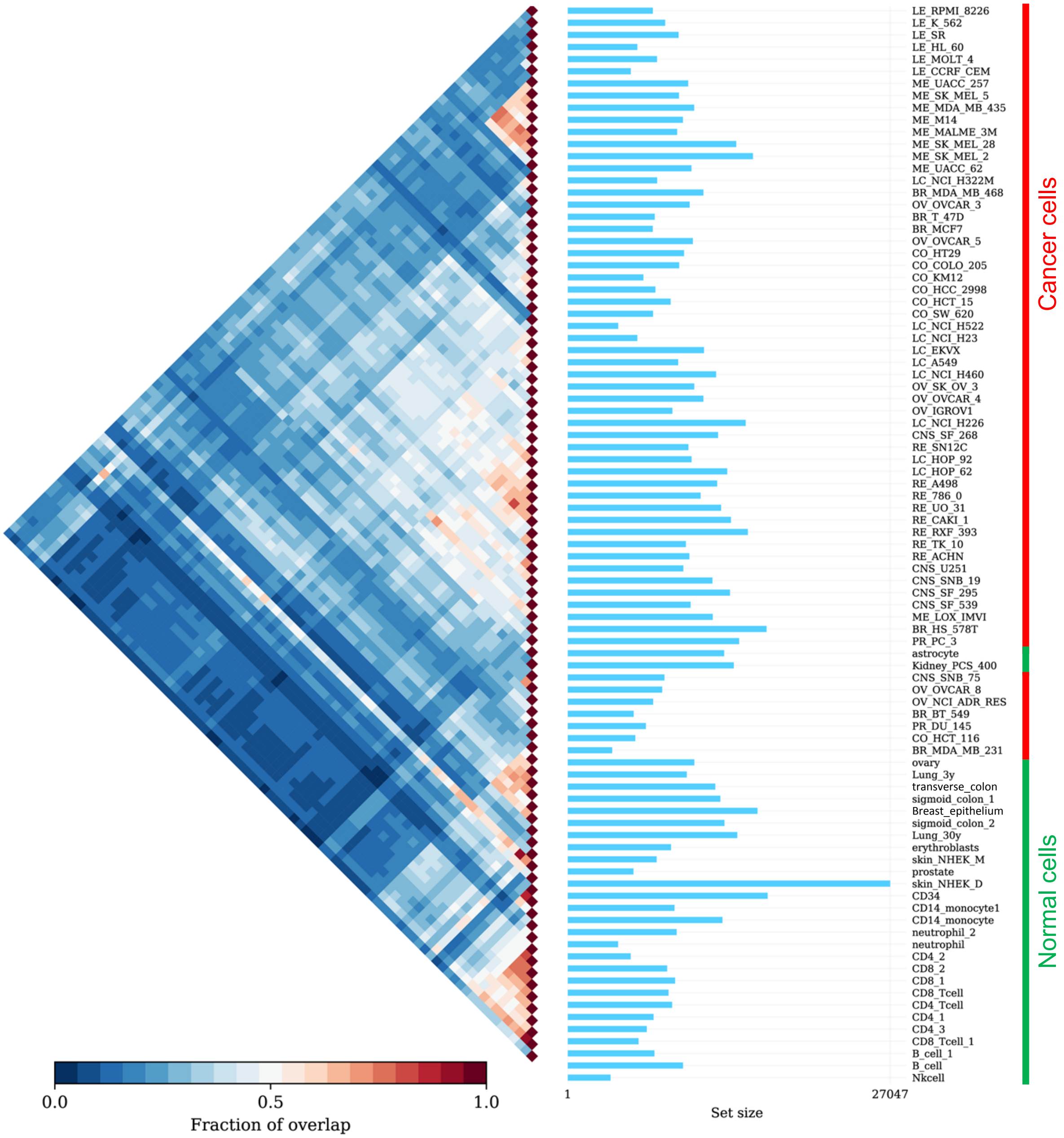

Supplementary Fig. 27. Intervene pairwise intersection of intergenic H3K27ac enhancer regions in cancer and normal cells. Intervene pairwise intersection heat map of intergenic H3K27ac peaks for 60 cancer cells and normal cells [2,4,13,16-20]. Heat map of pairwise intersection using Jaccard statistics of intergenic H3K27ac regions was generated using Intervene.

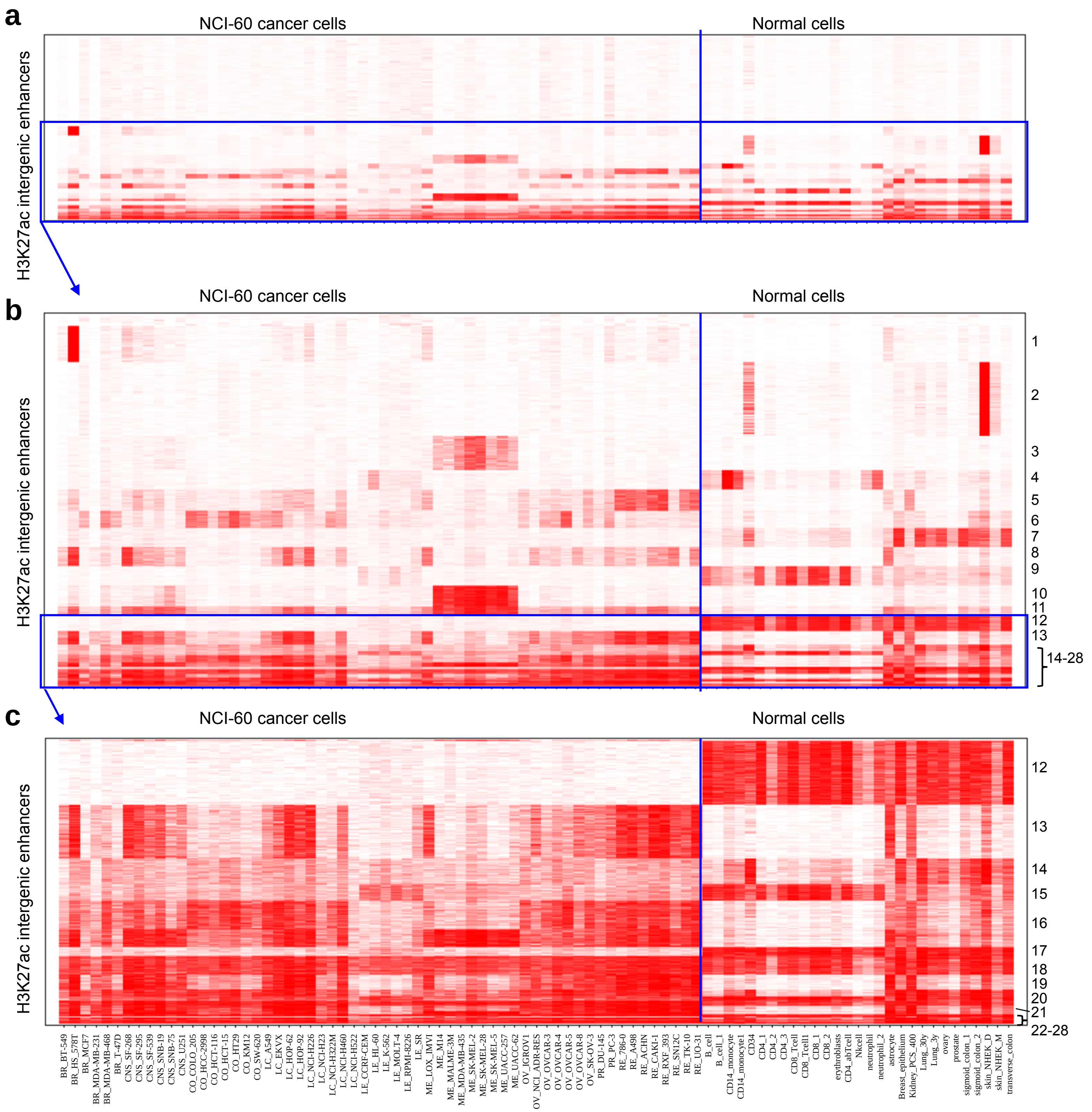

Supplementary Fig. 28

Supplementary Fig. 28. H3K27ac profiling in cancer and normal cells. (a) All intergenic H3K27ac enhancer regions across 60 cancer cell lines and normal cells. Heatmap of k-means clustered H3K27ac-marked intergenic enhancers (red: enhancer; white: no enhancer). **(b)** Higher magnification of clusters shown in (a). **(c)** Higher magnification of clusters shown in (b). Source data are provided as a Source Data file.

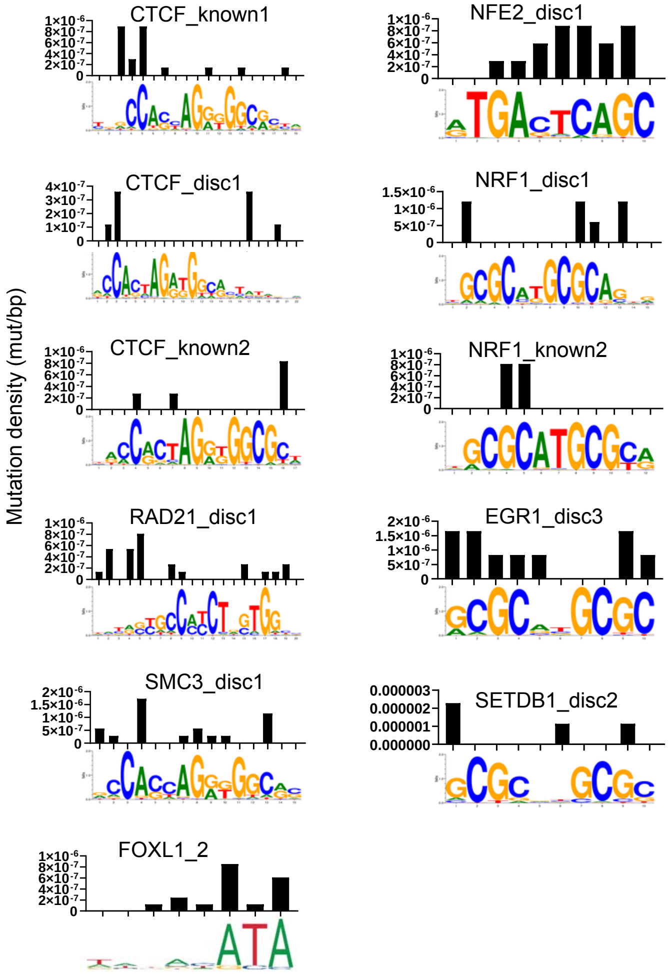
Mutation density (mut/bp)
CTCF_known1
CTCF_disc1
CTCF_known2
RAD21_disc1
SMC3_disc1
FOXL1_2
NFE2_disc1
NRF1_disc1
NRF1_known2
EGR1_disc3
SETDB1_disc2

Supplementary Fig. 29. Mutation density in TF binding motifs. Mutation density (mutation/bp) in genome-wide DNA sequences containing transcription factor recognition motifs across 60 cancer cell lines. Sequence logos of position frequency matrix (PFM) TF binding motifs are shown. Source data are provided as a Source Data file.

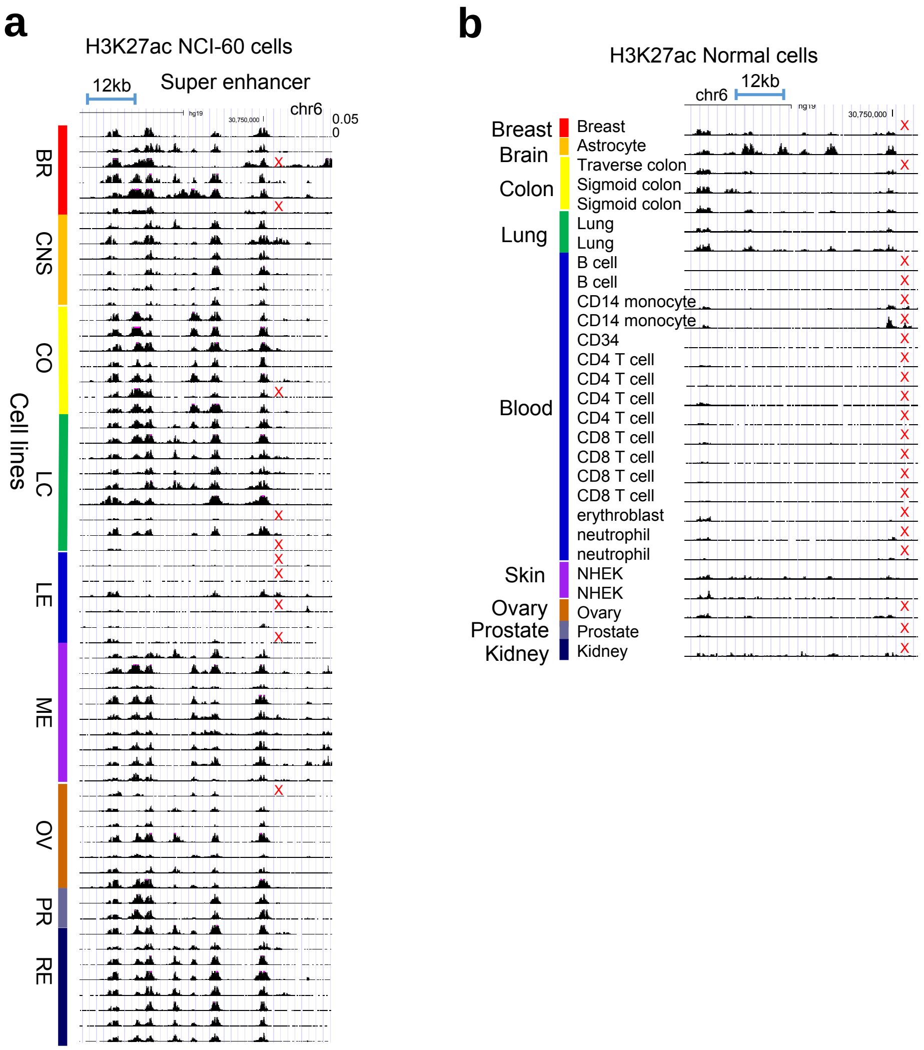

Supplementary Fig. 30

Supplementary Fig. 30. H3K27ac at a representative cancer type-specific super enhancer cluster. UCSC browser view of H3K27ac distributions at a representative super enhancer cluster in (**a**) cancer cells and (**b**) normal cells[2,4,16-20] (scale: 0-0.05 norm. tag density). Red 'x' indicates absence of a super enhancer.

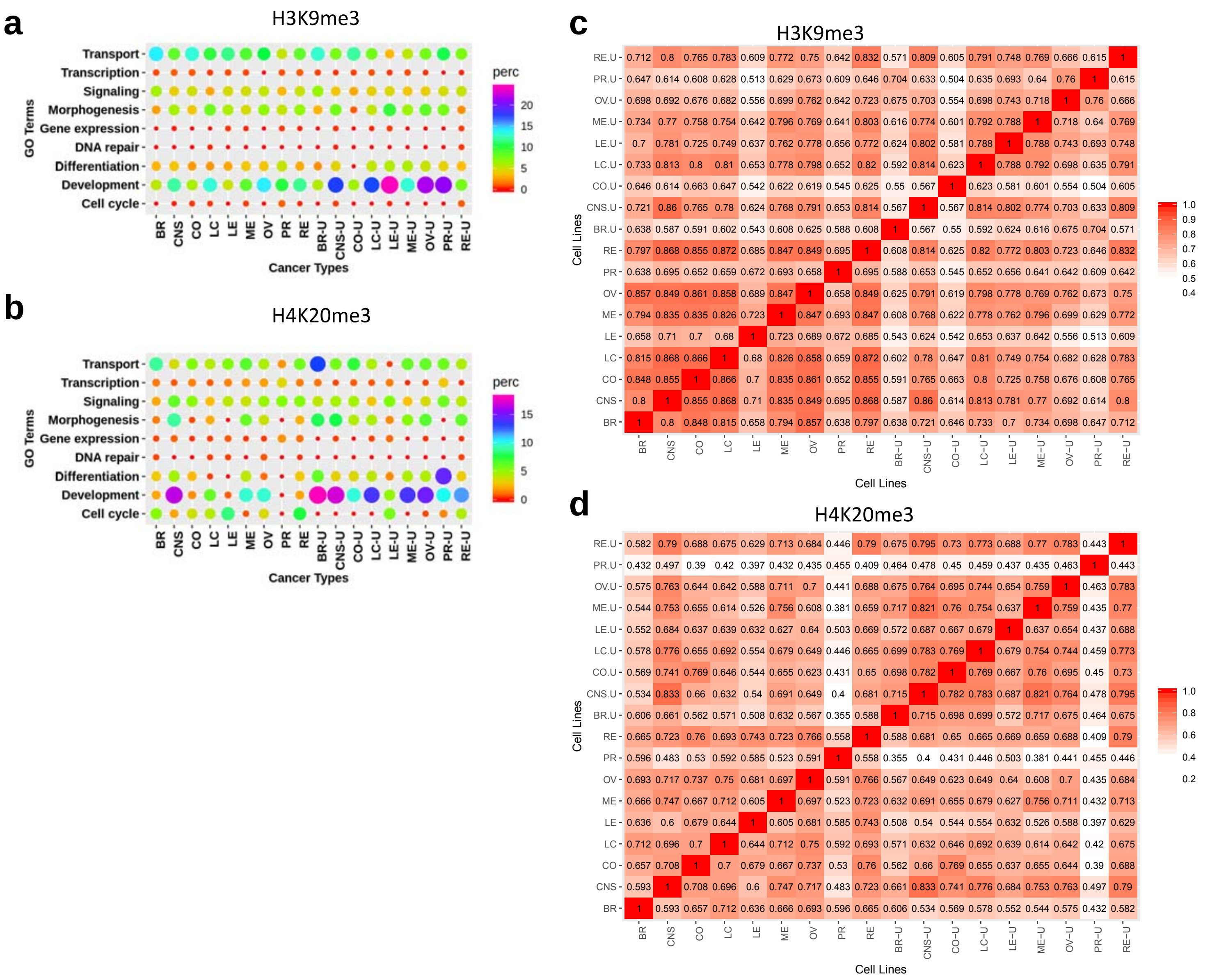

Supplementary Fig. 31

Supplementary Fig. 31. Gene ontology functional annotation of H3K9me3 and H4K20me3 peaks. H3K9me3 and H4K20me3 regions nearby TSS of genes (within 10 kb) were functionally annotated using DAVID. (**a-b**) Bubble plots (top) shows enrichment of top biological process GO terms identified from all peaks and cancer-type specific peaks from 9 cancer types (u: unique). GoSemSim (bottom) semantic similarity analysis. All (**c**) H3K9me3 and (**d**) H4K20me3 peaks for 60 cell lines and cancer-type specific peaks were annotated. Source data are provided as a Source Data file.

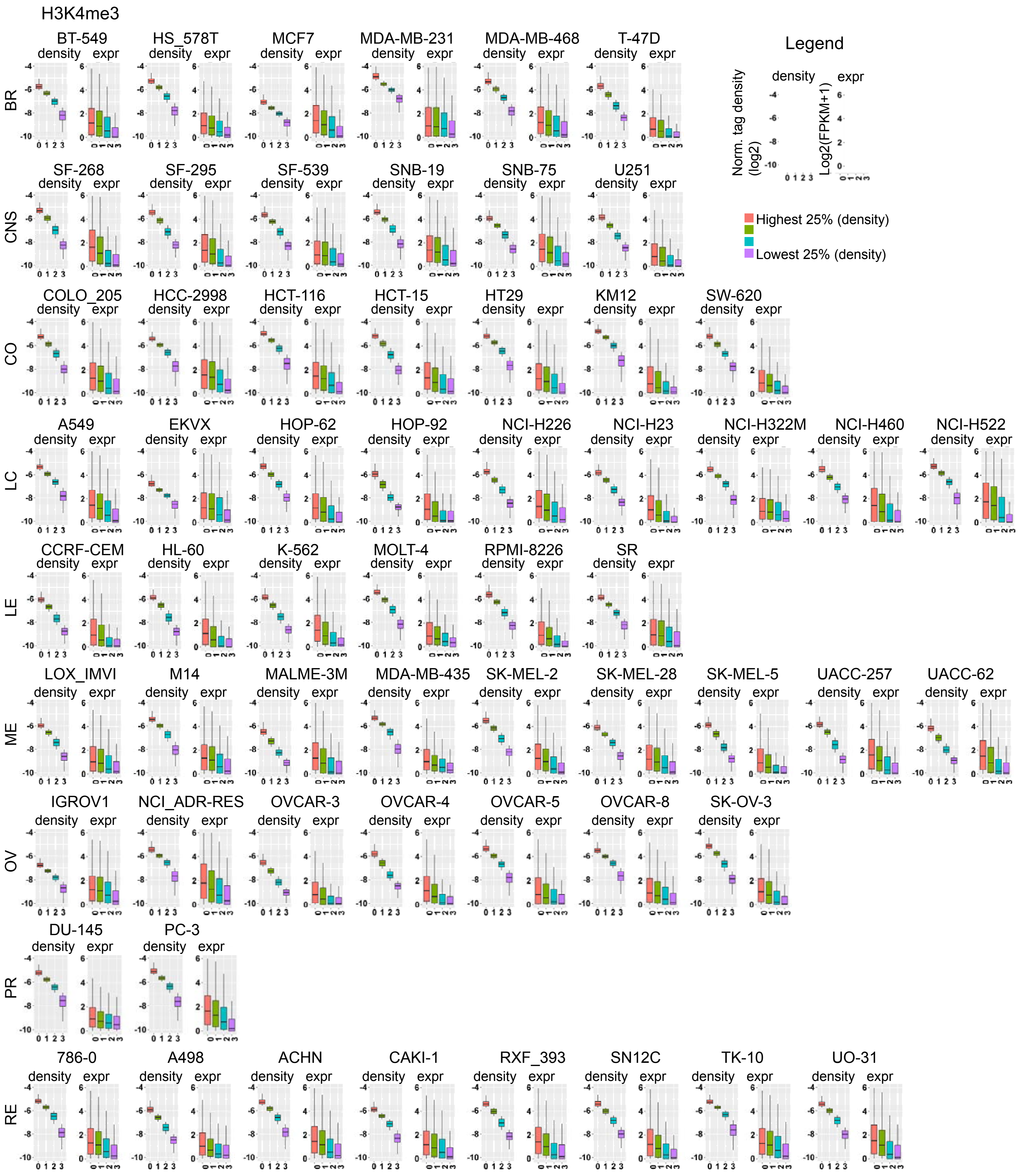

Supplementary Fig. 32

Supplementary Fig. 32. Correlation between H3K4me3 and gene expression. Boxplots of H3K4me3 densities (log2 normalized tag density) and RNA-Seq expression [log2(FPKM+1)] in 60 cancer cell lines representing 9 cancer types. Genes whose promoter contain H3K4me3 peaks were divided into quartiles (0, highest 25% density; 3, lowest 25% density) based on their H3K4me3 density in the respective cancer cell line. Boxplots indicate the 1st and 3rd quartiles (25th and 75th percentile, upper and lower bounds), 2nd quartile (centre), and minima-maxima (1.5*interquartile range, whiskers).

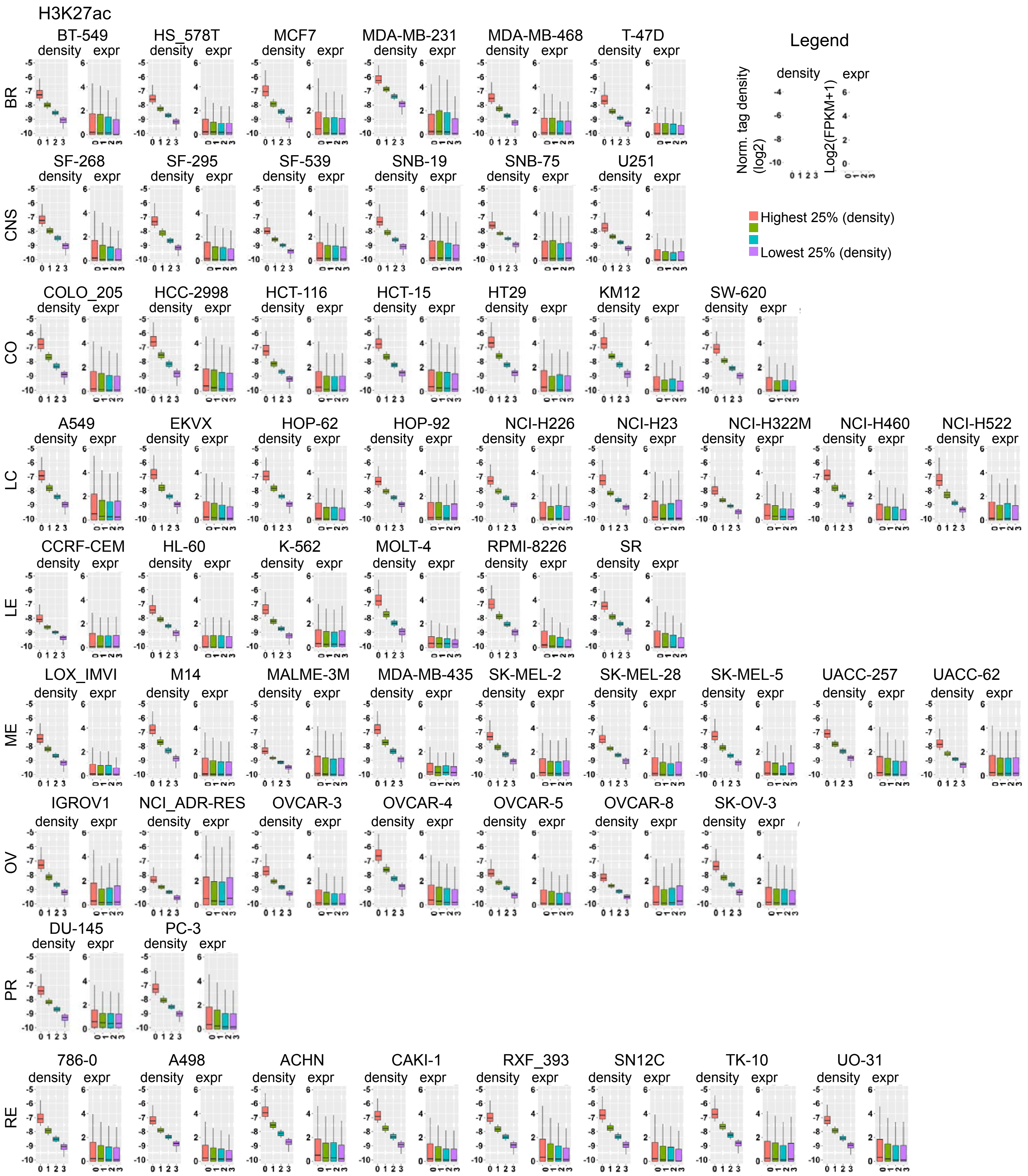

Supplementary Fig. 33

Supplementary Fig. 33. Correlation between H3K27ac and gene expression. Boxplots of intergenic H3K27ac densities (log2 normalized tag density) and RNA-Seq expression [log2(FPKM+1)] in 60 cancer cell lines representing 9 cancer types. Genes associated with intergenic H3K27ac peaks were divided into quartiles (0, highest 25% density; 3, lowest 25% density) based on their H3K27ac density in the respective cancer cell line. Boxplots indicate the 1st and 3rd quartiles (25th and 75th percentile, upper and lower bounds), 2nd quartile (centre), and minima-maxima (1.5*interquartile range, whiskers).

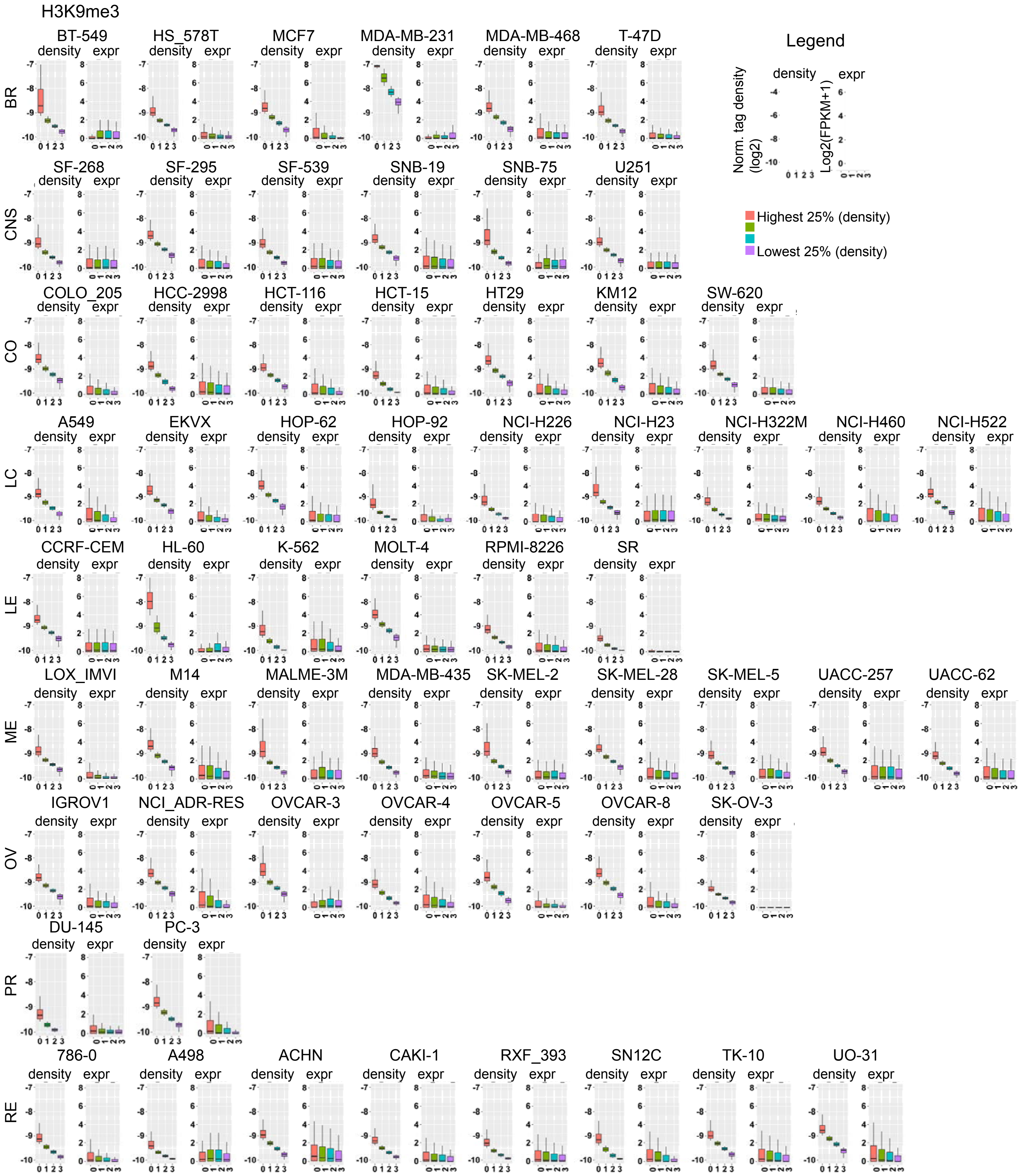

Supplementary Fig.34

Supplementary Fig. 34. Correlation between H3K9me3 and gene expression. Boxplots of H3K9me3 densities (log2 normalized tag density) and RNA-Seq expression [log2(FPKM+1)] in 60 cancer cell lines representing 9 cancer types. Genes associated with H3K9me3 peaks were divided into quartiles (0, highest 25% density; 3, lowest 25% density) based on their H3K9me3 density in the respective cancer cell line.

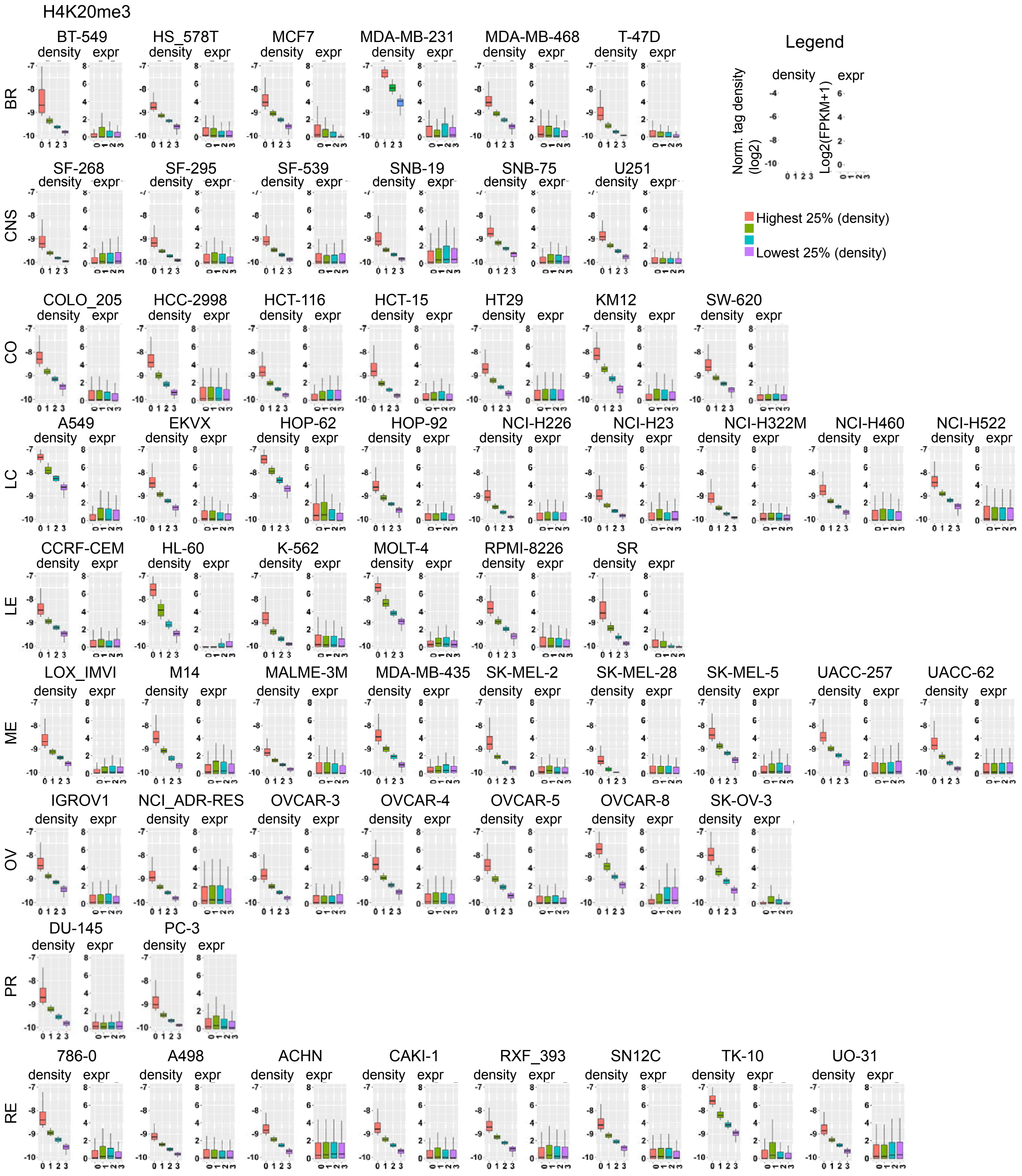

Supplementary Fig.35

Supplementary Fig. 35. Correlation between H4K20me3 and gene expression. Boxplots of H4K20me3 densities (log2 normalized tag density) and RNA-Seq expression [log2(FPKM+1)] in 60 cancer cell lines representing 9 cancer types. Genes associated with H4K20me3 peaks were divided into quartiles (0, highest 25% density; 3, lowest 25% density) based on their H4K20me3 density in the respective cancer cell line. Boxplots indicate the 1st and 3rd quartiles (25th and 75th percentile, upper and lower bounds), 2nd quartile (centre), and minima-maxima (1.5*interquartile range, whiskers).

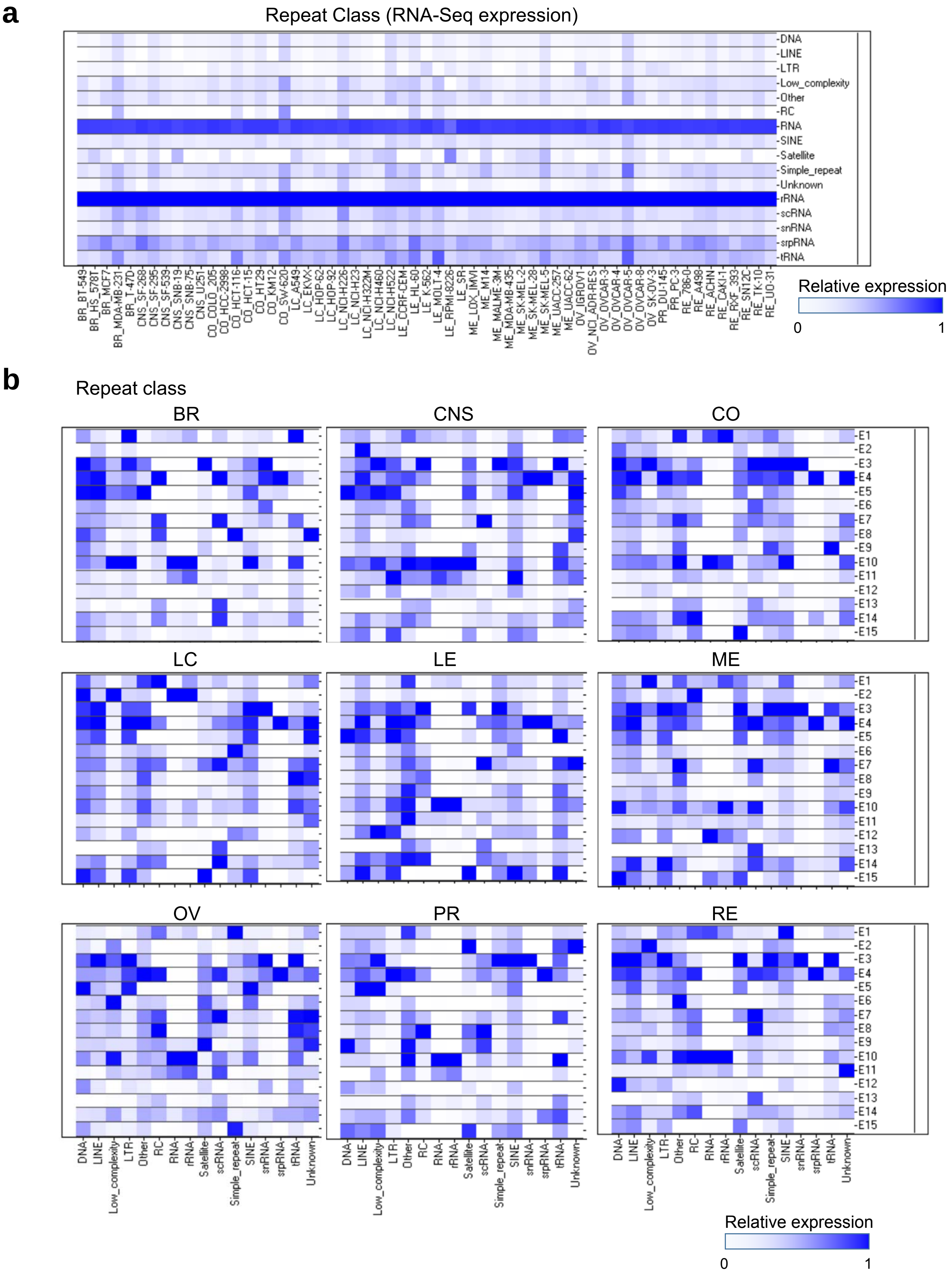

Supplementary Fig.36

Supplementary Fig. 36. Related to Figure 7. Expression of DNA repeat elements in cancer cells. (**a**) RNA-Seq expression of DNA repeat class members across cancer cell lines. Heatmap shows average RPKM values (column normalized 0 to 1). (**b**) Cancer type-specific RNA-Seq expression of repetitive DNA class members in the 15-state model. RNA-Seq expression (RPKM) of DNA repeat class members is column normalized (0 to 1).

Supplementary References

1 Cheung, I. *et al.* Developmental regulation and individual differences of neuronal H3K4me3 epigenomes in the prefrontal cortex. *Proc Natl Acad Sci U S A* **107**, 8824-8829, doi:10.1073/pnas.1001702107 (2010).

2 Bernstein, B. E. *et al.* The NIH Roadmap Epigenomics Mapping Consortium. *Nature biotechnology* **28**, 1045-1048, doi:10.1038/nbt1010-1045 (2010).

3 Akhtar-Zaidi, B. *et al.* Epigenomic enhancer profiling defines a signature of colon cancer. *Science* **336**, 736-739, doi:10.1126/science.1217277 (2012).

4 Consortium, E. P. An integrated encyclopedia of DNA elements in the human genome. *Nature* **489**, 57-74, doi:10.1038/nature11247 (2012).

5 Thurman, R. E. *et al.* The accessible chromatin landscape of the human genome. *Nature* **489**, 75-82, doi:10.1038/nature11232 (2012).

6 Bert, S. A. *et al.* Regional activation of the cancer genome by long-range epigenetic remodeling. *Cancer Cell* **23**, 9-22, doi:10.1016/j.ccr.2012.11.006 (2013).

7 Abraham, B. J., Cui, K., Tang, Q. & Zhao, K. Dynamic regulation of epigenomic landscapes during hematopoiesis. *BMC Genomics* **14**, 193, doi:10.1186/1471-2164-14-193 (2013).

8 Schmidl, C., Rendeiro, A. F., Sheffield, N. C. & Bock, C. ChIPmentation: fast, robust, low-input ChIP-seq for histones and transcription factors. *Nat Methods* **12**, 963-965, doi:10.1038/nmeth.3542 (2015).

9 Chen, K. *et al.* Broad H3K4me3 is associated with increased transcription elongation and enhancer activity at tumor-suppressor genes. *Nat Genet* **47**, 1149-1157, doi:10.1038/ng.3385 (2015).

10 Dincer, A. *et al.* Deciphering H3K4me3 broad domains associated with gene-regulatory networks and conserved epigenomic landscapes in the human brain. *Transl Psychiatry* **5**, e679, doi:10.1038/tp.2015.169 (2015).

11 Capell, B. C. *et al.* MLL1 is essential for the senescence-associated secretory phenotype. *Genes Dev* **30**, 321-336, doi:10.1101/gad.271882.115 (2016).

12 Kotzin, J. J. *et al.* The long non-coding RNA Morrbid regulates Bim and short-lived myeloid cell lifespan. *Nature* **537**, 239-243, doi:10.1038/nature19346 (2016).

13 Klein, R. H. *et al.* GRHL3 binding and enhancers rearrange as epidermal keratinocytes transition between functional states. *PLoS Genet* **13**, e1006745, doi:10.1371/journal.pgen.1006745 (2017).

14 Curry, E. *et al.* Genes Predisposed to DNA Hypermethylation during Acquired Resistance to Chemotherapy Are Identified in Ovarian Tumors by Bivalent Chromatin Domains at Initial Diagnosis. *Cancer Res* **78**, 1383-1391, doi:10.1158/0008-5472.CAN-17-1650 (2018).

15 Zhang, Q. *et al.* Mdig promotes oncogenic gene expression through antagonizing repressive histone methylation markers. *Theranostics* **10**, 602-614, doi:10.7150/thno.36220 (2020).

16 Andersson, R. *et al.* An atlas of active enhancers across human cell types and tissues. *Nature* **507**, 455-461, doi:10.1038/nature12787 (2014).

17 Jiang, K. *et al.* Disease-Associated Single-Nucleotide Polymorphisms From Noncoding Regions in Juvenile Idiopathic Arthritis Are Located Within or Adjacent to Functional Genomic Elements of Human Neutrophils and CD4+ T Cells. *Arthritis Rheumatol* **67**, 1966-1977, doi:10.1002/art.39135 (2015).

18 Limbach, M. *et al.* Epigenetic profiling in CD4+ and CD8+ T cells from Graves' disease patients reveals changes in genes associated with T cell receptor signaling. *J Autoimmun* **67**, 46-56, doi:10.1016/j.jaut.2015.09.006 (2016).

19 Gao, X. *et al.* Thyroid hormone receptor beta and NCOA4 regulate terminal erythrocyte differentiation. *Proc Natl Acad Sci U S A* **114**, 10107-10112, doi:10.1073/pnas.1711058114 (2017).

20 Yao, X. *et al.* VHL Deficiency Drives Enhancer Activation of Oncogenes in Clear Cell Renal Cell Carcinoma. *Cancer Discov* **7**, 1284-1305, doi:10.1158/2159-8290.CD-17-0375 (2017).
