# Supplementary material for: Integrative pan cancer analysis reveals epigenomic variation in cancer type and cell specific chromatin domains
Source: Nat Commun. 2021 Mar 3;12:1419. doi: 10.1038/s41467-021-21707-1 (PMC7930052; doi:10.1038/s41467-021-21707-1)
Supplement: Supplementary file 2 — Description of Additional Supplementary Files [file 41467_2021_21707_MOESM2_ESM.pdf]

Description of Additional Supplementary Files

File Name: Supplementary Data 1
Description: List of NCI-60 cell lines.

File Name: Supplementary Data 2
Description: Cellminer expression of DNA methyltransferases in NCI-60 cells.

File Name: Supplementary Data 3
Description: H3K4me3 cancer-type specific GO terms.

File Name: Supplementary Data 4
Description: TUSON explorer list of all genes with TSG-like properties

File Name: Supplementary Data 5
Description: GREAT GO analysis of super-enhancers.

File Name: Supplementary Data 6
Description: STR profiling of the NCI-60 cell lines.

File Name: Supplementary Data 7
Description: ChIP-Seq samples GSE143653.
