# Supplementary material for: Integrative pan cancer analysis reveals epigenomic variation in cancer type and cell specific chromatin domains
Source: Nat Commun. 2021 Mar 3;12:1419. doi: 10.1038/s41467-021-21707-1 (PMC7930052; doi:10.1038/s41467-021-21707-1)
Supplement: Supplementary file 10 — Reporting Summary [file 41467_2021_21707_MOESM10_ESM.pdf]

nature research

Corresponding author(s): Benjamin Kidder

Last updated by author(s): Jan 18, 2021

Reporting Summary

Nature Research wishes to improve the reproducibility of the work that we publish. This form provides structure for consistency and transparency in reporting. For further information on Nature Research policies, see our Editorial Policies and the Editorial Policy Checklist.

Statistics

For all statistical analyses, confirm that the following items are present in the figure legend, table legend, main text, or Methods section.

n/a	Confirmed	
☐	☒	The exact sample size (*n*) for each experimental group/condition, given as a discrete number and unit of measurement
☐	☒	A statement on whether measurements were taken from distinct samples or whether the same sample was measured repeatedly
☐	☒	The statistical test(s) used AND whether they are one- or two-sided<br>*Only common tests should be described solely by name; describe more complex techniques in the Methods section.*
☒	☐	A description of all covariates tested
☐	☒	A description of any assumptions or corrections, such as tests of normality and adjustment for multiple comparisons
☐	☒	A full description of the statistical parameters including central tendency (e.g. means) or other basic estimates (e.g. regression coefficient) AND variation (e.g. standard deviation) or associated estimates of uncertainty (e.g. confidence intervals)
☐	☒	For null hypothesis testing, the test statistic (e.g. *F*, *t*, *r*) with confidence intervals, effect sizes, degrees of freedom and *P* value noted<br>*Give P values as exact values whenever suitable.*
☒	☐	For Bayesian analysis, information on the choice of priors and Markov chain Monte Carlo settings
☒	☐	For hierarchical and complex designs, identification of the appropriate level for tests and full reporting of outcomes
☒	☐	Estimates of effect sizes (e.g. Cohen's *d*, Pearson's *r*), indicating how they were calculated

Our web collection on statistics for biologists contains articles on many of the points above.

Software and code

Policy information about availability of computer code

Data collection: Software was not used for data collection.

Data analysis: Bowtie2 (v2.3.4.3) was used for aligning ChIP-Seq reads. SICER (v1.03) was used for ChIP-Seq peak calling. ChIP-Seq density calculations were performed as described by Hu et al (Methods in Molecular Biology, 2014). C++ programs to convert a SAM formatted file to a BED6 format from bowtie2 (Sam2Bed6_Bowtie2), to remove redundant reads from a BED6 file (RemoveRedundantReads), and to convert a BED6 file to a BEDGraph file (GenerateRPBMBasedSummary) were described previously by Hu et al (Methods in Molecular Biology, 2014). ChromHMM v1.2 was used to identity chromatin states. The R libraries GenomicScores (v1.4.1), regioneR (v1.12.0), phastCons100way.UCSC.hg19, and GenomicRanges (v1.32.7) were used to calculate conservation scores. Motif discovery was performed using ENCODE motifs (v1.3). Super enhancers were identified using HOMER (v4.10.3). NCBI DAVID (v6.8) was used to functionally annotate genes and evaluated by semantic analysis using GoSemSim (v2.6.2).

For manuscripts utilizing custom algorithms or software that are central to the research but not yet described in published literature, software must be made available to editors and reviewers. We strongly encourage code deposition in a community repository (e.g. GitHub). See the Nature Research guidelines for submitting code & software for further information.

Data

Policy information about availability of data

All manuscripts must include a data availability statement. This statement should provide the following information, where applicable:

- Accession codes, unique identifiers, or web links for publicly available datasets
- A list of figures that have associated raw data
- A description of any restrictions on data availability

The sequencing data from this study have been submitted to the NCBI Gene Expression Omnibus (GEO) (http://www.ncbi.nlm.nih.gov/geo) under accession no.

GSE143653. A description of the ChIP-Seq samples can be found in Supplementary Data 7.
Publicly available H3K27ac ChIP-Seq datasets analyzed in this study: GSM1003459, GSM1027287, GSM1003559, GSM1102782, GSM2152595, GSM772859, GSM999004, GSM1102781, GSM1847878, GSM999000, GSM999001, GSM2741449, GSM773004, GSM1027288, GSM2527658, GSM1633870, GSM733763, GSM2698422, GSM2293347, GSM906395, GSM1013123, GSM956009, GSM4250668, GSM2699699, GSM910559, GSM1666386, GSM1662338, GSM2698631.
Publicly available H3K4me3 ChIP-Seq datasets analyzed in this study: GSM1427065, GSM1647618, GSM1666384, GSM1782766, GSM1874929, GSM2035818, GSM2067930, GSM2736544, GSM3011841, GSM3011844, GSM3011847, GSM3011850, GSM4315283, GSM529959, GSM529964, GSM529966, GSM529967, GSM621457, GSM621665, GSM733720, GSM733747, GSM773041, GSM883691, GSM883692, GSM945276, GSM947523, GSM971341, SRR11600891, SRR11600898.

The source data underlying Figures 1a, 1b, 1c, 1d, 1e, 2a, 2b, 3a, 3b, 3c, 3d, 3f, 3g, 3h, 4a, 4b, 4c, 4d, 4e, 4f, 4g, 4h, 5a, 5b, 5c, 5d, 5e, 5f, 5g, 5h, 5i, 5j, 6b, 6c, 6d, 6e, 6f, 6g, 6h, 6i, 7a, 7b, 7e, 7f, 7g, 7h, and supplementary Figures S2, S3a, S3b, S3c, S3d, S3e, S4a, S4b, S5a, S5b, S5c, S6, S7, S8, S9, S11, S12a, S12b, S12c, S13, S14, S16a, S16b, S17a, S17b, S20, S22A, S22B, S23A, S23B, S24A, S24B, S25, S26A, S26B, S27, S28, S29, S31A, S31B, S36A, and S36B are provided as a Source Data file.

Field-specific reporting

Please select the one below that is the best fit for your research. If you are not sure, read the appropriate sections before making your selection.

☒ Life sciences ☐ Behavioural & social sciences ☐ Ecological, evolutionary & environmental sciences

For a reference copy of the document with all sections, see nature.com/documents/nr-reporting-summary-flat.pdf

Life sciences study design

All studies must disclose on these points even when the disclosure is negative.

Sample size	Cell numbers selected for the H3K4me3, H3K27ac, H3K9me3, and H4K20me3 ChIP-Seq experiments are typical for the field. Statistical tests were not used to predetermine sample size.
Data exclusions	Data was not excluded.
Replication	We have generated biological duplicate H3K4me3, H3K27ac, H3K9me3, and H4K20me3 ChIP-Seq datasets for all cell lines in the NCI-60 panel. All results were highly consistent.
Randomization	Randomization was not necessary in this in vitro experiment. This study did not use animals and human participants. It is not relevant to this study.
Blinding	The investigators were not blinded during acquisition of data and analysis. This study involved in vitro experiments and therefore no blinding is required. This study did not use animals and human participants. It is not relevant to this study.

Reporting for specific materials, systems and methods

We require information from authors about some types of materials, experimental systems and methods used in many studies. Here, indicate whether each material, system or method listed is relevant to your study. If you are not sure if a list item applies to your research, read the appropriate section before selecting a response.

Materials & experimental systems

n/a	Involved in the study	
☐	☒	Antibodies
☐	☒	Eukaryotic cell lines
☒	☐	Palaeontology and archaeology
☒	☐	Animals and other organisms
☒	☐	Human research participants
☒	☐	Clinical data
☒	☐	Dual use research of concern

Methods

n/a	Involved in the study	
☐	☒	ChIP-seq
☒	☐	Flow cytometry
☒	☐	MRI-based neuroimaging

Antibodies

Antibodies used	Anti-H3K4me3 (17-614, Millipore)<br>Anti-H3K27ac (ab4729, Abcam)<br>Anti-H3K9me3 (ab8898, Abcam)<br>Anti-H4K20me3 (ab9053, Abcam)
Validation	Validation of commercial antibodies was performed by the respective companies. The antibodies have been confirmed to be ChIP grade on the manufacturer's specification sheets.

Eukaryotic cell lines

Policy information about cell lines

Cell line source(s)	The NCI-60 cell lines were obtained from the NCI DTP Tumor Repository.
Authentication	The NCI DTP Tumor Repository performed Applied Biosystems AmpFLSTR Identifiler testing with PCR amplification to confirm consistency with the published Identifiler STR profile for each of the NCI-60 cell lines (Supplementary Data 6).
Mycoplasma contamination	The cells were not tested for mycoplasma contamination.
Commonly misidentified lines (See ICLAC register)	No commonly misidentified cell lines were used in this study.

ChIP-seq

Data deposition

☒ Confirm that both raw and final processed data have been deposited in a public database such as GEO.

☒ Confirm that you have deposited or provided access to graph files (e.g. BED files) for the called peaks.

Data access links *May remain private before publication.*	https://www.ncbi.nlm.nih.gov/geo/query/acc.cgi?acc=GSE143653
Files in database submission	We have provided a supplemental table with descriptions of the samples submitted to GEO (Supplementary Data 7).
Genome browser session (e.g. UCSC)	NA

Methodology

Replicates	We generated biological duplicate H3K4me3, H3K27ac, H3K9me3, and H4K20me3 ChIP-Seq datasets for the NCI-60 panel.
Sequencing depth	Single-end and paired-end sequencing was performed for ChIP-Seq samples. ~20-30 million reads were generated for each ChIP-Seq sample.
Antibodies	Anti-H3K4me3 (17-614, Millipore)<br>Anti-H3K27ac (ab4729, Abcam)<br>Anti-H3K9me3 (ab8898, Abcam)<br>Anti-H4K20me3 (ab9053, Abcam)
Peak calling parameters	SICER (v1.03) was used for ChIP-Seq peak calling with a window size setting of 200 bps, a gap setting of 400 bps and a FDR setting of 0.001
Data quality	SICER (v1.03) was used for ChIP-Seq peak calling, using a stringent FDR < 0.001.
Software	Bowtie2 (v2.3.4.3) was used for aligning ChIP-Seq reads. SICER (v1.03) was used for ChIP-Seq peak calling.
